# Synthesis, Characterisation and Mechanism of Action of Anticancer 3-Fluoroazetidin-2-ones

**DOI:** 10.3390/ph15091044

**Published:** 2022-08-24

**Authors:** Azizah M. Malebari, Gabriela Duffy Morales, Brendan Twamley, Darren Fayne, Mohemmed Faraz Khan, Eavan C. McLoughlin, Niamh M. O’Boyle, Daniela M. Zisterer, Mary J. Meegan

**Affiliations:** 1 Department of Pharmaceutical Chemistry, College of Pharmacy, King Abdulaziz University, Jeddah 21589, Saudi Arabia; 2 Trinity Biomedical Sciences Institute, School of Pharmacy and Pharmaceutical Sciences, Trinity College Dublin, 152-160 Pearse Street, Dublin 2, D02 R590 Dublin, Ireland; 3 School of Chemistry, Trinity College Dublin, Dublin 2, D02 PN40 Dublin, Ireland; 4 Trinity Biomedical Sciences Institute, School of Biochemistry and Immunology, Trinity College Dublin, 152-160 Pearse Street, Dublin 2, D02 R590 Dublin, Ireland

**Keywords:** combretastatin A-4, antiproliferative activity, fluorinated β-lactams, Reformatsky reaction, inhibition of tubulin polymerisation, breast cancer

## Abstract

The stilbene combretastatin A-4 (CA-4) is a potent microtubule-disrupting agent interacting at the colchicine-binding site of tubulin. In the present work, the synthesis, characterisation and mechanism of action of a series of 3-fluoro and 3,3-difluoro substituted β-lactams as analogues of the tubulin-targeting agent CA-4 are described. The synthesis was achieved by a convenient microwave-assisted Reformatsky reaction and is the first report of 3-fluoro and 3,3-difluoro β-lactams as CA-4 analogues. The β-lactam compounds 3-fluoro-4-(3-hydroxy-4-methoxyphenyl)-1-(3,4,5-trimethoxy phenyl)azetidin-2-one **32** and 3-fluoro-4-(3-fluoro-4-methoxyphenyl)-1-(3,4,5-trimethoxyphenyl)azetidin-2-one) **33** exhibited potent activity in MCF-7 human breast cancer cells with IC_50_ values of 0.075 µM and 0.095 µM, respectively, and demonstrated low toxicity in non-cancerous cells. Compound **32** also demonstrated significant antiproliferative activity at nanomolar concentrations in the triple-negative breast cancer cell line Hs578T (IC_50_ 0.033 μM), together with potency in the invasive isogenic subclone Hs578Ts(i)8 (IC_50_ = 0.065 μM), while **33** was also effective in MDA-MB-231 cells (IC_50_ 0.620 μM). Mechanistic studies demonstrated that **33** inhibited tubulin polymerisation, induced apoptosis in MCF-7 cells, and induced a downregulation in the expression of anti-apoptotic Bcl2 and survivin with corresponding upregulation in the expression of pro-apoptotic Bax. In silico studies indicated the interaction of the compounds with the colchicine-binding site, demonstrating the potential for further developing novel cancer therapeutics as microtubule-targeting agents.

## 1. Introduction

Breast cancer is the most prevalent cancer diagnosed in women, with 2.3 million women diagnosed with breast cancer and 685,000 deaths globally in 2020 [1,2], and is recognised as the leading cause of cancer-related deaths in women. Many successful anticancer agents with different biological targets are used clinically to treat breast cancer [3,4]. Approximately 70–80% of breast cancers are hormone-dependent; the majority are identified as estrogen receptor-positive (ER+) cancers which also express the progesterone receptor (ER+/PR+). The estrogen receptor is directly targeted by selective estrogen receptor modulators (e.g., tamoxifen) and selective estrogen receptor degraders (e.g., fulvestrant). The aromatase inhibitors anastrozole, letrozole and exemestane block a key step in estrogen biosynthesis and are effective clinical adjuvant therapies. Triple-negative breast cancer (TNBC) is an aggressive breast cancer subtype which lacks the estrogen receptor (ER) and progesterone receptor (PR) expression and also lacks overexpression of the human epidermal growth factor receptor 2 (HER2). TNBCs account for 15–20% of diagnosed breast cancers in the US, with mortality of 50% and median overall survival of less than 2 years [5], and are associated with younger age at diagnosis [6]. Patient responses to endocrine and HER2 targeted therapies in TNBCs are poor, and effective targeted therapy has not been well established [7].

Microtubules play a vital role in cell division and mitosis, and their dynamic nature makes them a significant and intensively investigated molecular target for the design of novel anticancer agents. Microtubule-targeting agents (MTAs) suppress microtubule dynamics, inhibit microtubule function and induce apoptosis. The microtubule-stabilising drugs paclitaxel, docetaxel and the epothilone ixabepilone are used in patients with metastatic breast cancer (MBC), together with the mitotic inhibitor eribulin, which is a synthetic macrocyclic ketone analogue of the marine natural product halichondrin B [8,9].

The most recent drugs approved by the European Medicines Agency (2015–2021) for the treatment of breast cancer (including HER2-targeted drugs, CDK 4/6 inhibitors, PIK3CA inhibitors, PARP inhibitors, immunotherapy drugs and antibody–drug conjugates (ADCs)) were recently reviewed by Duranti et al. [10]. Tucatinib is an orally bioavailable tyrosine kinase inhibitor that is approved for HER2-positive MBC [11], while the phosphoinositide-3-kinase (PI3Kα) inhibitor alpelisib is approved for HER2-negative, PIK3CA-mutated advanced breast cancer [12,13]. The immunotherapy drug pembrolizumab is recently approved for early-stage, triple-negative breast cancer (TNBC) [14], while the human epidermal growth factor receptor (HER2) inhibitor margetuximab-cmkb [15] and the antibody–drug conjugate Fam-trastuzumab deruxtecan (HER2-directed antibody which is linked to a topoisomerase inhibitor) also gained FDA approval for the treatment of HER2-positive MBC [16]. In addition, the ADC sacituzumab govitecan (TROP2-targeted antibody linked with a topoisomerase I inhibitor) suitable for previously treated metastatic TNBC [17] and the ADC ladiratuzumab vedotin (a LIV-1-targeted antibody linked to the potent microtubule-disrupting agent monomethyl auristatin E (MMAE)) have gained approval for locally advanced or metastatic TNBC [18].

**Figure 1:**
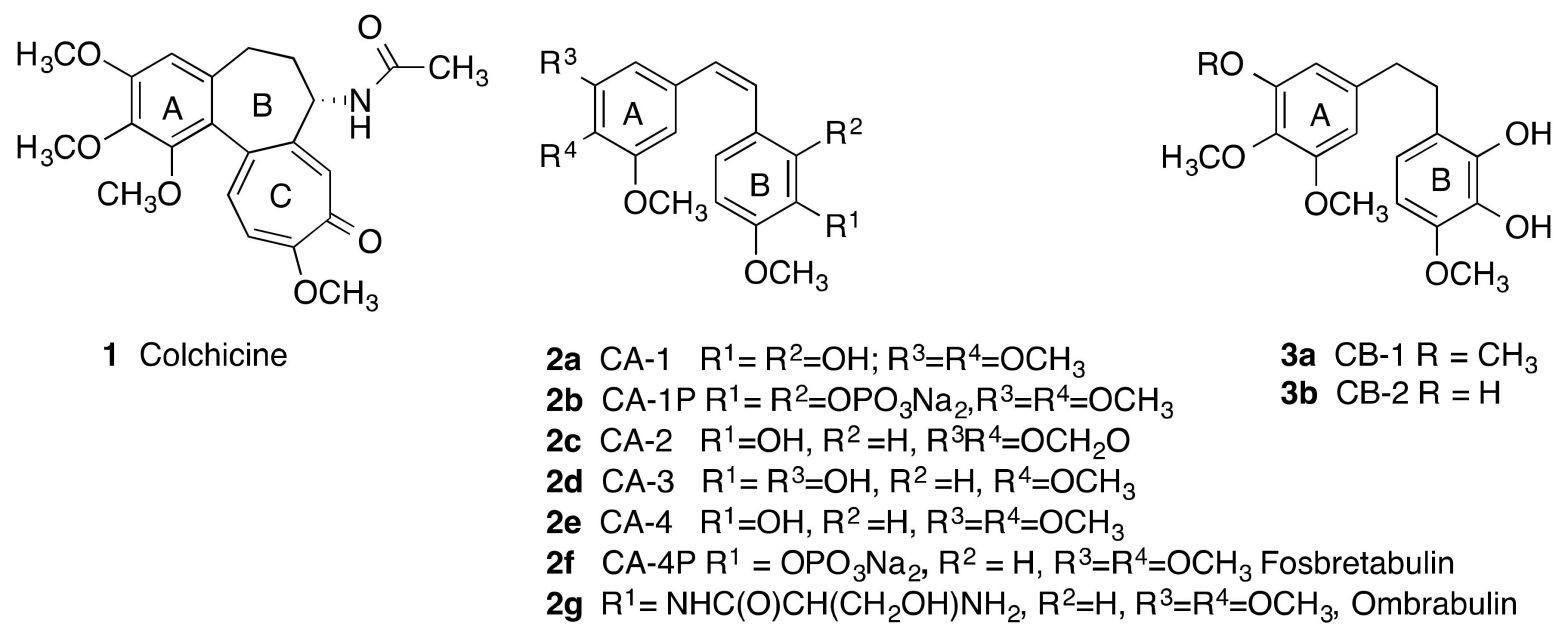
Colchicine (**1**), combretastatins CA-1 (**2a**), CA-1P (**2b**), CA-2 (**2c**), CA-3 (**2d**), CA-4 (**2e**), CA-4P (fosbretabulin) (**2f**), ombrabulin (**2g**), CB-1 (**3a**) and CB-2 (**3b**).

Colchicine **1** (Figure 1) isolated from *Colchicum autumnale* was the first agent identified to bind at a specific site of tubulin identified as the colchicine domain [19]. Many structurally diverse compounds were subsequently discovered which bind to tubulin at the colchicine-binding site and inhibit tubulin polymerisation, resulting in cell cycle arrest followed by apoptotic cell death [20]. The stilbene combretastatin A-4 (CA-4, **2e**, Figure 1), isolated from the African bush willow tree *Combretum caffrum*, contains two substituted aryl rings, A and B, which are linked by an olefinic bridge [21,22]. CA-4 is a potent inhibitor of tumour cell proliferation, migration and invasion; in addition, CA-4 significantly promotes cell apoptosis, inhibits tubulin polymerisation and disrupts microtubule dynamics. Examples of the combretastatin group **2a**–**2e** are illustrated in Figure 1, including combretastatins CA-1 (**2a**), CA-2 (**2c**), CA-3 (**2d**) and CA-4 (**2e**) and the combretastatin B-series (CB-1 (**3a**) and CB-2 (**3b**)). However, there are many limitations associated with CA-4 such as poor water solubility and chemical instability (by isomerisation to more stable inactive *E*-isomer during storage and administration), together with phase I and phase II metabolic transformations [22]. The low aqueous solubility has been overcome by the development of the water-soluble phosphate prodrugs CA-1P, **2b,** and CA-4P, fosbretabulin, **2f** [23,24,25], and the amino prodrug ombrabulin (AVE8062) (**2g**), a synthetic water-soluble vascular disrupting combretastatin analogue [26] (Figure 1). The isomerisation instability has been overcome by the replacement of the double bond of CA-4 with different heterocycles which lock the *E*-configuration, leading to the discovery and development of combretastatin analogues [23,27,28,29]. CA-4 and derivatives continue to be of interest in cancer cell biology as potent inhibitors of angiogenesis and cell proliferation and inducers of apoptosis and are known to modulate an extensive network of cell signalling systems [21,30]. Among the heterocyclic structures reported, the β-lactam ring has been explored in the design of CA-4 analogues as it provides a rigid scaffold for the CA-4 aryl rings, preventing the undesired conversion to the inactive *trans* isomer while maintaining the essential *cis* geometry between rings A and B of CA-4 and resulting in potent antimitotic compounds [31,32,33,34]. β-Lactam derivatives are versatile and attractive chemical scaffolds for drug discovery [35,36,37], and the β-lactam structure is a useful template for the design of novel anticancer agents [38].

**Figure 2:**
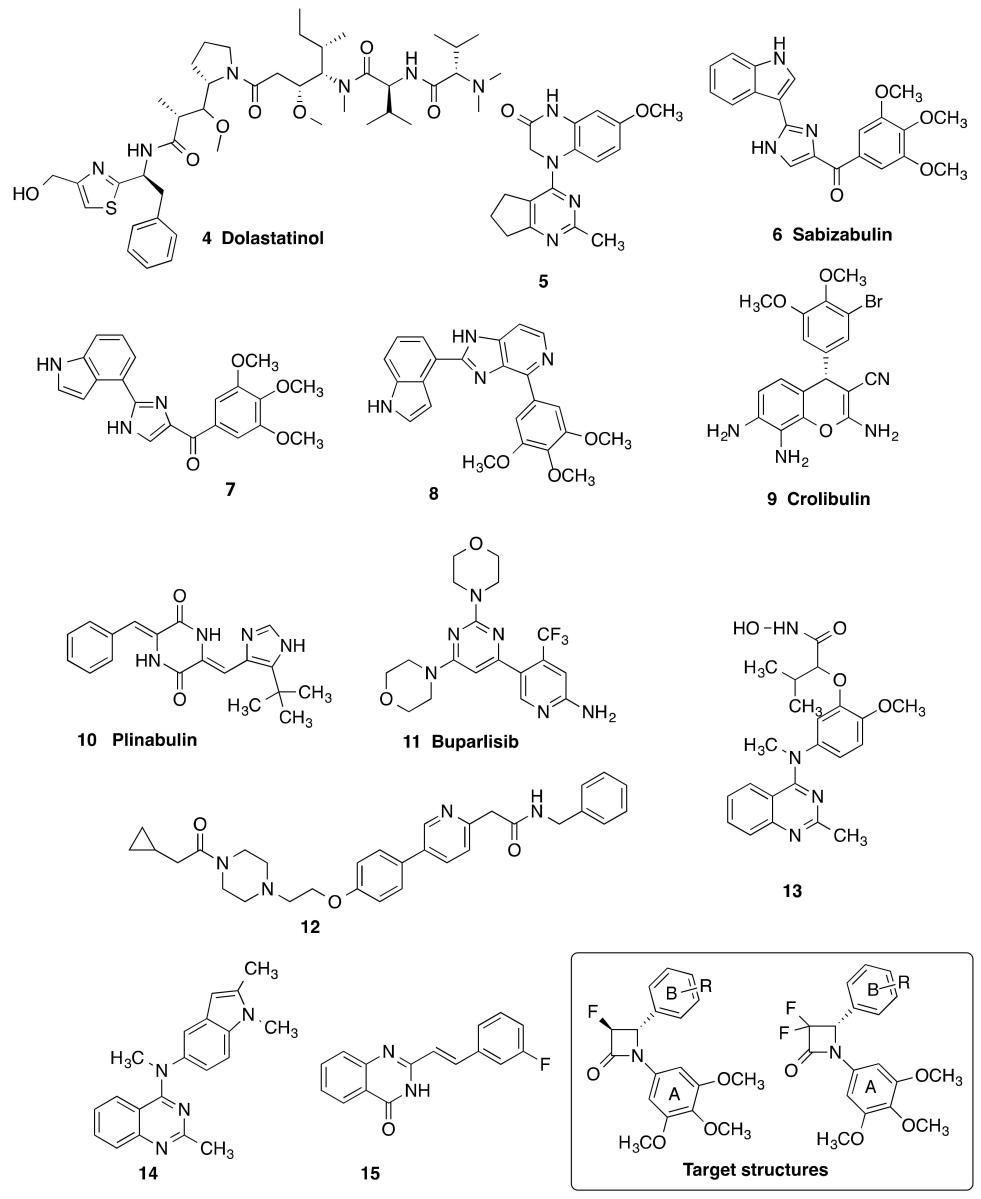
Microtubule-targeting agents **4**–**15** and target structures.

Microtubule-targeting agents (MTAs) are among the most frequently prescribed antitumour chemotherapeutic agents. However, despite the benefits of microtubule-targeting drugs such as taxol and the vinca alkaloids in the clinic, the development of drug resistance and dose-limiting toxicities restrict their clinical efficacy [39]. Recently reported structurally diverse tubulin-targeting compounds with potent activities include the peptide dolastatinol **4** (Figure 2) [40] and the hydroquinoxalinone **5**, binding at the colchicine site [41]. The *bis*-indole sabizabulin **6** (ABI-231, Veru-111) is a potent, orally bioavailable tubulin inhibitor in clinical trials for metastatic castration-resistant prostate cancer. Interaction of **6** and related analogue **7** with the colchicine-binding site is confirmed by X-ray crystallography [42,43]. Additionally, the antiviral activity of sabizabulin against SARS-CoV-2 is reported. Sabizabulin binds to viral tubulin, disrupting the intracellular transport of the SARS-CoV-2 virus together with anti-inflammatory effects [44]. The indolylimidazopyridine **8** is a microtubule-depolymerising agent in metastatic melanomas and P-gp-mediated multidrug-resistant cancers [45]. A phase I/II clinical trial for anaplastic thyroid cancer was completed for the 4-aryl-4*H*-chromene crolibulin **9** which interacts with tubulin at the colchicine-binding site [46]. The orally active diketopiperazine plinabulin **10** also targets the colchicine-binding site and disrupts tumour growth [47], while the phosphoinositide 3-kinase (PI3K) inhibitor buparlisib **11** disrupts microtubule polymerisation as an off-target effect, and has been assessed in metastatic TNBC [48]. Preclinical studies have suggested that CBSIs are effective in suppressing the overexpression of tubulin isotypes and can prevent P-gp-, MRP1- and MRP2-mediated drug resistance [28]. Tubulin inhibitors as dual targeting small molecules for cancer therapy [49] have been reported; e.g., the dual-targeting inhibitor of tubulin and Src kinase **12** [50] and the hydroxamic acid based microtubule destabilising agent **13** [51]; **14** synergistically targets both the tubulin colchicine site and aryl hydrocarbon receptor [52], and **15** is a dual inhibitor of poly(ADP-ribose) polymerase-1 (PARP-1) and tubulin [53] (Figure 2).

The introduction of fluorine can enhance the potency and target selectivity of a drug by affecting properties such as pKa, lipophilicity, hydrophobic interactions, conformation, membrane permeability and P-gp recognition while oral bioavailability and half-life can also be improved [54]. More than 340 marketed drugs (20% of the market) contain at least one fluorine atom [55]. Fluorinated analogues of CA-1 and CA-4 demonstrate varying effects on cancer cell cytotoxicity and tubulin binding [56]. We have previously reported the synthesis of β-lactams containing various C3 β-lactam ring substituents with antiproliferative activity against breast cancer MCF-7 cells [32,33,34,57].

Synthetic methods for monocyclic β-lactams have been extensively reviewed [58,59,60]. The most popular methods for the construction of β-lactams are ketene/imine cycloadditions (Staudinger reaction), ester or amide enolate–imine condensations and [2 + 2] cycloadditions of isocyanates with vinyl ethers. Ligand-controlled transition metal catalysis such as the alkyne–nitrone Kinugasa reaction and rhodium-mediated diazo activation/C–H bond insertion methods have been developed [35,61]. Structurally diverse β-lactam compounds have attracted considerable interest as core structures of antibiotics and as synthons for chiral β-amino acids [62]. 3-Fluoro-β-lactams have been used in the preparation of fluorinated β-amino acids as core intermediates for more complex molecules such as fluoro analogues of taxol [63,64].

The objective of this research is the synthesis of a series of novel β-lactam bridged CA-4 analogues with 3-fluoro and 3,3-difluoro substituents at the 3-position on the β-lactam ring (Figure 2, target structures) and the investigation of the antiproliferative effects of the compounds in MCF-7 breast cancer cells and in TNBC cell lines. We also aimed to identify the mechanism of action of these compounds as potential antitubulin agents. In addition, we now investigate possible fluorophilic binding sites on the colchicine-binding site which may contribute to stabilisation of the drug–receptor complex [65]. The 3-fluoro β-lactams (**14**–**23**) and 3,3-difluoro β-lactams (**24**–**33**) identified for study contain the 3,4,5-trimethoxyphenyl ring A (required for potent activity of CA-4), together with selected ring B substituents [33,34]. These compounds will facilitate the investigation of the effect of the introduction of the 3-fluoro substituent on the anticancer activity in MCF-7 breast cancer cells and the tubulin binding effects of the β-lactam CA-4 analogues.

## 2. Results and Discussion

### 2.1. Chemistry

**Scheme 1:**
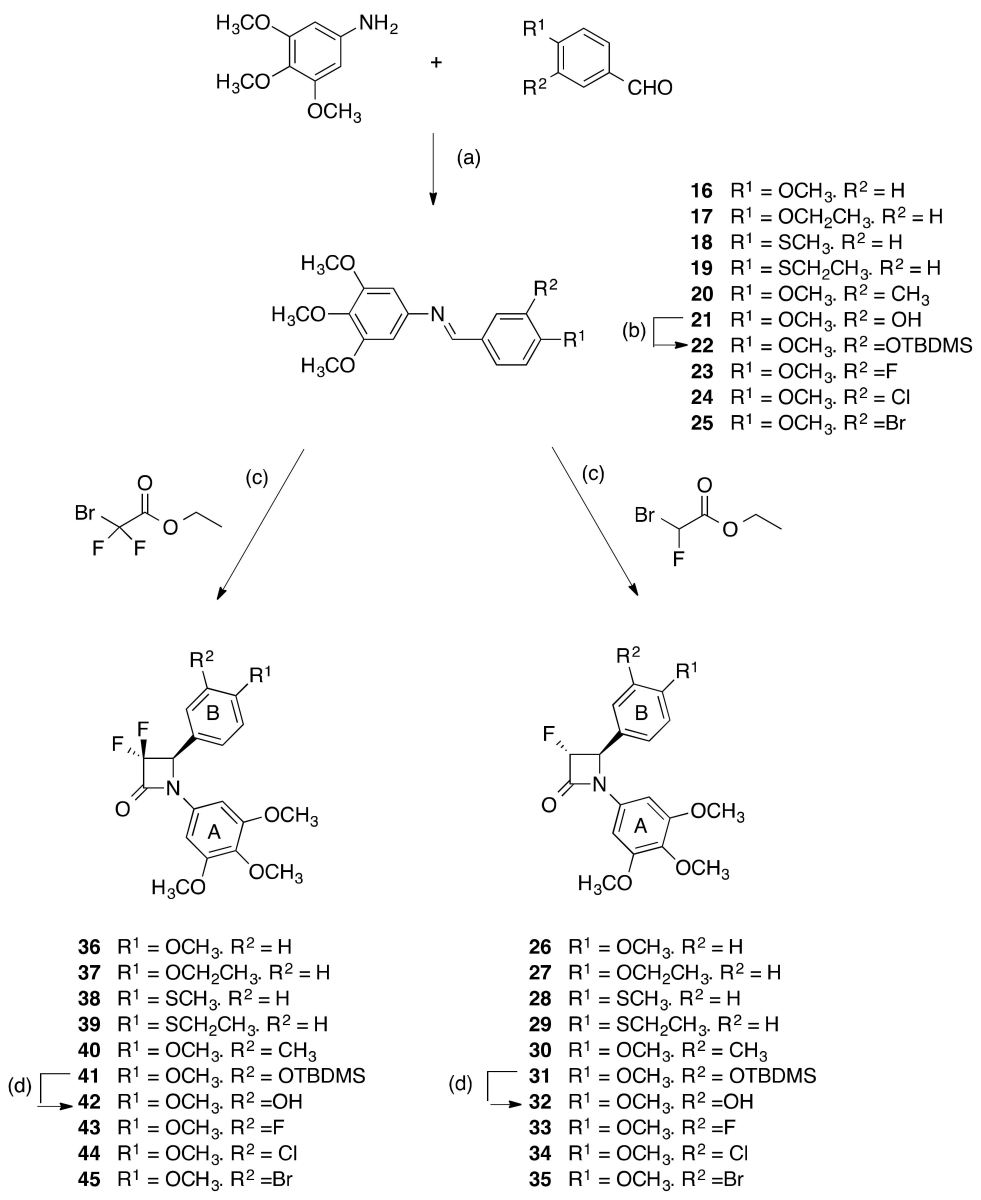
Synthesis of 2-azetidinones **26**–**45**. Reagents and conditions: (**a**) EtOH, reflux, 4 h, 75–91%; (**b**) TBDMSCl, DBU, CH_2_Cl_2_, 20 °C, until reaction complete as indicated by TLC, 52%; (**c**) Zn dust, TMSCl, 40 °C, 15 min, then 100 °C, 2 min, microwave, C_6_H_6_, 100 °C, 30 min, microwave (**26**–**31**, **33**–**35**, 6–58%; **36**–**41**, **43**–**45**, 13–65%); (**d**) TBAF, THF, 0 °C, 15 min (**32**, 18%; **42**, 21%). Products obtained as racemic mixtures, one enantiomer illustrated.

In the present work, the preparation of a series of 3-fluoro and 3,3-difluoro substituted β-lactams **26**–**45** was achieved using the microwave-assisted Reformatsky reaction of the appropriate imines and ethyl bromofluoroacetate or ethyl bromodifluoroacetate, respectively, in the presence of trimethylchlorosilane and zinc dust (Scheme 1). The imines **16**–**21** and **23**–**25** required for the synthetic procedures were obtained by condensation of 3,4,5-trimethoxyaniline with the appropriately substituted benzaldehyde in 75–91% yields (Scheme 1). Phenol **21** was protected as the *tert*-butyldimethylsilyl (TBDMS) ether **22** by reaction with *tert*-butyldimethylsilyl chloride. The characteristic imine proton signal was observed in the ^1^H NMR spectra of these imines, e.g., for compound **22**, as the singlet at δ 8.51, and the aromatic ^19^F signal was observed at δ −134.3 in compound **23** (see Supplementary Materials). X-ray crystallography confirmed the *E* configuration of the imines **18** and **23** (Table 1), with centrosymmetric and monoclinic packing structure observed for both compounds. The N1-C2 imine bond lengths were 1.2788 (14) Å and 1.2790 (12) Å respectively, with typical bond angles of 117.58° (C2N1C14) and 123.10° (N1C14C15) for compound **18** and 117.64° (C2N1C11) and 122.87° (N1C2C3) for compound **23**. The torsion angles for N=C-C-phenyl ring were observed as −177.67 (11)° and 171.67 (9)° for compounds **18** and **23**, respectively, with the torsion angles for the C=N-C- phenyl ring measured as −140.42 (11)° and 140.51 (9)°, respectively.

The 3-fluoro-β-lactams **26**–**31** and **33**–**35** were obtained by microwave-assisted Reformatsky reaction of the imines **16**–**21** and **23**–**25**, respectively, with ethyl bromofluoroacetate in a short reaction time (30 min) in moderate yield (6–58%) after purification via flash column chromatography (Scheme 1). Deprotection of the TBDMS ether **31** with tetrabutylammonium fluoride (TBAF) afforded the phenol **32** in 18% yield. For compounds **26**–**35**, the β-lactam carbonyl group was confirmed from the IR spectrum (ν 1736–1762 cm^−1^). In the ^1^H NMR spectrum of compounds **31** and **32**, H-3 was observed at 5.14 ppm and 5.11 ppm, respectively; geminal coupling with the 3-fluoro substituent of the β-lactam ring to H3 was demonstrated with ^3^*J* fluorine H-F coupling constants of 47 Hz and 48 Hz for **31** and **32**, respectively. Due to ^2^*J* fluorine coupling, the typical β-lactam doublet for H3 resonates as an apparent double doublet with a second ^3^*J* coupling of 1.7 Hz and 2.6 Hz for H_3_ of **31** and **32**, respectively, to the adjacent H_4_ of the β-lactam ring. The characteristic coupling constant indicates that the *trans* isomer is isolated exclusively in this reaction for the series of compounds. Interestingly, we previously obtained a 2:1 *cis*/*trans* mixture of 3-methylazetidin-2-ones by reaction of ethyl-2-bromopropionate with the imine under similar conditions [57]. For both compounds **31** and **32**, H_4_ resonates also as an apparent double doublet with ^3^*J* coupling to both the 3-fluoro substituent and adjacent *trans* H_3_. ^3^*J* fluorine coupling constant values for H_4_ are narrower than values for geminal fluorine coupling observed for H_3_ with values of 25 and 26 Hz for **31** and **32**, respectively. Additionally, interesting carbon–fluorine coupling is observed on the ^13^C spectrum for both **31** and **32**, at C_2_, C_3_ and C_4_ of the β-lactam ring. For compound **32**, C_3_ resonates much further downfield compared to C_4_ due to the electron-withdrawing effect of the 3-fluoro atom (δ 95.2 ppm and 58.8 ppm, respectively) as an apparent double doublet with carbon–fluorine coupling constant values of 75 and 26 Hz, respectively. The carbonyl carbon (C_2_) of the β-lactam ring for **32** resonates as a doublet also coupling to the 3-fluoro substituent with a *J* value of 31 Hz. The characteristic ^19^F NMR spectrum confirmed the presence of the 3-fluoro substituent in compound **31** at δ −203.7 and **32** at δ −203.6.

The 3,3-difluoro-β-lactams **36**–**41** and **43**–**45** were similarly prepared by reaction of ethyl bromodifluoroacetate with the imines **16**–**20** and **22**–**25**, respectively, in 13–65% yield (Scheme 1). Deprotection of the TBDMS ether **41** with TBAF afforded the phenol **42** in 21% yield. In the ^1^H NMR spectrum of 3,3-difluoro compound **37**, the H4 signal was observed as a double doublet δ 5.29 ppm (*J* = 12.20 Hz and 1.64 Hz). In the ^13^C NMR spectrum of compound **37**, the difluoro-substituted C3 was observed at δ 121.47 ppm (further downfield compared with C-3 of monofluoro-substituted β-lactam, e.g., for compound **31** where C-3 is found at δ 95.2 ppm), and while the resonance of C4 occurred at δ 63.59 ppm. All products were obtained as racemic mixtures, with one enantiomer illustrated (Scheme 1).

### 2.2. X-ray Structural Study for 3-Fluoro and 3,3-Difluoro-β-lactams ***33*** and ***43***

X-ray crystallographic analysis of the 3-fluoro β-lactam **33** and 3,3-difluoro β-lactam **43** confirmed the stereochemical assignments (Table 2, Table 3 and Table 4). The β-lactam ring in both compounds **33** and **43** is observed with a conformationally restricted scaffold for the planar aryl rings A and B usually required for characteristic interaction with the colchicine-binding site of tubulin. As shown in Table 2, the X-ray structures demonstrated a rigid configuration for both β-lactams with rings A and B not coplanar. The torsional angles (ring A/B) observed for compounds **33** and **43** are 62.3° and −76.6° respectively (Table 4) indicating that compound **33** is more comparable with the related ring A/B torsional angles observed for colchicine [19] and CA-4 [66,67] reported as 53° and 55° respectively. The torsional angle value for ring B and the 3-fluoro group of compound **33** is -119.43° which is similar to our previously reported 3-hydroxy β-lactams (117.0°) and 3-chloro β-lactams (114.4°) [33] (Table 4). In contrast, the torsional angle value increased to 130.71° for 3,3-difluoro β-lactam **43** suggesting that this may modulate the tubulin activity observed for this compound. The β-lactam C=O bond lengths of 1.2134 (15) Å and 1.2012 (19) Å are observed for compounds **33** and **43** respectively, in agreement with carbonyl bond lengths reported for monocyclic β-lactams [68]. The strained *β*-lactam four-membered ring differs from a normal amide resulting in decreased amide resonance. Monocyclic *β*-lactams are found to contain a longer C-2/N amide bond length (1.35–1.38 Å) when compared with normal amide bond length of 1.33 Å; also a shorter C=O bond length (1.21–1.23 Å) is observed when compared with a standard amide bond length of 1.24 Å [68]. The data obtained for compounds **33** and **43** show the *β*-lactam C=O bonds were within the expected range for a monocyclic β-lactam, as were the C-2/N(amide) bond lengths [(1.3706 (15) Å and 1.3724 (19) Å respectively]. The C-2/C-3 bond lengths of 1.5339 (17) Å and 1.534 (2) Å were in the expected range of 1.52–1.55 Å for a β-lactam, while the N/C-4 bond lengths (1.4851 (15) Å and 1.4851 (18) Å respectively were also within the expected range of 1.49–1.51 Å [68,69]. (Numbers in parentheses refer to the second crystallographically independent molecule in the asymmetric unit).

### 2.3. Stability Study for β-Lactams ***33*** and ***39***

The β-lactam ring is known to undergo hydrolysis/degradation depending on the substituent type on the ring. For example, degradation of monocyclic β-lactam antibiotic aztreonam increased with elevated humidity, temperature and acidic pH buffer [70]. Ezetimibe, a monocyclic β-lactam drug used to reduce blood cholesterol, undergoes degradation and hydrolysis more rapidly in neutral and basic pH conditions than in acidic pH [71]. The stability of representative compounds **33** and **39** was also determined at pH 4, pH 7.4 (plasma) and pH 9 (intestine) by HPLC. Interestingly, compound **33** bearing a fluoro substituent at C-3 of β-lactam together with a 3-fluoro on ring B is stable at buffered systems pH 4 and pH 7.4 with 76% and 87%, respectively, remaining at 24 h, while 26% remained at pH 9. The electron-withdrawing effect of the 3-fluoro substituent may result in decreasing stability of the β-lactam ring in response to acid hydrolysis. For the 3,3-difluoro compound **39**, 50% remained after 24 h at pH 7.4, 39% at pH 4, while the compound was less stable at pH 9 with 19% remaining at 24 h. Compounds **33** and **39** were further examined under degradation conditions; **33** was found to be stable with 90%, 88%, 60%, 65% and 65% of the compound remaining after 4 h treatment in heat (60 °C), UV light, acidic (0.1 M HCl), alkaline (0.1M NaOH) and oxidative (3% H_2_O_2_) conditions, respectively. The 3,3-difluoro compound **39** was slightly less stable in these conditions, with 82%, 61%, 54%, 60% and 50% of the compound remaining, respectively (see Supplementary Materials, Table S7).

### 2.4. Predicted Physicochemical and ADME Properties

Consideration of physicochemical and pharmacokinetic properties of active molecules is useful in the early stages of drug discovery to determine the potential of compounds for further development. The physicochemical properties and metabolic stability predicted for the panel of 3-fluoro and 3,3-difluoro-β-lactams **26**–**45** were compiled to assess the relevant drug properties of the series (see Supplementary Materials for Tier 1 profiling screen, Tables S1 and S2). Pipeline Pilot Professional [72] was used to calculate the relevant physicochemical and pharmacokinetic properties. The synthesised compounds followed Lipinski and Veber rules with a molecular weight less than 500 Da (within the range of 361–458 Da), ≤10 hydrogen bond acceptors, ≤5 hydrogen bond donors and ≤10 rotatable bonds. The logP for all synthesised compounds was determined to be less than 5 (Supplementary Materials, Table S2) and was in the range 2.68–4.00. The topological polar surface area (TPSA), indicating the ability of the compound to form hydrogen bonds and to permeate cells (indicating good intestinal absorption), was calculated to be in the range 57.23–77.46 Å^2^ and within the acceptable limit of ≤140 Å^2^. All compounds were predicted to have good passive gastrointestinal absorption properties, high blood–brain barrier (BBB) absorption levels and good plasma protein binding properties (>90%) and were not predicted to inhibit CYP2D6. The synthesised compounds are predicted to be un-ionised at physiological pH. The theoretical pKaH values of phenolic compound **32** calculated with Marvin are 9.82 (phenolic OH) and 11.47 (CH-F), while the corresponding values for compounds **33** and **42** are 11.46 (CH-F) and 9.82 (CH-F), respectively. The compounds are predicted to have low aqueous solubility, with the exception of phenolic compound **32** which is predicted to have good aqueous solubility (logSw = −3.8020 mol/L) (see Supplementary Materials, Table S1). These compounds were soluble in EtOH and DMSO for biological evaluation; improved water solubility may be achieved using phosphate esters, as for CA-4 [73]. In addition, the compounds did not signal an alert for pan-assay interference compounds (PAINS) [74]. As the compounds are predicted to demonstrate good drug-like parameters and bioavailability [75], we proceeded with further biochemical studies to examine their mechanism of action.

### 2.5. Biochemical Results

#### 2.5.1. In Vitro Antiproliferative Activity of 3-Fluoro β-Lactams and 3,3-Difluoro β-Lactams in MCF-7 Breast Cancer Cells

**Figure 3:**
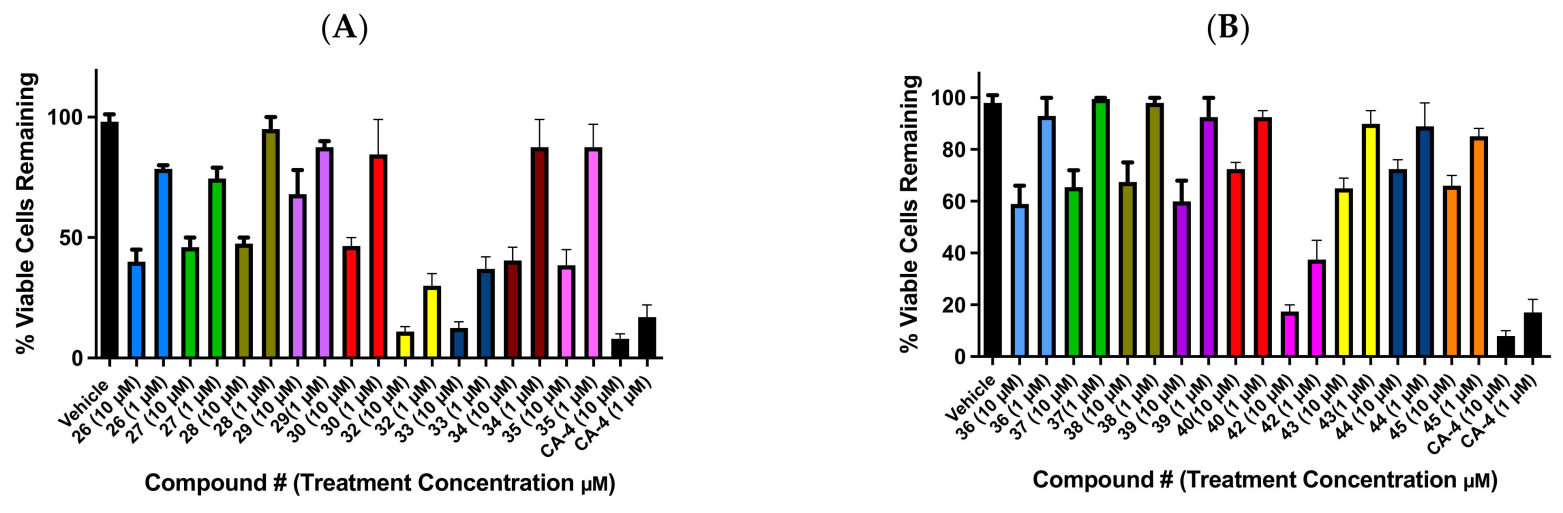
Preliminary cell viability data for 3-fluoro and 3,3-difluoro β-lactam compounds. (**A**) 3-Fluoro β-lactam compounds containing different *para*-substituent ring B **26**–**29**; 3-fluoro β-lactam compounds containing different *meta*-substituent ring B **30**, **32**–**35**; (**B**) 3-difluoro β-lactam compounds containing different *para*-substituent ring B **36**–**39**; 3-difluoro β-lactam compounds containing different *meta*-substituent ring B **40**, **42**–**45** in MCF-7 breast cancer cells. Cell proliferation of MCF-7 cells was determined with an AlamarBlue assay (seeding density 2.5 × 10^5^ cells/mL per well for 96-well plates). Compound concentrations of either 10 μM or 1 μM for 72 h were used to treat the cells with control wells containing vehicle ethanol (1% *v*/*v*) and CA-4 (10 μM and 1 μM). The mean value for three experiments is shown with the ± S.E.M. for three independent experiments.

The antiproliferative potential of the synthesised 3-fluoro β-lactams **26**–**35** and 3,3-difluoro β-lactams **36**–**45** was evaluated in MCF-7 human breast cancer cells using the AlamarBlue assay, with CA-4 as reference compound (Figure 3). These compounds were screened at two concentrations (1 and 10 μM) to identify the most potent compounds for further investigations. The results for the preliminary in vitro antiproliferative screen for 3-fluoro β-lactam compounds (**26**–**29**), which contain different substituents at the *para* position in the B ring, are presented in Figure 3A. Compounds **26** (OCH_3_), **27** (OCH_2_CH_3_) and **28** (SCH_3_) exhibited good anticancer activity with 40, 46 and 47% viable cells remaining at 10 μM but were only weakly active at 1 μM concentration (>70% viable cells of MCF-7). Compound **29** containing the SCH_2_CH_3_ substituent was weakly active with 68–90% cell viability for these two concentrations, much less potent compared to the corresponding derivatives containing OCH_3_, OCH_2_CH_3_ and SCH_3_ substituents. The 3-fluoro β-lactam compounds (**30**, **32**–**35**) having different *meta* substituents on ring B showed more potent cell growth inhibition activity at the higher concentration (10 μM), with 11–46% viable cells remaining after treatment (Figure 3A). Compounds **32** (3-hydroxy) and **33** (3-fluoro) proved to be the most active of the 3-fluoro β-lactam derivatives in this series, with 30 and 37% viable MCF-7 cells at 1 μM.

The antiproliferative effect of 3,3-difluoro β-lactams **36**–**39** containing different *para* substituents on ring B is displayed in Figure 3B. All compounds in this series exhibited much weaker activity with 59–67% viable cells at 10 μM and >90% at 1 μM compared to the corresponding 3-fluoro β-lactam derivatives (**26**–**29**). In a similar trend, 3,3-difluoro compounds **40** and **42**–**45** containing various *meta* substituents on ring B showed poorer antiproliferative activity with >60% cell viability at 10 μM and >80% cell viability at 1 μM compared to their corresponding 3-fluoro β-lactam derivatives (**30**, **32**–**35**) (Figure 3B). The exception in this series was compound **42** containing *meta*-hydroxy substituent on ring B, which elicited potent anticancer effects at both concentrations (1 and 10 μM) with 17% and 37% viable MCF-7 cells remaining, respectively. The results suggested that the introduction of an additional fluorine substituent at C-3 of the β-lactam ring significantly reduced the cell growth inhibition activity for most of the compounds and affects the interaction of the β-lactam with the target tubulin-binding site.

**Figure 4:**
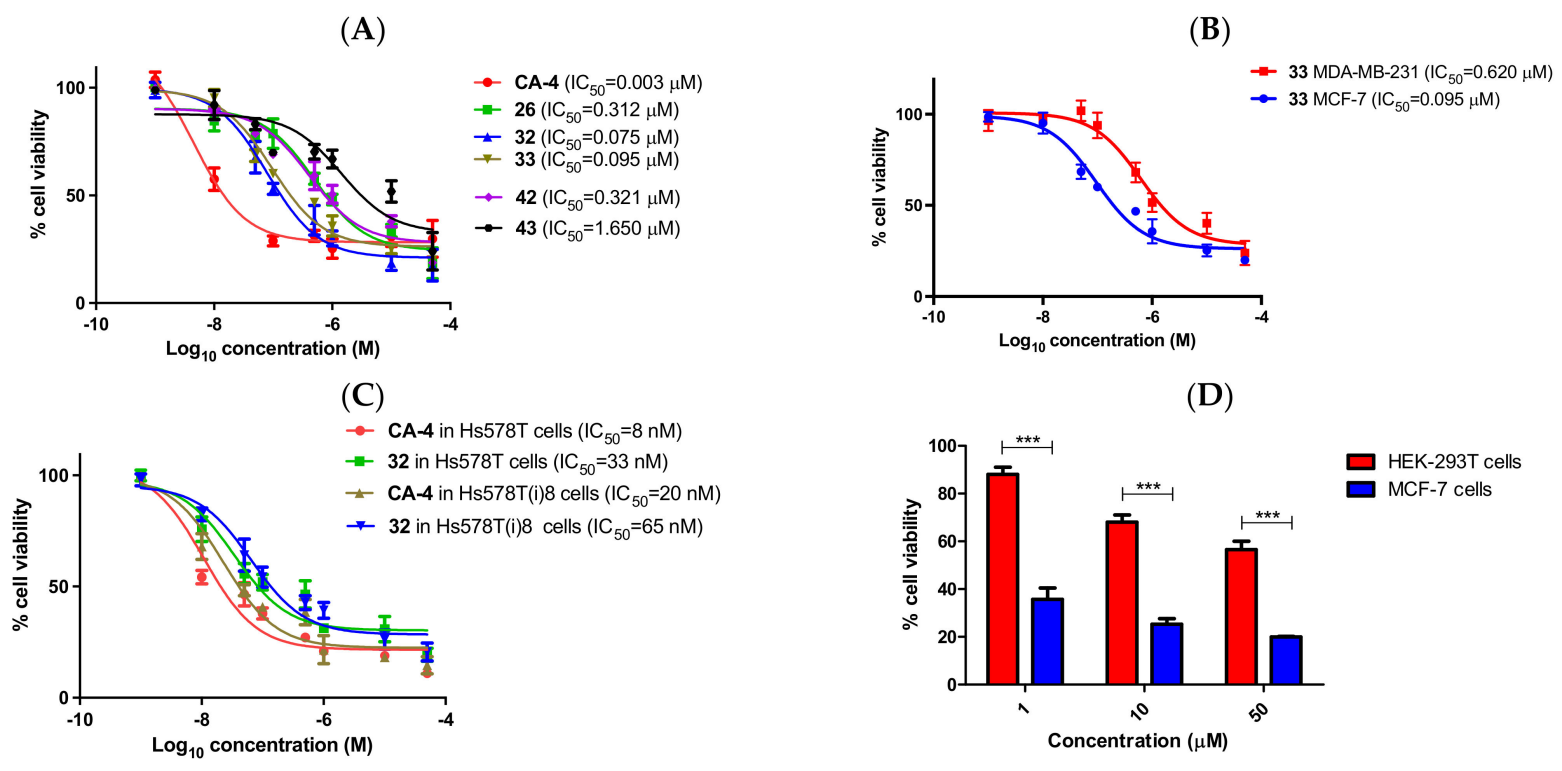
Antiproliferative activity of 3-fluoro β-lactams on MCF-7, MDA-MB-231, Hs578T and Hs578T isogenic subclone Hs578T(i)8 breast cancer cells and non-tumourigenic HEK-293T cells. (**A**) Antiproliferative activity for 3-fluoro β-lactams **26**, **32**, **33**, **42** and **CA-4** in MCF-7 cells. Cells were grown in 96-well plates and treated with indicated β-lactam compounds at 0.01–50 μM for 72 h. Cell viability was expressed as a percentage of vehicle control (ethanol 1% (*v*/*v*))-treated cells. The values represent the mean ± S.E.M. for three independent experiments performed in triplicate. (**B**) Antiproliferative activity of 3-fluoro β-lactam **33** in MCF-7 and MDA-MB-231 cells. (**C**) Antiproliferative activity of 3-fluoro β-lactam **33** in triple-negative breast cancer cell line Hs578T and its isogenic subclone Hs578T(i)8. (**D**) Effect of compound **33** on viability of MCF-7 and non-tumourigenic HEK-293T cells. Cells were grown in 96-well plates and treated with compound **33** at 1, 10 and 50 μM for 72 h. Cell viability was expressed as a percentage of vehicle control (ethanol 1% (*v*/*v*))-treated cells and was measured by AlamarBlue assay (average of three independent experiments). Two-way ANOVA (Bonferroni post-test) was used to test for statistical significance (***, *p* < 0.05).

Figure 3 fluoro and 3,3-difluoro β-lactams, five of the more active compounds (**26**, **32**, **33**, **42** and **43**) were selected for the determination of IC_50_ values against the MCF-7 cell line (Figure 4A and Supplementary Materials Table S4). 3-Fluoro β-lactam **33** exhibited notable antiproliferative activity against the ER-positive MCF-7 breast cancer cells with an IC_50_ value of 0.095 μM (Figure 4A). The positive control CA-4 gave an IC_50_ value of 0.0035 μM in the MCF-7 cell line, which is in good agreement with the reported values for CA-4 in the MCF-7 human breast cancer cell lines [76]. Removal of the ring B 3-fluoro substituent as in compound **26** resulted in a reduction in potency with IC_50_ = 0.312 μM. The 3-fluoro-β-lactam (ring B 3-hydroxy) **32** demonstrated significant potency (IC_50_ = 0.075 μM) compared to its corresponding 3,3-difluoro compound **42** which demonstrated a 4-fold reduction in potency (IC_50_ = 0.321 μM), suggesting that the hydroxyl group contributes to antiproliferative activity but 3,3-difluoro substitution negatively impacts the activity. This effect was also demonstrated in the reduction in activity observed for compound **43** (IC_50_ = 1.65 μM). It is interesting to see that the phenolic compound **32** with lower logP value (2.68) was more potent (IC_50_ value = 0.075 μM) than the corresponding ring B 3-fluoro compound **33** (logP 3.10, IC_50_ value = 0.095 μM); a similar trend was observed for the 3,3-difluoro compounds **42** (logP 2.85, IC_50_ value = 0.321 μM) and **43** (logP 3.29, IC_50_ value =1.65 μM), suggesting that the interaction of the phenolic group at the colchicine-binding site is more favourable than the fluorine for optimal tubulin effect. The introduction of the fluorine substituent at C-3 of the β-lactam ring resulted in antiproliferative activities for compounds **32** and **33** at nanomolar concentrations in MCF-7 breast cancer cells similar in potency to our previously reported 3-chloro, 3-vinyl and 3-methylazetidin-2-ones in MCF-7 breast cancer cells [32,33,57], thus indicating the importance of these substituents for the antiproliferative and antimitotic activity of these compounds.

The potent 3-fluoro-β-lactam compound **33** synthesised was also evaluated in the triple-negative MDA-MB-231 cell line which lacks expression of the estrogen and progesterone receptors (ER/PR) and the HER2 receptor. An IC_50_ value of 0.620 μM was obtained in MDA-MB-231 cells (with incubation time of 72 h), which was not as potent as that observed in the MCF-7 cells (IC_50_ = 0.095 μM) (Figure 4B). The positive control CA-4 gave an IC_50_ value of 0.043 μM in the MDA-MB-231 cell line, which is in agreement with the reported values for CA-4 in the MDA-MB-231 human breast cancer cell line [77,78]. Compound **32** was also evaluated in the triple-negative breast cancer cell line Hs578T and its isogenic subclone Hs578T(i)8 (Figure 4C). Hs578Ts(i)8 cells have 2.5-fold more migratory capacity and 3-fold more invasive capacity through the extracellular matrix than the parental cell line (Hs578T) and have 30% more CD44+/CD24-/low cells and in vivo proliferation [79]. Compound **32** demonstrated potent antiproliferative activity in Hs578T cells (IC_50_ 0.033 ± 0.005 μM) and Hs578Ts(i)8 cells (IC_50_ = 0.065 ± 0.003 μM) and compares well with CA-4 (IC_50_ = 0.008 μM in Hs578T and 0.020 μM in Hs578Ts(i)8 cells). These results demonstrate the potential application of this compound as an anticancer agent in the inhibition of tumour invasion and angiogenesis, which are recognised features of tumour growth and metastasis in aggressive breast cancers. Additional evaluation of the compounds **26**–**45** in the colon cancer cell line SW-480 indicated moderate activity, with compounds **33**, **42**, **26** and **29** being the most potent with 62%, 56%, 58% and 60% cells remaining at 10 μM concentration (see Supplementary Materials, Figure S11).

The cytotoxicity of the potent β-lactam **33** was investigated in the non-tumourigenic HEK-293T cell line (normal human embryonic kidney) at 72 h. As shown in Figure 4D, the cell viability of HEK-293T cells was notably higher than that observed for MCF-7 cells at 50, 10 and 1 μM concentrations of compound **33**. For example, the cell viability of HEK cells at 1 μM was 88% in contrast to the cell viability of 36% for MCF-7 cells at 1 μM. The IC_50_ value for compound **33** was greater than 50 μM in HEK-293T cells (0.095 μM in MCF-7 cells), demonstrating its selectivity towards cancer cells and the lack of toxicity in non-cancerous cells.

#### 2.5.2. NCI 60 Cell Line Screening for β-Lactam Compounds **33**, **37** and **43**

The antiproliferative effects of selected compounds **33**, **37** and **43** were initially evaluated in the NCI 60 cell line screen at 10 μM concentration [80] (see Supplementary Materials Table S3). Compounds **33**, **37** and **43** showed mean growth inhibition of 73.34%, 58.95% and 56.43% at 10 μM over the 60 cell lines tested. Compound **33** demonstrated broad-spectrum activity against the nine panels of cell lines tested (leukaemia, CNS, melanoma, ovarian, renal, non-small-cell lung, colon, breast and prostate cancers). The most potent growth inhibition for all compounds **33**, **37** and **43** was observed in the leukaemia panel with a mean growth inhibition of 90.6%, 91.4% and 86.3%, respectively. The activity in MCF-7 confirmed our results, with 87.2%, 82.8% and 84.9% growth inhibition for compounds **33**, **37** and **43,** respectively.

Compound **33** was next selected for evaluation in the NCI 60 cell line five-dose screen. The GI_50_ (50% growth inhibition), TGI (total growth inhibition) and LC_50_ (50% lethal concentration) were determined in the NCI panel of 60 cell lines, using the sulphorhodamine B (SRB) protein assay (Table 5). The GI_50_ value provides the growth inhibition of the selected compound, while the cytotoxic effect is evaluated in the LC_50_ value. Compound **33** showed good potency in all leukaemia, breast cancer, ovarian, colon and prostate cell lines and excellent antiproliferative activity at submicromolar concentrations in all cell lines except for melanoma cell lines UACC-257 and CNS SNB-75 (Table 5 and Supplementary Materials Table S3). The GI_50_ values displayed by compound **33** for breast cancer cell lines MCF-7 (0.0364 μM) and MDA-MB-231 (0.355 μM) confirmed our in-house values for MCF-7 (0.095 μM) and MDA-MB-231 (0.620 μM). Compound **33** exhibited promising antiproliferative activity in the leukemia cell line K-562 (GI_50_ = 0.038 μM), melanoma cell line M14 (GI_50_ = 0.025 μM), non-small-cell lung cancer cell line NCI-H552 (GI_50_ = 0.038 μM), renal cancer cell line RXF393 (GI_50_ = 0.039 μM), colon cancer cell line HCT-116 (GI_50_ = 0.0325 μM) and the adenocarcinoma-derived adriamycin-resistant ovarian tumour cell line NCI/ADR-RES (GI_50_ = 0.042 μM). The compound also displayed good antiproliferative activity in the glioblastoma cell lines U251 (GI_50_ = 0.0236 μM) and SF-539 (GI_50_ = 0.0280 μM). Glioblastoma is an aggressive and fast-growing brain tumour with a median survival time of 9–16 months from diagnosis, and these tumours quickly evolve resistance to temozolomide chemotherapy, which is the only FDA-approved treatment [81]. Additionally, the cytotoxicity of compound **33** (determined as the LC_50_ value) was greater than 100 μM in all cell lines (except COLO 205 (LC_50_ 8.23 μM) and SK-MEL-5 (LC_50_ 91.3 μM)), indicating minimal cell death and toxicity. The antiproliferative activity displayed by compound **33** (mean GI_50_ value 0.2238 μM, mean TGI value 52.48 μM and mean LC_50_ value 95.49 μM, Table 6) indicated a significant therapeutic window between the concentration required for inhibition of growth of the cancer cells and the concentration of the compound that is lethal to the cells, demonstrating the potential for further development. Further mechanistic and cellular studies were carried out and are described below.

**Table 5:** Antiproliferative activity of compound **33** in NCI 60 cell line screen ^a,b,c,d^.

*Panel*	*Cell Line*	GI_50_ (μM) ^b^	TGI (μM) ^c^	*Panel*	*Cell Line*	GI_50_ (μM) ^b^	TGI (μM) ^c^
***Leukaemia***	CCRF-CEM	0.0471	>100	***Melanoma***	LOX IMVI	0.0904	>100
	HL-60(TB)	0.0805	0.399		MALME-3M	Nd ^d^	>100
	K-562	0.0383	>100		M14	0.0254	Nd ^d^
	MOLT-4	0.146	93.4		MDA-MB-435	0.0291	Nd ^d^
	RPMI-8226	0.055	37.3		SK-MEL-2	0.303	>100
	SR	0.0408	>100		SK-MEL-28	15.8	>100
***NSCLung***	A549/A TCC	0.394	>100		SK-MEL-5	0.0456	0.35
	EKVX	0.203	>100		UACC-257	>100	>100
	HOP-62	0.511	>100		UACC-62	Nd^d^	>100
	HOP-92	0.266	63.9	***Ovarian***	IGROV1	0.0946	>100
	NCI-H226	22.2	>100		OVCAR-3	0.0465	0.492
	NCI-H23	0.273	>100		OVCAR-4	0.966	>100
	NCI-H322M	1.63	>100		OVCAR-5	0.423	>100
	NCI-H460	0.356	91.1		OVCAR-8	0.368	>100
	NCI-H522	0.0383	>100		NCI/ADR-RES	0.0424	>100
***Colon***	COLO 205	0.156	0.503		SK-OV-3	3.45	>100
	HCC-2998	0.406	>100	***Renal***	786–0	0.0462	15.1
	HCT-116	0.0325	>100		A498	0.847	8.08
	HCT-15	0.0664	>100		ACHN	Nd ^d^	Nd ^d^
	HT29	0.134	>100		RXF 393	0.0398	60
	KM12	0.0596	>100		SN12C	0.482	>100
	SW-620	0.0562	>100		TK-10	38.6	>100
***CNS***	SF-268	0.324	>100		UO-31	0.171	>100
	SF-295	0.0535	>100	***Prostate***	PC-3	0.042	>100
	SF-539	0.028	0.217		DU-145	0.269	>100
	SNB-19	0.302	>100	***Breast***	MCF7	0.0364	>100
	SNB-75	>100	>100		MDA-MB-231/ATCC	0.355	>100
	U251	0.0236	>100		HS 578T	0.295	>100
					BT-549	0.0498	33.8
					T-47D	Nd ^d^	>100
					MDA-MB-468	0.106	Nd ^d^

^a^ NCI in vitro human tumour cell screen 5-dose assay for compound **33** (NSC 792959). The compound was evaluated using five different concentrations (100 µM, 10 µM, 1 µM, 0.1 µM and 0.01 µM) over the NCI 60 cell line panel, and incubations were carried out over 48 h exposures to the drug [80]. ^b^ GI_50_ is the molar concentration of the compound causing 50% inhibition of growth of the tumour cells. ^c^ TGI: total growth inhibition; TGI is the mean concentration required to completely inhibit the growth of all cells in the NCI 60 cell line panel. ^d^ Nd: Not determined.

**Table 6:** NCI 60 cell line in vitro primary screening results for compound **33** ^a^.

NCI Ref No.	Compound	Structure	MG-MID GI_50_ (µM) ^b^	MG-MID TGI (µM) ^c^	MG-MID LC_50_ (µM) ^d^
D-613729	**CA-4**	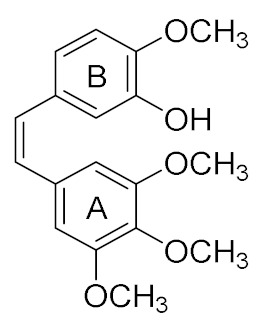	0.099	10.3	85.5
D-792959	**33**	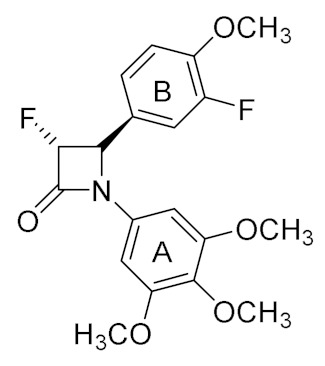	0.223	52.4	95.49

^a^ Data obtained from NCI in vitro human tumour cell screen 5-dose assay for CA-4 and compound **33** (NSC 792959). The GI_50_, TGI and LC_50_ values are determined; they represent the molar drug concentration required to cause 50% growth inhibition, the concentration required to cause total growth inhibition and the concentration that kills 50% of the cells, respectively. The compounds were evaluated using five different concentrations (100 µM, 10 µM, 1 µM, 0.1 µM and 0.01 µM) over the total NCI 60 cell line panel in the assay, and incubations were carried out over 48 h exposures to the drug [80]. ^b^ MG-MID: mean-graph midpoint GI_50_ values. ^c^ MG-MID mean-graph midpoint TGI values. ^d^ MG-MID: mean-graph midpoint LC_50_ values of compounds in the NCI cell panel.

To gain insight into the potential cellular target and molecular mechanism correlating with the cytotoxic effect of **33**, NCI COMPARE analysis was carried out [82]. The NCI five-dose cell growth data of **33** were compared with the NCI databases (Standard and Synthetic). The COMPARE algorithm ranks the compared compounds from the NCI databases (Standard and Synthetic, >55,000 compounds) to the seed compound **33** (NCI ref 792959), using Pearson correlation coefficients for the correlation. A correlation coefficient of greater than 0.8 indicates a strong correlation and suggests that the seed compound may have a mechanism of action similar to that of the highly correlated compounds [83,84]. The comparison with the NCI standard database of clinically used drugs resulted in the top-ranked compounds of macbecin II (0.53, Hsp90 inhibitor) and vincristine (0.5, tubulin inhibitor), with values below the acceptable value for useful prediction of the mechanism of action (see Supplementary Materials Table S5, Figure S12). However, the COMPARE analysis using the NCI Synthetic Database of compounds which have known and unknown mechanisms of action resulted in the identification of 19 compounds with correlations in the range of 0.85–0.75 based on GI_50_ values (see Supplementary Materials Table S6 for details, together with the structures of the compounds identified). Of these, 12 were identified as acting as microtubule-targeting agents with tubulin binding activity, including the highest-ranking compounds which target the colchicine-binding site of tubulin. Compound **33** was designed to target microtubules, so further investigations were carried out to confirm this mechanism of action.

#### 2.5.3. Effect of Compound **33** on the Apoptosis in MCF-7 Cells through Annexin V–FITC/PI Staining

**Figure 5:**
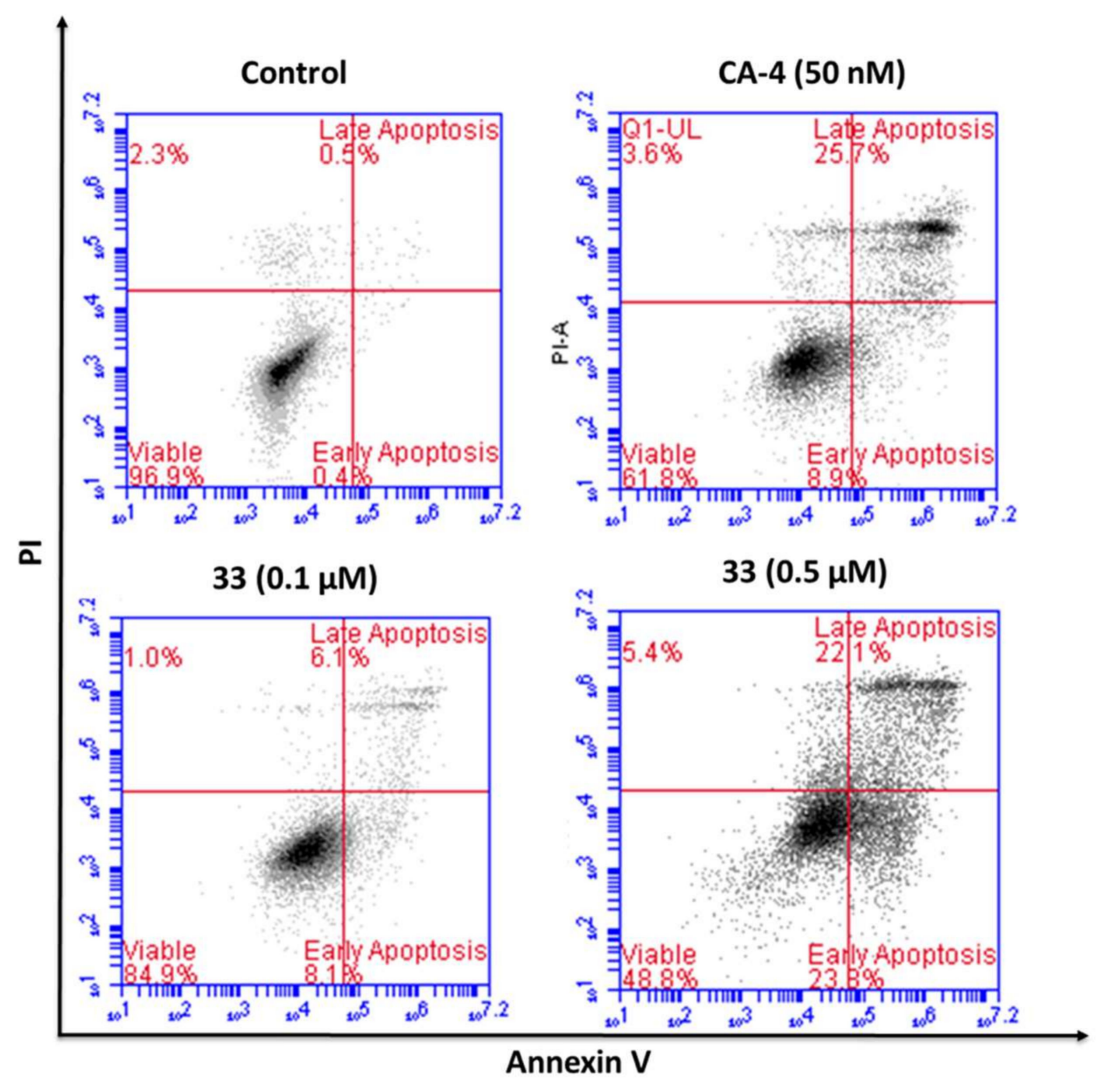
Compound **33** induces apoptosis in MCF-7 breast cancer cells. MCF-7 breast cancer cells were treated with **33** (0.1 and 0.5 µM), CA-4 (50 nM) or vehicle control (0.1% ethanol (*v*/*v*)) for 48 h. The percentage of apoptotic cells was determined by staining with Annexin V–FITC and PI. In each panel, the **lower left** quadrant shows cells that are negative for both PI and Annexin V–FITC, and the **upper left** shows cells that are only negative PI, which are necrotic. The **lower right** quadrant shows Annexin-positive cells which are in the early apoptotic stage, and the **upper right** shows both Annexin- and PI-positive cells, which are in late apoptosis.

Among the cell death pathways, apoptosis is very important to maintain cellular homeostasis. However, upon severe and irreparable cellular stress, all cells, including cancer cells, undergo apoptosis. In this work, the Annexin V–FITC/PI double-staining method was used in assessing the development of apoptosis when MCF-7 cells are exposed to compound **33** (0.1 and 0.5 μM for 48 h). MCF-7 cells were first treated with compound **33** for 48 h followed by staining with Annexin V–fluorescein isothiocyanate (FITC)/propidium iodide (PI). Live cells (Annexin V^−^/PI^−^), early apoptotic cells (Annexin V^+^/PI^−^), late apoptotic cells (Annexin V+/PI+) and necrotic cells (Annexin V^−^/PI^+^) can be differentiated by tie technique following flow cytometric analysis (Figure 5). The positive control CA-4 (at 50 nM concentration) induced 34.6% Annexin V-positive apoptotic cells, and the vehicle control used was 0.1% ethanol (*v*/*v*). Compound **33** was found to induce both early and late apoptosis compared to untreated control cells, and this effect was dose-dependent. β-Lactam **33** induced 14.2% apoptosis (8.1% early apoptotic and 6.1% late apoptotic cells) with 0.1 μM treatment and 45.9% apoptosis (22.1% early apoptotic and 23.8% late apoptotic cells) with 0.5 μM treatment in MCF-7 cells with respect to untreated cells which did not induce any significant apoptotic effect, 0.9% apoptosis (0.4% early apoptotic and 0.5% late apoptotic cells).

#### 2.5.4. Effect of Compound **33** on the Expression of Apoptosis Regulatory Proteins Bcl-2, Bax and Survivin in MCF-7 Cells

Based on the cell viability results for compound **33**, combined with induction of apoptosis, further effects of **33** on the expression of members of the Bcl-2 apoptosis regulatory protein family were next investigated. The Bcl-2 family of apoptosis regulatory proteins includes anti-apoptotic and pro-apoptotic members and is the best-characterised family of proteins involved in the regulation of apoptotic cell death [85]. The pro-apoptotic protein Bax is an important regulator of the intrinsic or mitochondrial apoptosis pathway and triggers the release of caspases, while the anti-apoptotic protein Bcl-2 prevents apoptosis by sequestering caspases or by inhibiting the release of the mitochondrial apoptogenic factors cytochrome c and apoptosis-inducing factor (AIF) into the cytoplasm [86,87].

**Figure 6:**
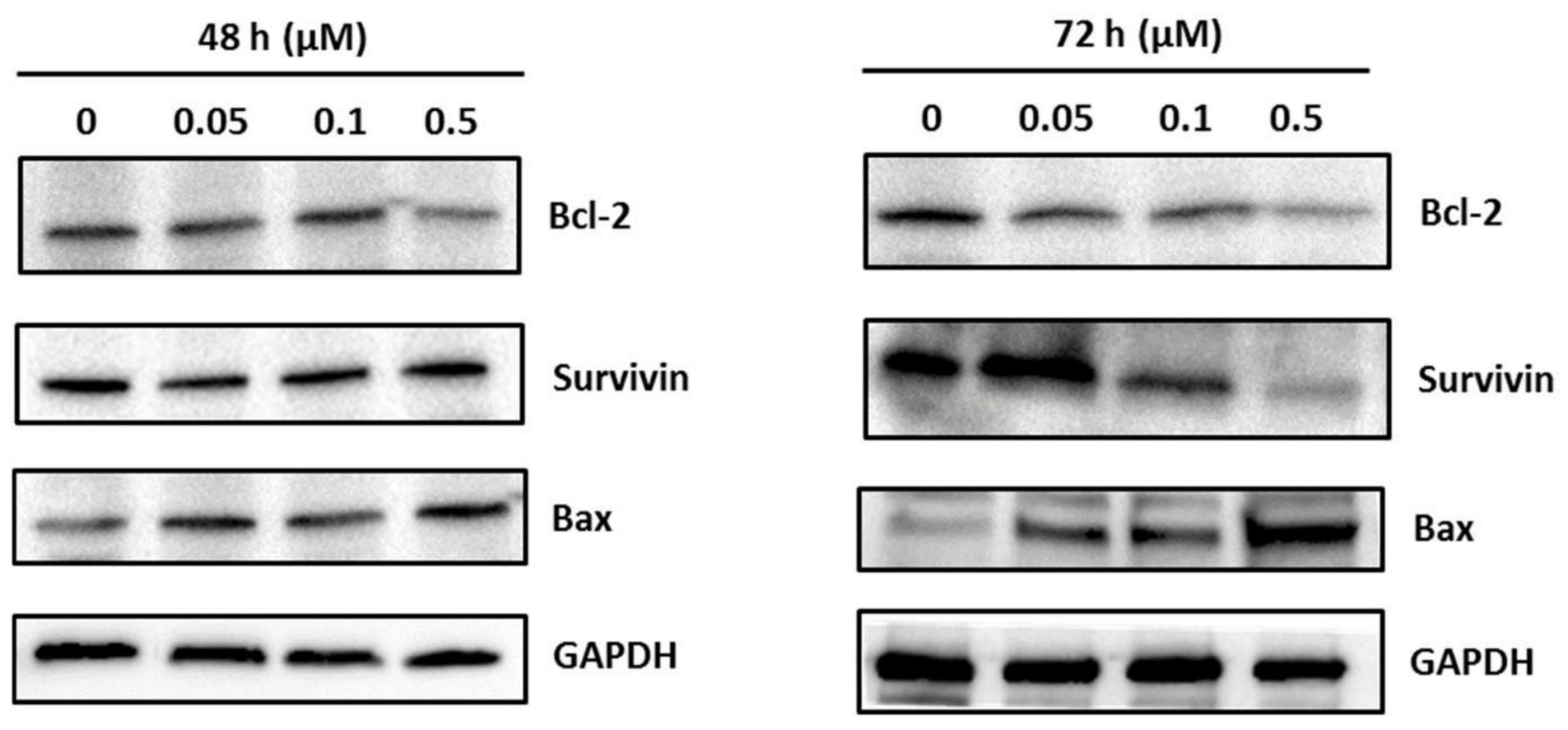
β-Lactam **33** decreases the expression of anti-apoptotic proteins Bcl-2 and survivin and increases the expression of pro-apoptotic protein Bax in MCF-7 cells. MCF-7 cells were treated with vehicle control (ethanol 0.1% *v*/*v*) or **21** at the indicated concentrations (0.05, 0.1 or 0.5 μM) for 48 h (**left**) or 72 h (**right**). Then, the cells were harvested for Western blot analysis to detect the level of the apoptosis-related proteins. Results are indicative of three separate experiments, performed independently. To confirm equal protein loading, each membrane was stripped and re-probed with GAPDH antibody.

The selective Bcl-2 inhibitor venetoclax was developed as a BH3-mimetic that binds to the pro-survival protein Bcl-2 and inhibits its ability to bind Bax or Bak. Venetoclax has been approved for clinical use in the treatment of chronic lymphocytic leukaemia (CLL) [86]. MCF-7 cells were treated with compound **33** (0.05, 0.1 and 0.5 μM) for 48 and 72 h as shown in Figure 6. Compound **33** induced downregulation of Bcl-2 expression with corresponding upregulation of Bax at 48 h in a dose-dependent manner. This effect was further increased at 72 h.

Survivin is a member of the inhibitor of apoptosis (IAP) protein family. It is an essential anti-apoptotic protein marker that is overexpressed in most tumour cells and is associated with a poor clinical outcome. Survivin inhibits caspase activation, which is used as an indicator of apoptosis cascades [88,89,90]. Survivin selective inhibitor molecules have been identified as cancer therapeutics, including survivin–partner protein interaction inhibitors, survivin homodimerisation inhibitors, survivin gene transcription inhibitors and survivin mRNA inhibitors [91]. We examined the effect of a range of concentrations of compound **33** (0.05, 0.1 and 0.5 μM) on the level of survivin expressed. Compound **33** caused a downregulation of the expression of survivin in a dose- and time-dependent manner, as demonstrated by Western blotting (Figure 6), confirming the pro-apoptotic effect of **33** in MCF-7 cancer cells. The upregulation of the pro-apoptotic protein Bax and the downregulation of the anti-apoptotic proteins Bcl-2 and survivin support the pro-apoptotic mechanism of action suggested for compound **33**, also indicated from the Annexin V/PI flow cytometric analysis.

#### 2.5.5. Effect of Compound **33** on Tubulin Polymerisation

**Figure 7:**
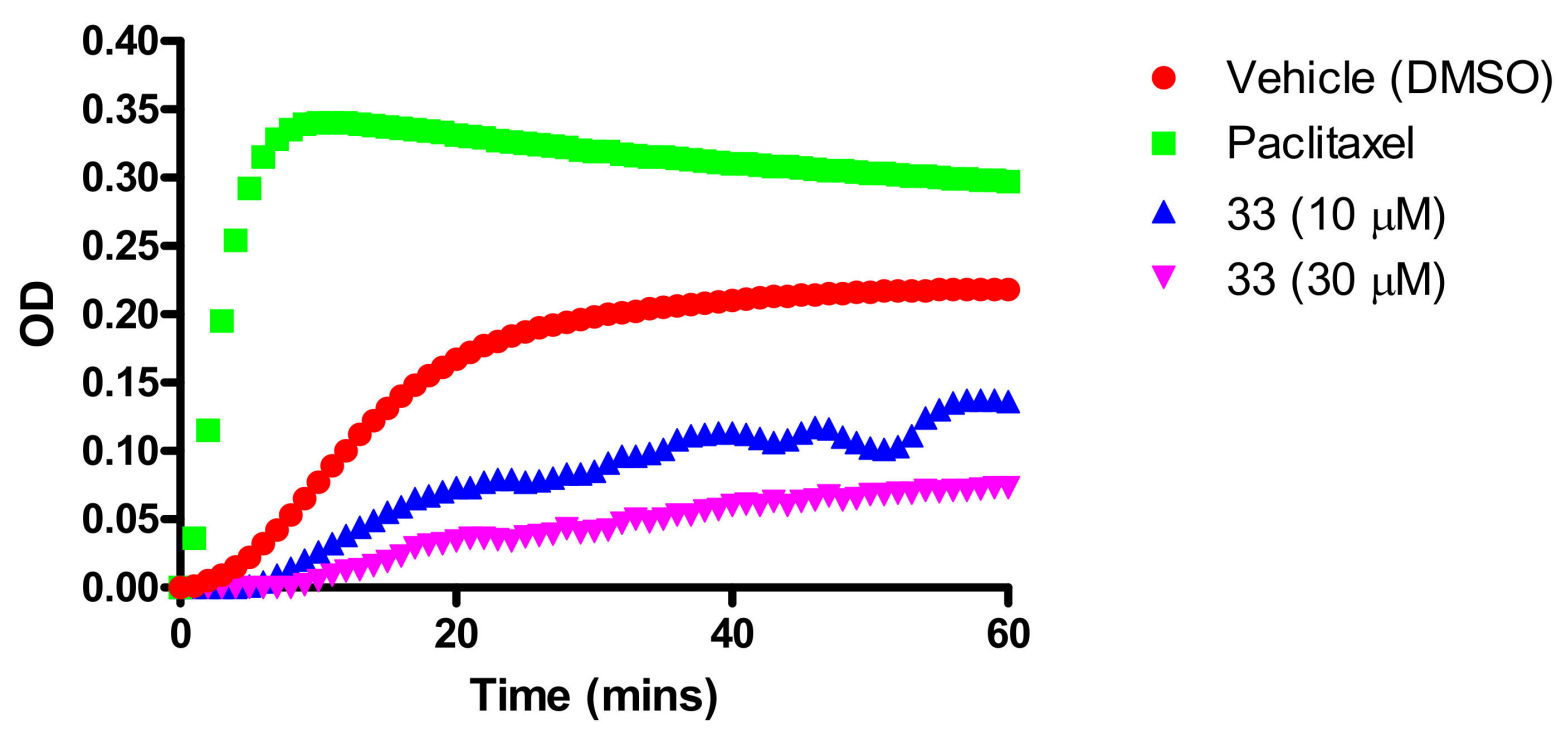
Effect of β-lactam compound **33** (10 and 30 µM) and paclitaxel (10 µM) on in vitro tubulin polymerisation. Purified bovine tubulin and GTP were mixed in a 96-well plate. Compounds were added and the reaction was started by warming the solution from 4 to 37 °C. Ethanol (1% *v*/*v*) was used as a vehicle control. The effect on tubulin assembly was monitored in a Spectramax 340PC spectrophotometer at 340 nm at 30 s intervals for 30 min at 37 °C. The graph shows one representative experiment. Each experiment was performed in duplicate.

The effect of representative 3-fluoro β-lactam CA-4 analogue compound **33**, which exhibited potent anticancer effects in vitro, on the polymerisation of the purified tubulin protein was examined. Tubulin polymerisation was investigated using a turbidimetric assay which determines the light scattering by microtubules (absorbance at 340 nm) that is proportional to the microtubule polymer concentration. Paclitaxel (10 µM), which stabilises the tubulin when compared to the vehicle control (DMSO), was the positive control. Tubulin polymerisation results obtained for **33** showed a 3.5-fold reduction in the V_max_ (maximum rate of reaction) at 10 µM compared to the vehicle with an increase to 4-fold reduction at the higher concentration of 30 µM (Figure 7), whereas in our previous work we demonstrated that CA-4 induced a 6.3-fold reduction at 10 μM [33]. This result indicated that tubulin is the molecular target of the antiproliferative 3-fluoro β-lactam compound **33** as reported previously for related heterocyclic CA-4 analogues [33].

**Figure 8:**
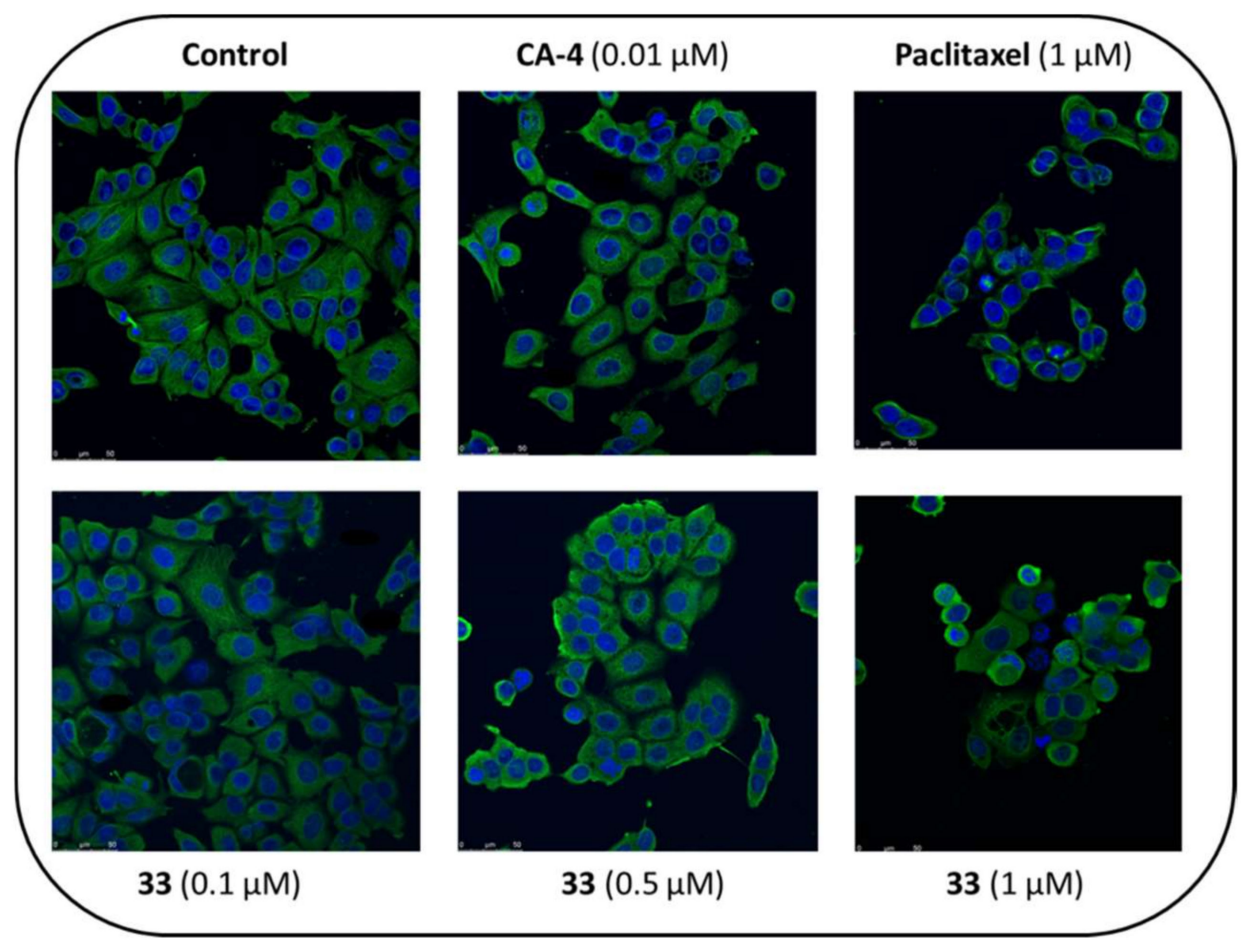
Compound **33** acts as depolymeriser of the microtubule network of MCF-7 breast cancer cells. Cells were treated with vehicle control (0.1% ethanol (*v*/*v*)), CA-4 (0.01 μM), paclitaxel (1 μM) or compound **33** (0.1, 0.5 and 1 μM) for 16 h. Cells were fixed in ice-cold methanol and stained with mouse monoclonal anti-α-tubulin–FITC antibody (clone DM1A) (green) and Alexa Fluor 488 dye and counterstained with DAPI (blue). Images were obtained with Leica SP8 confocal microscopy with Leica Application Suite X software, (LAS V 4.13), Wetzlar, Germany. Representative confocal micrographs of three separate experiments are shown. White scale bar indicates 50 μM.

The tubulin-targeting effect of compound **33** on the microtubule network of MCF-7 cells was further evaluated by immunofluorescence studies to identify the cellular effects that are relevant to its mechanism of action by inhibiting the polymerisation of tubulin. MCF-7 cells displayed a well-organised microtubular network in the control cells (0.1% ethanol (*v*/*v*)) (Figure 8). Clearly, significant stabilisation of microtubules in paclitaxel (1.0 μM)-treated cells was observed, while CA-4-treated cells (0.01 μM, positive control) demonstrated depolymerisation and destabilisation of the cell membrane of microtubules. Treatment of MCF-7 cells with compound **33** (0.1, 0.5 and 1.0 μM) induced cell rounding and distinct abnormalities of the spindle formation as well as a loss of the structured tubulin that impacts the microtubule network structure in a dose-dependent manner. These results confirmed our findings that compound **33** acts by destabilising microtubules, supporting the results from the in vitro tubulin polymerisation assay.

### 2.6. Molecular Modelling Study for Compounds ***32***, ***33***, ***42*** and ***43***

**Figure 9:**
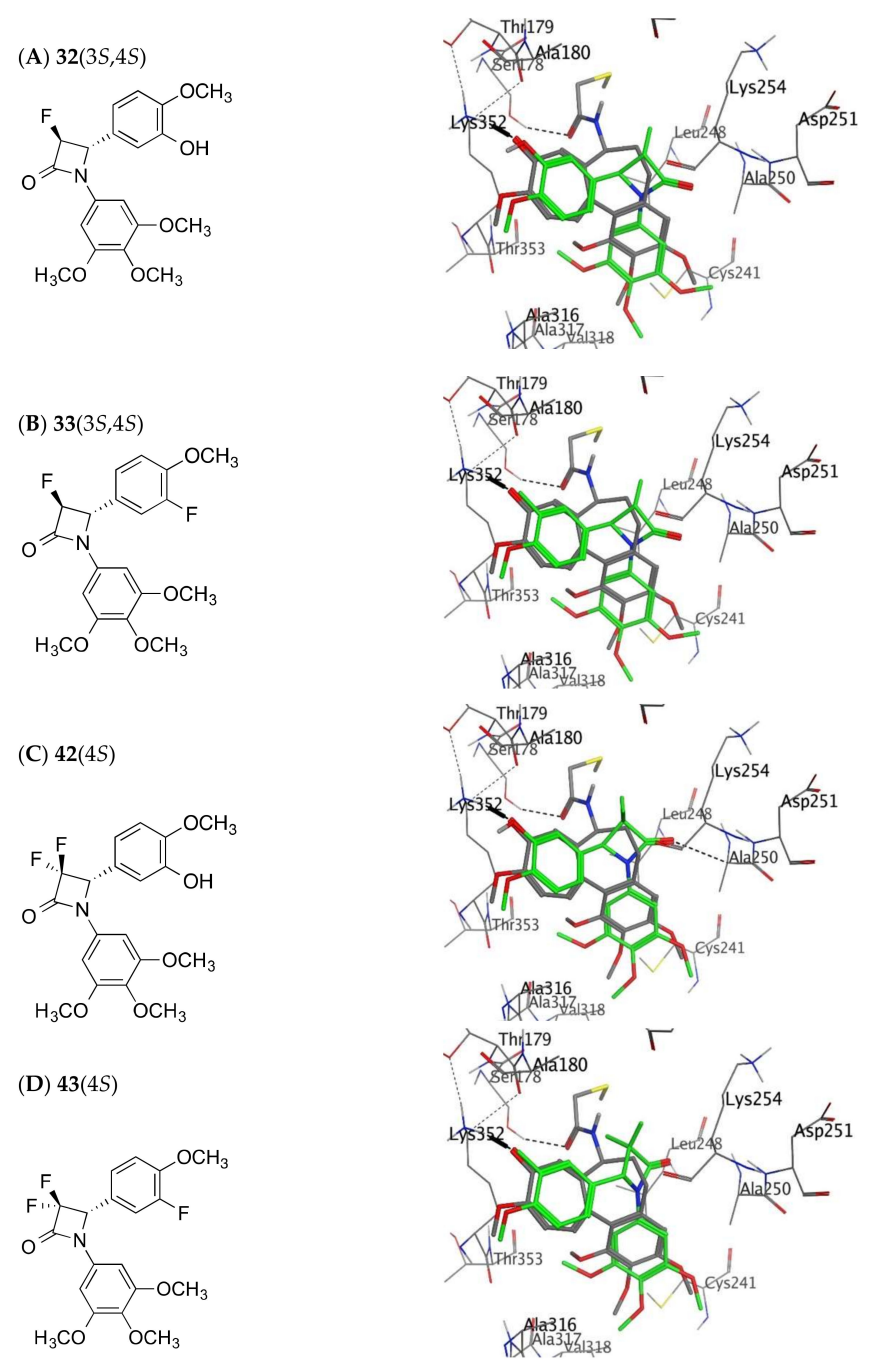
Overlay of the X-ray structure of tubulin co-crystallised with DAMA-colchicine (PDB entry 1SA0) on the best ranked docked poses of the *S* enantiomers of β-lactams: (**A**) **32** (3*S*,4*S*), (**B**) **33** (3*S*,4*S*), (**C**) **42** (4*S*) and (**D**) **43** (4*S*). Ligands are rendered as tubes and amino acids as lines. Tubulin amino acids and DAMA-colchicine are coloured by atom type, the novel β-lactam compounds are coloured with a green backbone. The atoms are coloured by element type; carbon = grey, hydrogen = white, oxygen = red, nitrogen = blue, sulphur = yellow, fluorine = green. Key amino acid residues are labelled and multiple residues are hidden to enable a clearer view.

A series of molecular docking calculations using MOE 2020.09 were undertaken on both enantiomeric pairs of the 3-fluoro-β-lactam compounds **32**, **33**, **42** and **43**, using the tubulin co-crystallised with DAMA-colchicine ligand (X-ray crystal structure PDB entry 1SA0) [19] (Figure 9). It is evident from ^1^H NMR spectroscopy that only the *trans* isomers of the β-lactam compounds were obtained, and therefore the 3*S*/4*S* and 3*R*/4*R* enantiomeric pairs were selected for the modelling analysis. For the compounds with two stereogenic centres, in all cases the *S*,*S* enantiomers were found to be more highly ranked than the corresponding *R*,*R* enantiomeric pair and so will only be discussed in this study. All selected compounds overlaid their B-rings on the C-ring of DAMA-colchicine (resulting in the characteristic hydrogen bond acceptor interactions with Lys352 residue). The 3,4,5-trimethoxyphenyl substituted A-rings occupied the colchicine-binding site, and the 3-fluoro and 3,3-difluoro substituents were located in an open accessible region of the β-tubulin binding site at the monomer interface and did not form interactions with adjacent residues.

The predicted affinity ranking obtained was **32** (3*S*,4*S*), **43** (4*R*), **42** (4*S*), **33** (3*S*,4*S*), **43** (4*S)*, **32** (3*R*,4*R*), **33** (3*R*,4*R*) and **42** (4*R*), ordered from best ranked to worst. The docking scores are provided in Supplementary Materials Table S8. When the conformers were generated with OMEGA [92,93] and docking was run with FRED [94], a similar preference was obtained for *S*,*S* over *R*,*R* enantiomers for compounds **32** and **33**. Docking studies are not always ideal when studying changes in cellular efficacy associated with the C-3 ring B fluoro/hydroxyl substitutions. The best ranked enantiomer, **32** (3*S*,4*S*), was also the most active analogue in MCF-7 cells with IC_50_ of 0.075 μM (Figure 9A). The 3-hydroxy substituent on the 4-phenyl ring (ring B) co-located very well with the C-ring of DAMA-colchicine, with the oxygen positioned to act as a hydrogen bond acceptor with Lys352. The 3,4,5-trimethoxyphenyl ring (ring A) interacted slightly deeper in the binding pocket than that of DAMA-colchicine. The β-lactam carbonyl group potentially formed a hydrogen bond acceptor interaction with a backbone hydrogen atom of Ala250. The 3*S*,4*S* enantiomer of **33** also presented a similar pose in the binding site (Figure 9B). The ring A, 3,4,5-trimethoxyphenyl groups of the compounds illustrated made favourable van der Waals contacts in the lower sub-pocket defined by residues Val β318 and Cys β241. Figure 9B shows the ring B 3-fluoro substituent of compound **33** occupying the same location as the phenolic hydroxyl group in the **32**, acting as a hydrogen bond acceptor with Lys352 which could play a role in stabilisation of protein–drug conformation but which is weaker than the hydrogen bond formed between Lys352 and the hydroxyl group of **32**, which may explain the decrease in cellular efficacy of this compound.

Due to the small volume of the 3,3-difluoro substituents at position 3 of the β-lactam ring, different orientations of these groups did not affect binding, which is evident from the near equal docking scores given to the 3,3-difluoro *R* and *S* enantiomers of **42** and **43**. However, the in vitro antiproliferative activity of the most potent 3-fluoro-β-lactams **32** and **33** of the series is significantly superior when compared with the corresponding 3,3-difluoro-β-lactams **42** and **43** compounds, despite having similar docking scores. Both of the enantiomers of **42** and **43** orientated the 3,4,5-trimethoxyphenyl ring A to overlap with the C-ring of DAMA-colchicine and achieve overlap of the 3,4,5-trimethoxyphenyl groups of colchicine (Figure 9C,D). It is possible that both sets of compounds (monofluoro **32** and **33** and difluoro **42** and **43**) may have slightly different target protein profiles or physicochemical properties such as solubility and cellular permeability. The best ranked binding pose of each compound examined in the study is illustrated in Figure 9, showing the shared binding mode for the selected analogues studied.

## 3. Experimental Section

### 3.1. Chemistry

Melting points (uncorrected) were measured on a Gallenkamp apparatus. Infrared (IR) spectra were recorded on a Perkin Elmer FT-IR Paragon 1000 spectrometer. ^1^H, ^19^F and ^13^C nuclear magnetic resonance spectra (NMR) were recorded on a Bruker DPX 400 spectrometer (400.13 MHz, ^1^H; 100.61 MHz, ^13^C; 376 MHz, ^19^F) in CDCl_3_ (internal standard tetramethylsilane (TMS)) at 27 °C. ^1^H-NMR spectra were assigned relative to the TMS peak at 0.00 ppm and ^13^C-NMR spectra were assigned relative to the middle CDCl_3_ peak at 77.0 ppm. Electrospray ionisation mass spectrometry (ESI-MS) was performed using a liquid chromatography time-of-flight mass spectrometer (Micromass LCT, Waters Ltd., Manchester, UK) in a positive ion mode. The samples were introduced to the ion source by an LC system (Waters Alliance 2795, Waters Corporation, Milford, MA, USA) in acetonitrile:water (60:40 %*v*/*v*) at 200 µL/min, with capillary voltage at 3 kV and sample cone (de-clustering) voltage at 40 V. For exact mass determination, the instrument was externally calibrated for the mass range *m*/*z* 100 to *m*/*z* 1000 with a lock (reference) mass (*m*/*z* 556.2771). TLC was run using Merck Silica gel 60 TLC aluminium sheets with fluorescent indicator visualising with UV at 254 nm; silica gel 60 (230–400 mesh) was used for flash chromatography. Analytical high-performance liquid chromatography (HPLC) was performed using Waters 2487 Dual Wavelength Absorbance detector, Waters 1525 binary HPLC pump and Waters 717 plus Autosampler with Varian Pursuit XRs C18 reverse phase 150 × 4.6 mm chromatography column and UV detection at 254 nm. The mobile phase was acetonitrile (70%):water (30%) over 10 min with a flow rate of 1 mL/min. All synthesised products were homogeneous on TLC when isolated; the purity of the biologically tested compounds was confirmed by HPLC (≥95%). Microwave experiments were carried out using a Biotage Discover SP4 and CEM microwave synthesisers using standard power setting (maximum power 300 watts) unless otherwise stated.

#### 3.1.1. General Method I: Preparation of Imines **16**–**21** and **23**–**25**

The appropriately substituted benzaldehyde (10 mmol) and 3,4,5-trimethoxyaniline (10 mmol) were reacted together at reflux in ethanol (40 mL) for 5 h. Following evaporation of the reaction mixture in vacuo, the product was recrystallised from ethanol. Imines **16**–**21** and **23**–**25** were prepared and characterized as described previously [33,34].

#### 3.1.2. [3-(Tert-butyldimethylsilanyloxy)-4-methoxybenzylidene](3,4,5-trimethoxyphenyl)amine **22**

To a stirred solution of imine **22** (5 mmol) and *tert*-butyldimethylsilyl chloride (6 mmol) in anhydrous CH_2_Cl_2_ (40 mL) under nitrogen was added DBU (8 mmol). After 2–4 h, when the reaction was complete as demonstrated by TLC (eluent, 1:1 hexane/ethyl acetate), the reaction mixture was diluted with CH_2_Cl_2_ (50 mL); washed with water (100 mL), 0.1 M HCl (50 mL) and NaHCO_3_ (50 mL, satd.); and dried (NaSO_4_). Removal of the solvent yielded the imine **22** as an amber oil (yield 52%), purity (HPLC): 100%. Imine **22** was characterised as described previously by us [31,57].

#### 3.1.3. General Method II: Preparation of β-Lactams **26**–**31**, **33**–**41** and **43**–**45**

Activated zinc powder (9 mmol) with trimethylchlorosilane (7 mmol) in anhydrous benzene (4 mL) was heated for 15 min at 40 °C and then at 100 °C for 2 min in a microwave reactor. The mixture was then cooled and the selected imine (2 mmol) and ethyl bromoacetate (5 mmol) were added. The mixture was heated in the microwave reactor for 30 min at 100 °C. The reaction mixture was filtered through Celite; diluted with DCM (30 mL); and washed with ammonium chloride solution (20 mL, satd.) and ammonium hydroxide (20 mL, 25%), HCl (10%, 40 mL) and water (40 mL). The organic phase was dried (anhydrous Na_2_SO_4_) and the solvent was evaporated in vacuo. Isolation of the crude product was achieved by flash column chromatography over silica gel (eluent: hexane:ethyl acetate gradient).

#### 3.1.4. 3-Fluoro-4-(4-methoxyphenyl)-1-(3,4,5-trimethoxyphenyl)azetidin-2-one (**26**)

Compound **26** was obtained from imine **16** and ethyl bromofluoroacetate following the General Method II to afford the product as an oil; yield: 12%, purity (HPLC): 98%. IR ν_max_ (ATR): 1760.0 (C=O) cm^−1^. ^1^H NMR (400 MHz, CDCl_3_): δ ppm 3.68 (s, 3 H, OCH_3_), 3.74 (s, 9 H, OCH_3_), 5.00 (dd, *J* = 1.65 Hz, 1 H, H3), 5.20 (dd, *J* = 10.88, 1.26 Hz, 1 H, H4), 6.51 (s, 2 H, ArH), 6.91 (d, *J* = 8.71 Hz, 2 H, ArH), 7.25 (d, *J* = 8.71 Hz, 2 H, ArH). ^13^C NMR (100 MHz, CDCl_3_): δ ppm 55.34, 56.01, 60.89, 63.67, 95.48, 96.73, 114.77, 127.61, 132.63, 135.70, 153.49, 160.36, 174.36 (C_2,_ C=O). HRMS: calculated for C_19_H_21_FNO_5_ [M + H]^+^ 362.1404; found 362.1409.

#### 3.1.5. 4-(4-Ethoxyphenyl)-3-fluoro-1-(3,4,5-trimethoxyphenyl)azetidin-2-one (**27**)

Preparation of compound **27** following the General Method II from imine **17** and ethyl bromofluoroacetate afforded the product as an oil; yield: 12%, purity (HPLC): 98%. IR ν_max_ (ATR): 1758.3 (C=O) cm^−1^. ^1^H NMR (400 MHz, CDCl_3_): δ ppm 1.58 (t, *J* = 12.23 Hz, 3 H, OCH_2_CH_3_), 3.61 (s, 3 H, OCH_3_), 3.80 (s, 6 H, OCH_3_), 3.74 (m, 2 H, CH_2_CH_3_), 5.30 (d, *J* = 1.23 Hz, 1 H, H3), 5.55 (d, *J* = 10.28, 1.124 Hz, 1 H, H4), 6.57 (s, 2 H, ArH), 6.90 (d, *J* = 8.70 Hz, 2 H, ArH), 7.23 (d, *J* = 8.75 Hz, 2 H, ArH). ^13^C NMR (100 MHz, CDCl_3_): δ ppm 14.22, 55.81, 56.20, 60.8, 62.27, 95.52, 97.53, 114.14, 128.18, 133.73, 135.43, 136.09, 150.98, 160.25, 172.66 (C_2,_ C=O). HRMS: calculated for C_20_H_23_FNO_5_ [M + H]^+^ 376.1560; found 376.1555.

#### 3.1.6. 3-Fluoro-4-(4-(methylthio)phenyl)-1-(3,4,5-trimethoxyphenyl)azetidin-2-one (**28**)

Preparation of compound **28** following the General Method II from imine **18** and ethyl bromofluoroacetate afforded the product as a brown oil; yield: 11%, purity (HPLC): 100%. IR ν_max_ (ATR): 1752.1 (C=O) cm^−1^. ^1^H NMR (400 MHz, CDCl_3_): δ ppm 2.47 (s, 3 H, SCH_3_), 3.69 (s, 6 H, OCH_3_), 3.75 (s, 3 H, OCH_3_), 5.00 (dd, *J* = 1.21 Hz, 1 H, H3), 5.33 (dd, *J* = 10.71, 1.66 Hz, 1 H, H4), 6.51 (s, 2 H, ArH), 7.23–7.25 (m, 4 H, ArH). ^13^C NMR (100 MHz, CDCl_3_): δ ppm 15.57, 55.85, 58.93, 60.94, 62.05, 91.66, 126.67, 127.41, 134.59, 138.53, 142.57, 153.38, 167.81 (C_2,_ C=O). HRMS: calculated for C_19_H_21_FNO_4_S [M + H]^+^ 378.1175; found 378.1185.

#### 3.1.7. 4-(4-(Ethylthio)phenyl)-3-fluoro-1-(3,4,5-trimethoxyphenyl)azetidin-2-one (**29**)

Preparation of compound **29** following the General Method II from imine **19** and ethyl bromofluoroacetate afforded the product as an oil; yield: 12%, purity (HPLC): 98%. IR ν_max_ (ATR): 1762.4 (C=O) cm^−1^. ^1^H NMR (400 MHz, CDCl_3_): δ ppm 1.29 (t, *J* = 7.26, 3 H, SCH_2_CH_3_), 2.94 (m, 2 H, SCH_2_CH_3_), 3.67 (s, 3 H, OCH_3_), 3.74 (s, 6 H, OCH_3_), 5.00 (dd, *J* = 12.44 Hz, 1 H, H3), 5.33 (s, 1 H, H4), 6.49 (s, 2 H, ArH), 7.19–7.26 (m, 2 H, ArH), 7.29 (s, 2 H, ArH). ^13^C NMR (100 MHz, CDCl_3_): δ ppm 14.05, 27.5, 56.15, 60.24, 65.43, 100.12, 106.36, 127.27, 128.19, 130.86, 131.41, 134.92, 139.52, 154.27, 169.38 (C_2,_ C=O). HRMS: calculated for C_20_H_23_FNO_4_S [M + H]^+^ 392.1332; found 392.1353.

#### 3.1.8. 3-Fluoro-4-(4-methoxy-3-methylphenyl)-1-(3,4,5-trimethoxyphenyl)azetidin-2-one (**30**)

Preparation of compound **30** following the General Method II from imine **20** and ethyl bromofluoroacetate afforded the product as a yellow oil; yield: 58%, purity (HPLC): 96%. IR ν_max_ (ATR): 1736.1 (C=O) cm^−1^. ^1^H NMR (400 MHz, CDCl_3_): δ ppm 2.29 (s, 3 H, CH_3_), 3.66 (s, 3 H, OCH_3_), 3.68 (s, 3 H, OCH_3_) 3.70 (s, 6 H, 2xOCH_3_), 5.01 (dd, *J* = 2.90 Hz, 1 H, H3), 5.13 (dd, *J* = 10.14, 2.90 Hz, 1 H, H4), 5.80 (s, 2 H, ArH), 5.86 (s, 1 H, ArH), 6.18 (s, 1 H, ArH), 6.53 (s, 1 H, ArH). ^13^C NMR (100 MHz, CDCl_3_): δ ppm 14.23, 55.20, 56.95, 65.32, 100.58, 101.22, 112.34, 122.17, 125.26, 129.72, 134.21, 135.63, 137.52, 152.96, 156.24, 170.30 (C_2,_ C=O). HRMS: calculated for C_20_H_23_FNO_5_ [M + H]^+^ 376.1560; found 376.1567.

#### 3.1.9. 4-(3-(Tert-butyldimethylsilyl)-4-methoxyphenyl)-3-fluoro-1-(3,4,5-trimethoxyphenyl)azetidin-2-one (**31**)

Preparation of compound **31** following the General Method II using imine **21** and ethyl bromofluoroacetate afforded the product as an orange oil; yield: 18%, purity (HPLC): 98%. IR ν_max_ (ATR): 1745.1(C=O) cm^−1^. ^1^H NMR (400 MHz, CDCl_3_): δ ppm: 0.09 (s, 3H, SiCH_3_), 0.10 (s, 3H, SiCH), 0.97 (s, 9H, *t*-butyl), 3.73 (s, 3H), 3.74 (s, 6H), 3.81 (s, 3H), 4.85 (apparent dd, 1H, ^3^*J*_F-H_ = 25.3 Hz, ^3^*J* = 2.1 Hz, H_4_), 5.14 (apparent dd, 1H, ^2^*J*_F-H_ = 47.4 Hz, ^3^*J* = 2.1 Hz, H_3_), 5.83 (s, 2H), 6.84 (d, 1H, ^3^*J* = 7.8 Hz, Ar-H), 6.86 (d, 1H, ^4^*J* = 1.7 Hz, Ar-H), 6.95 (dd, 1H, ^4^*J*′ = 2.7 Hz, ^3^*J* = 7.8 Hz, Ar-H) ^13^C NMR (100 MHz, CDCl_3_): δ ppm −1.5 (Si-CH_3_), 28.14 (CH_3_), 31.42 (Si-C-CH_3_), 55.6, 55.9, 58.9 (C_3_, *J*_F-C_ = 21 Hz), 61.1, 61.5, 62.0, 69.0, 91.7 (*J*_C-F_ = 91 Hz, C_4_), 95.0, 112.2, 119.7, 120.3, 130.1, 130.7, 145.3, 150.8, 153.8, 167.9 (d, *J*_C-F_ = 25.6 Hz, C_2_) ^19^F NMR (376 MHz CDCl_3_): δ −203.7. HRMS: calculated for C_25_H_35_FNO_5_Si [M + H]^+^ 476.2268; found 476.2276.

#### 3.1.10. 3-Fluoro-4-(3-fluoro-4-methoxyphenyl)-1-(3,4,5-trimethoxyphenyl)azetidin-2-one (**33**)

Preparation of compound **33** following the General Method II from imine **23** and ethyl bromofluoroacetate afforded the product as a yellow oil; yield: 25%, purity (HPLC): 94%. IR ν_max_ (ATR): 1760.7 (C=O) cm^−1^. ^1^H NMR (400 MHz, CDCl_3_): δ 3.89 (s, 3 H, OCH_3_), 3.70 (s, 6 H, 2xOCH_3_), 3.76 (s, 3 H, OCH_3_), 5.18 (dd, *J* = 1.66 Hz, 1 H, H3), 5.31 (dd, *J* = 11.12, 1.24 Hz, 1 H, H4), 6.50 (s, 2 H, ArH), 6.96–7.08 (m, 3 H, ArH). ^13^C NMR (100 MHz, CDCl_3_): δ ppm 55.85, 58.93, 60.94, 62.05, 90.42, 91.66, 126.67, 127.41, 130.66, 134.59, 138.53, 138.83, 142.57, 151.32, 153.77, 167.57 (C_2,_ C=O). ^19^F NMR (376 MHz, CDCl_3_): δ ppm: −132.65 (ArF), −188.43 (C-F, β-lactam). HRMS: calculated for C_19_H_20_F_2_NO_5_ [M + H]^+^ 380.1310; found 380.1316.

#### 3.1.11. 4-(3-Chloro-4-methoxyphenyl)-3-fluoro-1-(3,4,5-trimethoxyphenyl)azetidin-2-one (**34**)

Preparation of compound **34** following the General Method II from imine **24** and ethyl bromofluoroacetate afforded the product as a brown oil; yield: 7%, purity (HPLC): 95%. IR ν_max_ (ATR): 1762.0 (C=O) cm^−1^. ^1^H NMR (400 MHz, CDCl_3_): δ ppm 3.68 (s, 6 H, 2xOCH_3_), 3.70 (s, 3 H, OCH_3_), 3.72 (s, 3 H, OCH_3_), 4.96 (dd, *J* = 9.20, 2.70 Hz, 1 H, H3), 5.30 (dd, *J* = 2.07 Hz, 1 H, H4), 6.48 (s, 2 H, ArH), 6.91 (d, *J* = 8.71 Hz, 1 H, ArH), 7.17 (d, *J* = 8.50 Hz, 1 H, ArH), 7.34 (m, 1 H, ArH). ^13^C NMR (100 MHz, CDCl_3_): δ ppm 55.82, 56.02, 60.29, 62.92, 95.47, 101.34, 112.53, 123.57, 125.71, 128.13, 134.77, 135.35, 153.56, 155.75, 171.04 (C_2,_ C=O). HRMS: calculated for C_19_H_20_^35^ClFNO_5_ [M + H]^+^ 396.1014; found 396.1024.

#### 3.1.12. 4-(3-Bromo-4-methoxyphenyl)-3-fluoro-1-(3,4,5-trimethoxyphenyl)azetidin-2-one (**35**)

Preparation of compound **35** following the General Method II from imine **25** and ethyl bromofluoroacetate afforded the product as a brown oil; yield: 6%, purity (HPLC): 96%, IRν_max_ (ATR): 1743.0 (C=O) cm^−1^. ^1^H NMR (400 MHz, CDCl_3_): δ ppm 3.96 (s, 6 H, 2xOCH_3_), 3.70 (s, 3 H, OCH_3_), 3.72 (s, 3 H, OCH_3_) 5.29 (dd, *J* = 11.12, 1.70 Hz, 1 H, H3), 5.52 (dd, *J* = 4.90 Hz, 1 H, H4), 6.52 (s, 2 H, ArH), 7.10 (d, *J* = 3.40, 1H, ArH), 7.17 (d, *J* = 2.20 Hz 1 H, ArH), 7.53 (s, 1 H, ArH). ^13^C NMR (100 MHz, CDCl_3_): δ ppm 54.90, 55.57, 61.62, 65.55, 101.73, 102.44, 113.81, 125.28, 132.74, 134.22, 135.31, 136.25, 152.63, 154.78, 170.30 (C_2,_ C=O). HRMS: calculated for C_19_H_20_^79^BrFNO_5_ [M + H]^+^ 440.0509; found 440.0517.

#### 3.1.13. 3,3-Difluoro-4-(4-methoxyphenyl)-1-(3,4,5-trimethoxyphenyl)azetidin-2-one (**36**)

Preparation of compound **36** following the General Method II from imine **16** and ethyl bromodifluoroacetate afforded the product as a brown oil; yield: 59%, purity (HPLC): 98%. IR ν_max_ (ATR): 1765.8 (C=O) cm^−1^. ^1^H NMR (400 MHz, CDCl_3_): δ ppm 3.72–3.78 (m, 12 H, 4xOCH_3_), 5.30 (dd, *J* = 9.41, 1.66 Hz, 1 H, H4), 6.56 (s, 2 H, ArH), 6.88 (d, *J* = 9.12 Hz, 2 H, ArH), 6.94 (d, *J* = 9.12 Hz, 2 H, ArH). ^13^C NMR (100 MHz, CDCl_3_): δ ppm 55.57, 56.15, 60.20, 71.33, 100.29, 114.64, 125.85, 129.72, 134.25, 135.83, 136.16, 154.16, 158.22, 170.33 (C_2,_ C=O). HRMS: calculated for C_19_H_20_F_2_NO_5_ [M + H]^+^ 380.1310; found 380.1329.

#### 3.1.14. 4-(4-Ethoxyphenyl)-3,3-difluoro-1-(3,4,5-trimethoxyphenyl)azetidin-2-one (**37**)

Preparation of compound **37** following the General Method II from imine **17** and ethyl bromodifluoroacetate afforded the product as a brown oil; yield: 23%, purity (HPLC): 98%. IR ν_max_ (ATR): 1734.4 (C=O) cm^−1^. ^1^H NMR (400 MHz, CDCl_3_): δ ppm 1.40 (t, *J* = 7.05 Hz, 3 H, CH_2_CH_3_), 3.70 (s, 6 H, 2xOCH_3_), 3.76 (s, 3 H, OCH_3_), 4.03 (q, *J* = 7.33 Hz, 2 H, CH_2_CH_3_), 5.28 (dd, *J* = 12.20, 1.64 Hz, 1 H, H4), 6.56 (s, 2 H, ArH), 6.91 (d, *J* = 8.71 Hz, 2 H, ArH), 7.27 (d, *J* = 8.71 Hz, 2 H, ArH). ^13^C NMR (100 MHz, CDCl_3_): δ ppm 14.70, 56.08, 60.92, 63.59, 95.97, 115.10, 121.47, 128.94, 153.58, 160.19 (C_2,_ C=O). HRMS: calculated for C_20_H_22_F_2_NO_5_ [M + H]^+^ 394.1466; found 394.1478.

#### 3.1.15. 3,3-Difluoro-4-(4-(methylthio)phenyl)-1-(3,4,5-trimethoxyphenyl)azetidin-2-one (**38**)

Preparation of compound **38** following the General Method II from imine **18** and ethyl bromodifluoroacetate afforded the product as an oil; yield: 19%, purity (HPLC): 96%. IR ν_max_ (ATR): 1734.3 (C=O) cm^−1^. ^1^H NMR (400 MHz, CDCl_3_): δ ppm 2.38 (s, 3 H, CH_3_), 3.61–3.69 (m, 9 H, 3xOCH_3_), 4.94 (dd, *J* = 12.87, 1.66 Hz, 1 H, H4), 5.82 (s, 2 H, ArH), 7.16 (m, *J* = 8.29 Hz, 2 H, ArH), 7.28 (m, *J* = 8.29 Hz, 2 H, ArH). ^13^C NMR (100 MHz, CDCl_3_): δ ppm 14.07, 60.34, 60.82, 62.88, 63.21, 92.02, 126.25, 127.92, 128.62, 129.73, 130.48, 130.98, 139.60, 153.71, 171.17 (C_2,_ C=O). HRMS: calculated for C_19_H_20_F_2_NO_4_S [M + H]^+^ 396.1081; found 396.1100.

#### 3.1.16. 4-(4-(Ethylthio)phenyl)-3,3-difluoro-1-(3,4,5-trimethoxyphenyl)azetidin-2-one (**39**)

Preparation of compound **38** following the General Method II from imine **19** and ethyl bromodifluoroacetate afforded the product as a brown oil; yield: 18%, purity (HPLC): 99%. IR ν_max_ (ATR): 1774.5 (C=O) cm^−1^. ^1^H NMR (400 MHz, CDCl_3_): δ ppm 1.29 (t, *J* = 7.46 Hz, 3 H, SCH_2_CH_3_), 2.90–2.95 (q, *J* = 7.46 Hz, 2 H, SCH_2_CH_3_), 3.67 (s, 3 H, OCH_3_), 3.74 (s, 6 H, 2xOCH_3_), 5.31 (dd, *J* = 7.05, 1.66 Hz, 1 H, H4), 6.53 (s, 2 H, ArH), 7.24 (m, *J* = 8.71 Hz, 2 H, ArH), 7.30 (m, *J* = 8.29 Hz, 2 H, ArH). ^13^C NMR (100 MHz, CDCl_3_): δ ppm 14.02, 26.74, 56.05, 60.36, 60.90, 95.88, 126.72, 127.54, 128.26, 131.60, 135.78, 139.83, 142.29, 153.59, 171.15 (C_2,_ C=O). HRMS: calculated for C_20_H_22_F_2_NO_4_S [M + H]^+^ 410.1238; found 410.1219.

#### 3.1.17. 3,3-Difluoro-4-(4-methoxy-3-methylphenyl)-1-(3,4,5-trimethoxyphenyl)azetidin-2-one (**40**)

Preparation of compound **40** following the General Method II from imine **20** and ethyl bromodifluoroacetate afforded the product as a brown oil; yield: 31%, purity (HPLC): 95%. IR ν_max_ (ATR): 1769.9 (C=O) cm^−1^. ^1^H NMR (400 MHz, CDCl_3_): δ ppm 2.16 (s, 3 H, CH_3_), 3.66 (s, 3 H, OCH_3_), 3.72 (s, 3H, OCH_3_), 3.77 (s, 6H, 2xOCH_3_), 5.80 (dd, *J* = 7.46, 1.66 Hz, 1 H, H4), 6.55 (s, 2 H, ArH), 6.80 (d, *J* = 8.29 Hz, 1 H, ArH), 7.08 (s, 1 H, ArH), 7.12 (dd, *J* = 8.29, 2.07 Hz, 1 H, ArH).^13^C NMR (100 MHz, CDCl_3_): δ ppm 16.20, 55.99, 60.79, 69.19, 96.00, 110.13, 121.10, 126.44, 127.68, 129.58, 131.79, 135.70, 136.18, 154.84, 157.92, 171.04 (C_2,_ C=O). HRMS: calculated for C_20_H_22_F_2_NO_5_ [M + H]^+^ 394.1466; found 394.1478.

#### 3.1.18. 4-(3-(tert-Butyldimethylsilyl)-4-methoxyphenyl)-3,3-difluoro-1-(3,4,5-trimethoxyphenyl)azetidin-2-one (**41**)

Preparation of compound **41** following the General Method II from imine **22** and ethyl bromodifluoroacetate afforded the product as a yellow oil; yield: 13%, purity (HPLC): 100%. IR ν_max_ (ATR): 1775.6 (C=O) cm^−1^. ^1^H NMR (400 MHz, CDCl_3_): δ ppm 0.25 (d, *J* = 7.46 Hz, 6 H, 2xSi-CH_3_), 0.91 (s, 9 H, 3xCH_3_), 3.68 (s, 3 H, OCH_3_), 3.75 (s, 6 H, 2xOCH_3_), 5.25 (dd, *J* = 7.46, 1.66 Hz, 1 H, H_4_), 6.56 (s, 2 H, ArH), 6.77 (d, *J* = 2.07 Hz, 1 H, ArH), 6.86 (d, *J* = 8.29 Hz, 1 H, ArH), 6.92 (dd, *J* = 8.29, 2.07 Hz, 1 H, ArH). ^13^C NMR (101 MHz, CDCl_3_) δ ppm -1.5 (Si-CH_3_), 18.39 (CH_3_), 25.54 (Si-C-CH_3_), 55.41, 56.02, 60.88, 68.83, 96.02, 112.19, 119.98, 121.38, 121.94, 128.17, 131.69, 135.72, 145.60, 152.31, 153.55, 170.79 (C_2,_ C=O). HRMS: [M+H]^+^ calculated for C_25_H_34_F_2_NO_5_Si [M + H]^+^ 494.6306; found 494.6289.

#### 3.1.19. 3,3-Difluoro-4-(3-fluoro-4-methoxyphenyl)-1-(3,4,5-trimethoxyphenyl)azetidin-2-one (**43**)

Preparation of compound **43** following the General Method II from imine **23** and ethyl bromodifluoroacetate afforded the product as a colourless oil; yield: 45%, purity (HPLC): 97%. IRν_max_ (ATR): 1778.1 (C=O) cm^−1^. ^1^H NMR (400 MHz, CDCl_3_): δ ppm 3.58 (s, 3 H, OCH_3_), 3.64 (s, 9 H, 3xOCH_3_), 5.28 (dd, *J* = 6.63, 1.24 Hz, 1 H, H4), 6.46 (s, 2 H, ArH), 6.95–7.09 (m, 3 H, ArH). ^13^C NMR (100 MHz, CDCl_3_): δ ppm 55.62, 55.84, 60.16, 63.09, 95.89, 115.23, 123.90, 126.81, 130.98, 131.35, 135.80, 148.80, 151.12, 153.55, 170.93 (C_2,_ C=O). HRMS: calculated for C_19_H_19_F_3_NO_5_ [M + H]^+^ 398.1215; found 398.1200.

#### 3.1.20. 4-(3-Chloro-4-methoxyphenyl)-3,3-difluoro-1-(3,4,5-trimethoxyphenyl)azetidin-2-one (**44**)

Preparation of compound **44** w following the General Method II from imine **24** and ethyl bromodifluoroacetate afforded the product as a brown oil; yield: 59%, purity (HPLC): 95%. IR ν_max_ (ATR): 1774.80 (C=O) cm^−1^. ^1^H NMR (400 MHz, CDCl_3_): δ ppm 3.61 (s, 3 H, OCH_3_), 3.66 (s, 6 H, 2xOCH_3_), 3.78 (s, 3 H, OCH_3_), 5.29 (dd, *J* = 7.46, 1.25 Hz, 1 H, H4), 6.47 (s, 2 H, ArH), 6.89 (d, *J* = 8.71 Hz, 1 H, ArH), 7.16 (dd, *J* = 8.71, 2.07 Hz, 1 H, ArH), 7.31 (d, *J* = 2.07 Hz, 1 H, ArH). ^13^C NMR (100 MHz, CDCl_3_): δ ppm 55.91, 56.03, 60.69, 68.18, 91.90, 112.37, 122.68, 127.82, 129.24, 131.36, 135.71, 153.53, 156.10, 171.24 (C_2_, C=O). HRMS: calculated for C_19_H_19_^35^ClF_2_NO_5_ [M + H]^+^ 414.0920; found 414.0926.

#### 3.1.21. 4-(3-Bromo-4-methoxyphenyl)-3,3-difluoro-1-(3,4,5-trimethoxyphenyl)azetidin-2-one (**45**)

Preparation of compound **45** following the General Method II from imine **25** and ethyl bromodifluoroacetate afforded the product as a yellow oil; yield: 65%, purity (HPLC): 98%. IRν_max_ (ATR): 1774.5cm^−1^ (C=O, β-lactam). ^1^H NMR (400 MHz, CDCl_3_): δ ppm 3.54 (s, 6 H, 2xOCH_3_), 3.57 (s, 3 H, OCH_3_), 3.66 (s, 3 H, OCH_3_), 5.81 (dd, *J* = 7.26, 1.86 Hz, 1 H, H_4_), 6.43 (s, 2 H, ArH), 6.80 (s, 1 H, ArH), 7.14 (s, 1 H, ArH), 7.45 (s, 1 H, ArH). ^13^C NMR (100 MHz, CDCl_3_): δ ppm 54.80, 56.75, 60.54, 91.91, 111.59, 112.12, 127.50, 127.56, 130.77, 133.06, 133.18, 135.67, 153.50, 156.93, 170.95 (C_2,_ C=O). HRMS: [M + H]^+^ calculated for C_19_H_19_^79^BrF_2_NO_5_, 458.0415; found 458.0432.

#### 3.1.22. 3-Fluoro-4-(3-hydroxy-4-methoxyphenyl)-1-(3,4,5-trimethoxyphenyl)azetidin-2-one (**32**)

The TBDMS-protected β-lactam **31** (1 mmol) was dissolved under nitrogen in THF. The solution was stirred on ice enabling it to reach 0 °C. *tert*-Butylammonium fluoride (TBAF) solution (1.0 M, 1.5 equivalents) was added slowly dropwise. The resulting solution was stirred at 0 °C and monitored by TLC until completion of the reaction when the starting material was no longer present (1 h). The reaction mixture was diluted with ethyl acetate (50 mL) and washed with HCl (25 mL, 0.1 M) followed by extraction of the aqueous mixture using ethyl acetate (2 × 25 mL). The combined organic extracts were washed with water (100 mL) and saturated brine (100 mL) and dried over Na_2_SO_4_. The solution was concentrated *in vacuo*, and the crude product was purified by flash chromatography over silica gel (hexane:ethyl acetate gradient). The phenolic product **32** was obtained as a brown oil; 18%, purity (HPLC): 100%. IR ν_max_ (ATR): 3464.54, 1771.07 (OH and C=O) cm^−1^. ^1^H NMR (400 MHz, CDCl_3_): δ ppm 3.72 (s, 3H), 3.74 (s, 6H), 3.87 (s, 3H), 4.86 (apparent dd, ^3^*J*_F-H_ = 26.1 Hz, ^3^*J* = 2.6 Hz, 1H, H_4_), 5.11 (apparent dd, 1H,^2^*J*_F-3_ = 48.3 Hz, ^3^*J* = 2.6 Hz, H_3_), 5.83 (s, 2H), 6.83 (d, 1H, ^3^*J* = 8.9 Hz, Ar-H), 6.88 (dd, 1H, ^3^*J* = 8.1 Hz, ^4^*J* = 1.9 Hz), δ 6.98 (d, 1H, ^4^*J* = 1.9 Hz) ^13^C NMR (100 MHz, CDCl_3_) δ ppm 55.9, 56.0, 58.8 (d, *J*_F-C_ = 26 Hz, C_4_), 61.0, 62.1, 92.3, 95.2 (d, *J*_F-C_ = 75 Hz, C_4_), 110.8, 113.1, 118.6, 130.4, 131.4, 142.8, 146.2, 153.8, 167.9 (*J*_F-C_ = 31 Hz, C_2_). ^19^F NMR (376 MHz CDCl_3_): δ -203.6. HRMS: calculated for C_19_H_21_FNO_6_ [M + H]^+^, 378.1353; found 378.1347.

#### 3.1.23. 3,3-Difluoro-4-(3-hydroxy-4-methoxyphenyl)-1-(3,4,5-trimethoxyphenyl)azetidin-2-one (**42**)

Compound **42** was prepared from the TBDMS-protected β-lactam **41** using the procedure described above for **20** to obtain the phenolic product as a yellow oil; yield: 21%, purity (HPLC): 100%. IR ν_max_ (ATR): 3426.75, 1771.07 (OH, C=O) cm^−1^. ^1^H NMR (400 MHz, CDCl_3_): δ ppm 3.69 (s, 6 H, 2xOCH_3_), 3.75 (s, 3 H, OCH_3_), 3.87 (s, 3 H, OCH_3_), 5.25 (d, *J* = 7.05 Hz, 1 H, H4), 6.36 (s, 1 H, OH), 6.56 (s, 2 H, ArH), 6.85 (s, 2 H, ArH), 6.89 (s, 1 H, ArH). ^13^C NMR (100 MHz, CDCl_3_): δ ppm 56.10, 60.89, 69.13, 96.02, 110.96, 113.57, 119.61, 122.74, 125.46, 131.72, 135.75, 146.25, 147.82, 153.56, 171.42 (C_2_, C=O). HRMS: calculated for C_19_H_20_F_2_NO_6_ [M + H]^+^, 396.1251; found 396.1253.

### 3.2. Biochemical Evaluation of Compounds

The biochemical assays were performed in triplicate on at least three independent occasions for the determination of mean values reported.

#### 3.2.1. Cell Culture

The human breast carcinoma cell lines MCF-7 and MDA-MB-231 were purchased from the European Collection of Animal Cell Cultures (ECACC, Salisbury, UK). The SW-480 cells were a kind gift from Dr. Brian Flood, School of Biochemistry and Immunology, Trinity College Dublin. The human breast tumour cell line MCF-7 was cultured in Eagles minimum essential medium with 10% foetal bovine serum, 2 mM L-glutamine and 100 μg/mL penicillin/streptomycin. The medium was supplemented with 1% non-essential amino acids. The human breast tumour cell line MDA-MB-231 cells were maintained in Dulbecco’s modified Eagle’s medium (DMEM) with the addition of penicillin/streptomycin (100 µg/mL), 10% (*v*/*v*) foetal bovine serum and 2 mM L-glutamine (complete medium). Breast cancer cell line Hs578T and its isogenic subclone Hs578T(i)8 were a kind gift from Dr. Susan McDonnell, School of Chemical and Bioprocess Engineering, University College Dublin. SW-480 cells, triple-negative breast cancer Hs578T cells and invasive variant Hs578T8i cells were cultured in DMEM with GlutaMAX-I, with the same supplement in the absence of non-essential amino acids. HEK-293T cells (normal epithelial embryonic kidney cells) were cultured in DMEM with GlutaMAX-I in the absence of non-essential amino acids. The SW-480 cells were cultured in DMEM with GlutaMAX-I, with the same supplement in the absence of non-essential amino acids. All media contained 100 U/mL penicillin and 100 μg/mL streptomycin. The cells were seeded into pre-warmed complete medium (10 × 10^4^ cells/mL) and maintained at a cell density of 2–19 × 10^5^ cells /mL by the addition of fresh media. The cell number was monitored using a haemocytometer. Cells were maintained at 37 °C in 5% CO_2_ in a humidified incubator. The cells were sub-cultured 3 times weekly by trypsinisation using TrypLE Express (1X).

#### 3.2.2. Cell Viability Assay

Cells were seeded at a density of 5 × 10^3^ cells/well (MCF-7 and MDA-MB-231) and 1 × 10^4^ cells/well (HEK-293T) in 96-well plates (200 μL per well). After 24 h, cells were then treated with medium alone, with vehicle (1% ethanol (*v*/*v*)) or with CA-4 or β-lactam compounds at 1 μM and 10 μM concentrations. For determination of IC_50_ values, serial dilutions of CA-4 or β-lactam compounds **26**–**31**, **33**–**41** and **43**–**45** (0.001–100 μM) in triplicate were used. Cell proliferation for MCF-7 cells was analysed with the AlamarBlue assay (Invitrogen Corp.) as previously described by us [33]. After 72 h, AlamarBlue (10% (*v*/*v*)) was added to each well, and plates were incubated for 3–5 h at 37 °C in the dark. Fluorescence results were obtained with a 96-well fluorimeter with excitation at 530 nm and emission at 590 nm with results recorded as percentage viability relative to vehicle control (100%). Dose–response curves were obtained and IC_50_ values (concentration of drug resulting in 50% reduction in cell survival) were calculated using Prism (GraphPad Software, Inc., La Jolla, CA, USA).

#### 3.2.3. Annexin V/PI Apoptotic Assay

Apoptotic cell death was monitored by flow cytometry using Annexin V and propidium iodide (PI) as previously described by us [33]. MCF-7 cells were seeded in 6-well plates at a density of 1 × 10^5^ cells/mL and treated with either vehicle (0.1% (*v*/*v*) EtOH) or β-lactam compound **33** at concentrations 0.1 and 0.5 µM for 48 h. Cells were harvested and prepared for flow cytometric analysis. Cells were washed in 1X binding buffer (20X binding buffer: 0.1 M HEPES, pH 7.4; 1.4 M NaCl; 25 mM CaCl_2_ diluted in dH_2_O) and incubated in the dark for 30 min on ice in Annexin V-containing binding buffer [1:100]. Cells were then washed in binding buffer and then re-suspended in PI-containing binding buffer (1:1000). Samples were then analysed with the BD Accuri flow cytometer and Prism software. The resulting cell populations were identified as Annexin V- and PI-negative (Q4, healthy cells), Annexin V-positive and PI-negative (Q3, early apoptosis), Annexin V- and PI-positive (Q2, late apoptosis) and Annexin V-negative and PI-positive (Q1, necrosis). The positive control for induction of cell death was paclitaxel.

#### 3.2.4. Immunofluorescence Microscopy

Confocal microscopy was used to determine the effects of novel compound **33** and standard drugs on the MCF-7 cytoskeleton according to the protocols we previously described [33]. For the immunofluorescence study, MCF-7 cells were seeded at 1 × 10^5^ cells/mL using eight chamber glass slides (BD Biosciences). The cells were treated with vehicle control (1% ethanol (*v*/*v*)), CA-4 (0.01 μM), paclitaxel (1 µM) and **33** (0.1 µM, 0.5 µM and 1 µM) for 16 h. The cells were gently washed in PBS, fixed with 4% paraformaldehyde in PBS (20 min) and permeabilised in 0.5% Triton X-100. After further washes in PBS (containing 0.1% Tween (PBST)), cells were blocked with 5% bovine serum albumin which was diluted with PBST. Cells were incubated with mouse monoclonal anti-α-tubulin–FITC antibody (clone DM1A) (Sigma) (1:100) at 20 °C for 2 h and then washed in PBST and incubated with Alexa Fluor 488 dye (1:500) for 1 h at 20 °C. Cells were washed in PBST and mounted in Ultra Cruz Mounting Media (Santa Cruz Biotechnology, Santa Cruz, CA, USA) containing 4,6-diamino-2-phenolindol dihydrochloride (DAPI). The images were captured by Leica SP8 confocal microscopy with Leica Application Suite X software. Experiments were performed on three independent occasions and images were collected on the same day using identical parameters.

#### 3.2.5. Evaluation of Expression Levels of Anti-Apoptotic Protein Bcl-2 and Pro-Apoptotic Proteins Bax and Survivin

Western blot analysis was performed according to the protocols we previously described [33]. MCF-7 cells were seeded at a density of 1 × 10^5^ cells/flask in T25 flasks. After 48 h, whole cell lysates were prepared from untreated cells or cells treated with vehicle control (EtOH, 0.1% *v*/*v*) or cells treated with compound **33** (0.05 μM, 0.1 μM and 0.5 μM). MCF-7 cells were harvested in RIPA buffer which was supplemented with protease inhibitors (Roche Diagnostics, Rotkreuz, Switzerland), phosphatase inhibitor cocktail 2 (Sigma-Aldrich, Arklow, Ireland) and phosphatase inhibitor cocktail 3 (Sigma-Aldrich, Arklow, Ireland). Equal amounts of protein (as determined by a BCA assay) were resolved by SDS-PAGE (12%) followed by transfer to PVDF membranes. Membranes were blocked in 5% bovine serum albumin/TBST for 1 h and incubated in the relevant primary antibodies at 4 °C overnight, washed with TBST, and incubated in horseradish peroxidase conjugated secondary antibody for 1 h at 20 °C. After washing, Western blot analysis was performed with antibodies directed against Bcl-2 (1:500) (Millipore), Bax (1:1000) (Millipore) or survivin (1:1000) (Millipore) and followed by incubation with a horseradish peroxidase conjugated anti-mouse antibody (1:2000) (Promega, Madison, WI, USA). The blots were probed with anti-GAPDH antibody (1:5000) (Millipore) to confirm equal loading of protein. Proteins were detected using enhanced chemiluminescent Western blot detection (Clarity Western ECL substrate) (Bio-Rad) on the ChemiDoc MP System (Bio-Rad). Experiments were performed on three independent occasions.

#### 3.2.6. Tubulin Polymerisation Assay

Tubulin polymerisation was monitored with purified bovine tubulin using the BK006 kit supplied by Cytoskeleton Inc. (Denver, CO, USA), carried out following the manufacturer’s instructions with the standard assay conditions [95]. Purified (>99%) bovine brain tubulin (3 mg/mL) in a buffer containing PIPES (80 mM, pH 6.9), EGTA (0.5 mM), MgCl_2_ (2 mM), GTP (1 mM) and glycerol (10%) was incubated at 37 °C in the presence of either vehicle (2% (*v*/*v*) ddH_2_O) or paclitaxel (10 µM), CA-4 (10 μM) or **33** (10 and 30 µM). The effect on tubulin assembly was monitored turbidimetrically at 340 nm in a Spectramax 340 PC spectrophotometer (Molecular Devices, Sunnyvale, CA, USA), with the light scattered depending on the concentration of polymerised microtubules formed. The absorbance was measured at 30 s intervals for 60 min.

#### 3.2.7. Stability Study for Compounds **33** and **39**

The stability study for compounds **33** and **39** was performed using analytical HPLC (Symmetry column (C18, 5 mm, 4.6 × 150 mm), dual wavelength absorbance detector (Waters 2487), binary HPLC pump (Waters 1525) and autosampler (Waters 717 plus)) with mobile phase acetonitrile (70%)/water (30%), flow rate 1 mL/min over 15 min and detection at λ 254 nm. Stock solutions of compounds **33** and **39** (1 mg/mL in mobile phase) were used. Dilutions of 0.5 mg/mL, 0.25 mg/mL, 0.125 mg/mL, 0.0625 mg/mL, 0.03125 mg/mL, 0.015625 mg/mL and 0.0078 mg/mL were prepared for the calibration curve. (i) *Stability of* **33** *and* **39** *in phosphate buffers*: The phosphate buffers were prepared in accordance with the British Pharmacopoeia 2020 at pH values 4, 7.4 and 9. Stock solution, 300 µL (1 mg/mL ACN), was added to a vial containing buffer (9.7 mL), mixed and pre-heated to 37 °C. 1 mL of the solution was introduced into an HPLC vial. Samples (10 µL) were injected and analysed at time intervals of t = 0 min, 5 min, 30 min, 60 min and hourly for 24 h. The analysis was performed in triplicate. (ii) *Thermal stability*: Compounds **33** and **39** (1 mL stock solution) were each placed in a vial for 4 h at 60 °C on a heating block. The sample was then cooled, diluted with acetonitrile and analysed by HPLC. (iii) *Photostability study*: Compounds **33** and **39** (stock solution, 1 mL) were placed in a vial, exposed to UV light for 4 h and then analysed by HPLC. (iv) *Stability in acidic conditions:* HCl (0.1 M, 0.2 mL) was added to stock solutions (0.8 mL) of **33** and **39** in a vial. The vial was vortexed and retained at room temperature. A sample was neutralised with NaOH (0.1 M, 0.2 mL) each hour for 4 h and then analysed by HPLC. (v) *Stability of* **33** *and* **39** *in basic (alkaline) conditions*: NaOH (0.1 M, 0.2 mL) was added to stock solution (0.8 mL) in a vial. The vial was vortexed and retained at room temperature. A sample was neutralised with HCl (0.1 M, 0.2 mL) every hour for 4 h then analysed by HPLC. (vi) *Stability of* **33** *and* **39** *in oxidising conditions*: H_2_O_2_ (3%, 0.2 mL) was added to the stock solution (0.8 mL). The vial was vortexed to obtain a homogeneous mixture and retained at room temperature, and a sample was analysed by HPLC every hour for 4 h.

#### 3.2.8. Computational Procedure

The X-ray structure of bovine tubulin co-crystallised with N-deacetyl-N-(2-mercaptoacetyl)-colchicine (DAMA-colchicine) was downloaded from the PDB website (entry 1SA0) [19]. A UniProt Align analysis confirmed a 100% sequence identity between human and bovine β-tubulin. The crystal structure was prepared using QuickPrep (minimised to a gradient of 0.001 kcal/mol/Å), Protonate 3D, Residue pKa and Partial Charges protocols in MOE 2020 using the MMFF94x force field. Compounds **32**, **33**, **42** and **43** were drawn in MOE, saved as an mdb file and processed in MOE. Both *trans* enantiomers of compounds **32** and **33** were examined in addition to both enantiomers of compounds **42** and **43**. MMFF94x partial charges were calculated for each compound and minimised to a gradient of 0.001 kcal/mol/Å. Default parameters were used for docking; 300 poses were sampled for each compound and the top 50 docked poses were retained for subsequent analysis. Default settings of OMEGA [92,93] were used to generate 50 conformers of each compound prior to running rigid docking with FRED [94], included in the OEDocking suite (OpenEye Scientific Software, Santa Fe, NM, USA; http://www.eyesopen.com) (accessed on 15 September 2021).

### 3.3. X-ray Crystallography Analysis

Data were collected on a Bruker APEX DUO for samples **18**, **23** and **33** using Mo Kα radiation (λ = 0.71073 Å) and **43** using Cu Kα radiation (λ = 1.54178 Å). Each sample was mounted on a MiTeGen cryoloop, and data were collected at 100(2) K using an Oxford Cobra cryosystem. Bruker APEX [96] software was used to collect and reduce data and determine the space group. Absorption corrections were applied using SADABS [97].

All structures were solved with the SHELXT [98] structure solution program using Intrinsic Phasing and refined using the least squares method on F^2^ with SHELXL [99]. All non-hydrogen atoms were refined anisotropically. Hydrogen atoms were assigned to calculated positions using a riding model with appropriately fixed isotropic thermal parameters. Molecular graphics were generated using OLEX2 [100].

Crystallographic data for the structures in this paper have been deposited with the Cambridge Crystallographic Data Centre as supplementary publication Nos. 2101671, 2101672, 1537939 and 1537940. Copies of the data can be obtained, free of charge, on application to CCDC, 12 Union Road, Cambridge CB2 1EZ, UK (Fax: +44-(0)1223-336033 or e-mail: deposit@ccdc.cam.ac.uk).

## 4. Conclusions

Microtubule-targeting agents are the group of drugs frequently used for cancer treatment both in adults and children; they are effective in suppressing microtubule dynamics and inducing cell death via the mitochondrial intrinsic apoptosis pathway. Significant progress has been achieved in the discovery of targeted cancer therapies; however, it is recognised that resistance (demonstrated by both innate and acquired mechanisms) remains an issue for many clinically successful cancer drugs [32]. The β-lactam scaffold continues to attract much interest due to its utility as a versatile synthetic intermediate and also the many therapeutic applications of this heterocycle. We have now described the synthesis of a series of novel 3-fluoro and 3,3-difluoro-β-lactams which are designed as CA-4 analogues that show significant antiproliferative activity against the MCF-7 cell line. Introduction of fluorine can enhance the potency and target selectivity of a drug by affecting properties such as pKa, lipophilicity, hydrophobic interactions and membrane permeability. In the present work, compounds **32** and **33** were the most potent in this series examined, with IC_50_ values of 0.075 and 95 µM, respectively, in the MCF-7 cell line. The phenolic compound **32** also demonstrated significant antiproliferative activity at nanomolar concentrations in the triple-negative breast cancer cell line Hs578T (IC_50_ 0.033 μM), together with potency in the invasive isogenic subclone Hs578Ts(i)8 (IC_50_ = 0.065 μM), while **33** was also effective in MDA-MB-231 cells (IC_50_ 0.620 μM). The introduction of the fluorine substituent at C-3 of the β-lactam ring resulted in the antiproliferative profile for compounds **32** and **33** comparable to our previously reported 3-chloro, 3-vinyl and 3-methylazetidin-2-ones as CA-4 analogues. The 3-fluoro compounds demonstrated significant antiproliferative activity at nanomolar concentrations in the NCI 60 cancer cell line panel across a range of human cancer cell lines. Selectivity of the compounds in targeting cancer cells was demonstrated with minimal cytotoxicity shown in non-tumourigenic cell line HEK-293T on treatment with the 3-fluoro-β-lactam compound **33**.

The mechanism of action of these compounds was investigated. Induction of apoptosis was confirmed for compound **33** using flow cytometric analysis of Annexin V/PI-stained cells. Additionally, β-lactam **33** was observed to inhibit tubulin polymerisation, causing disruption of tubulin network structure in MCF-7 cells, inducing a disorganised microtubule network with multinucleation effects as also observed for CA-4. The effects on the expression of the characteristic apoptosis-related proteins Bcl-2, Bax and survivin in MCF-7 cells on treatment with compound **33** were demonstrated with Western blot analysis and confirmed the pro-apoptotic action of the 3-fluoro β-lactam **33**. The tubulin-targeting effects of compound **33** were demonstrated in a molecular modelling study suggesting interactions of the compound’s A and B rings with the colchicine-binding site of β-tubulin, in a manner similar to that of CA-4. The 3-fluoro-β-lactams **32** and **33** demonstrated potential as lead microtubule-targeting molecules suitable for further preclinical anticancer drug development.

## Figures and Tables

**Table 1:** X-ray crystal structures of imines **18** and **23**.

Compound	Structure	X-ray Representation
**18**	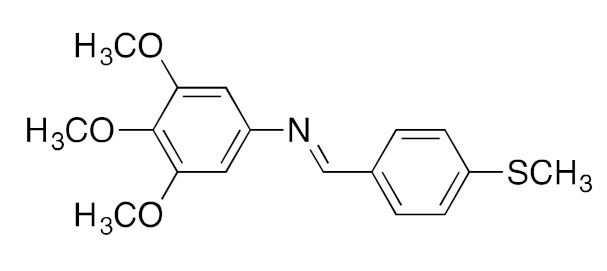	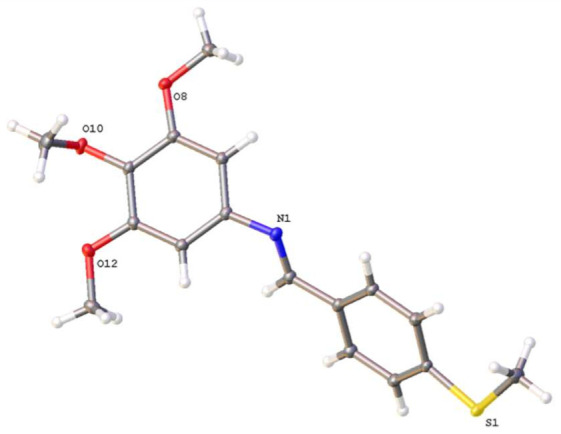
**23**	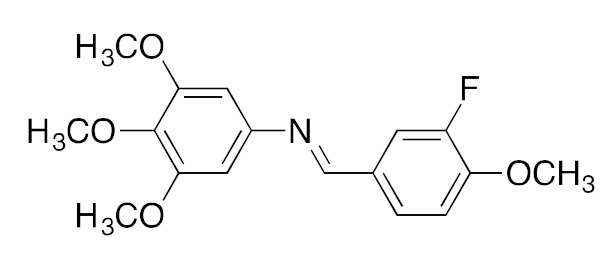	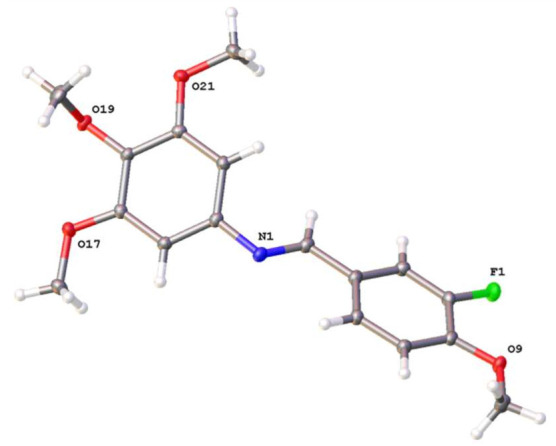

ORTEP representation of the X-ray crystal structures of imines **18** and **23** with 50% thermal ellipsoids with heteroatoms numbered.

**Table 2:** X-ray crystal structures of β-lactams **33** and **43** ^a^.

Compound	Structure	X-ray Representation
**33**	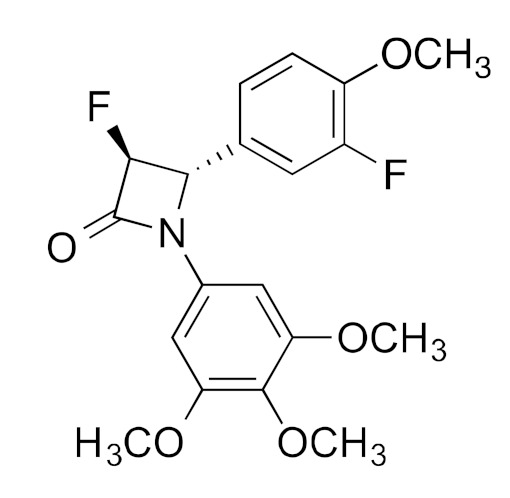	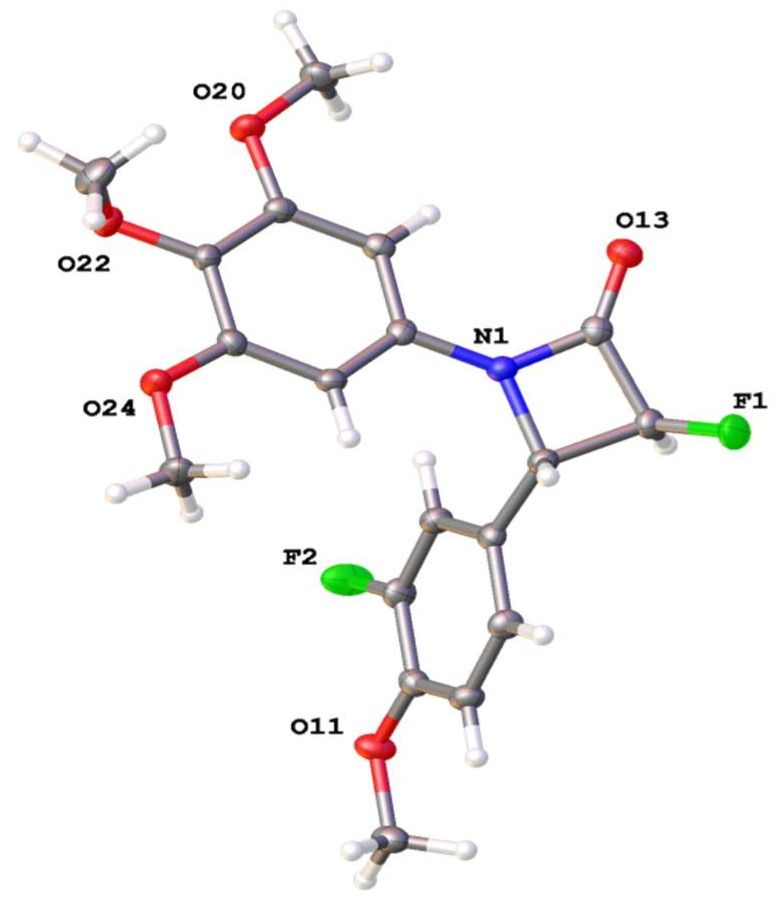
**43**	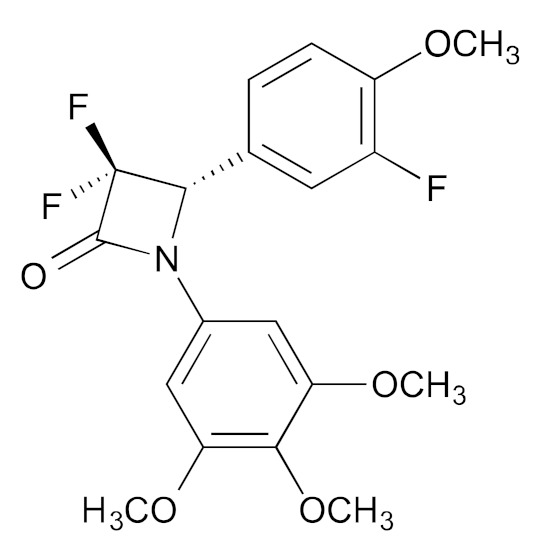	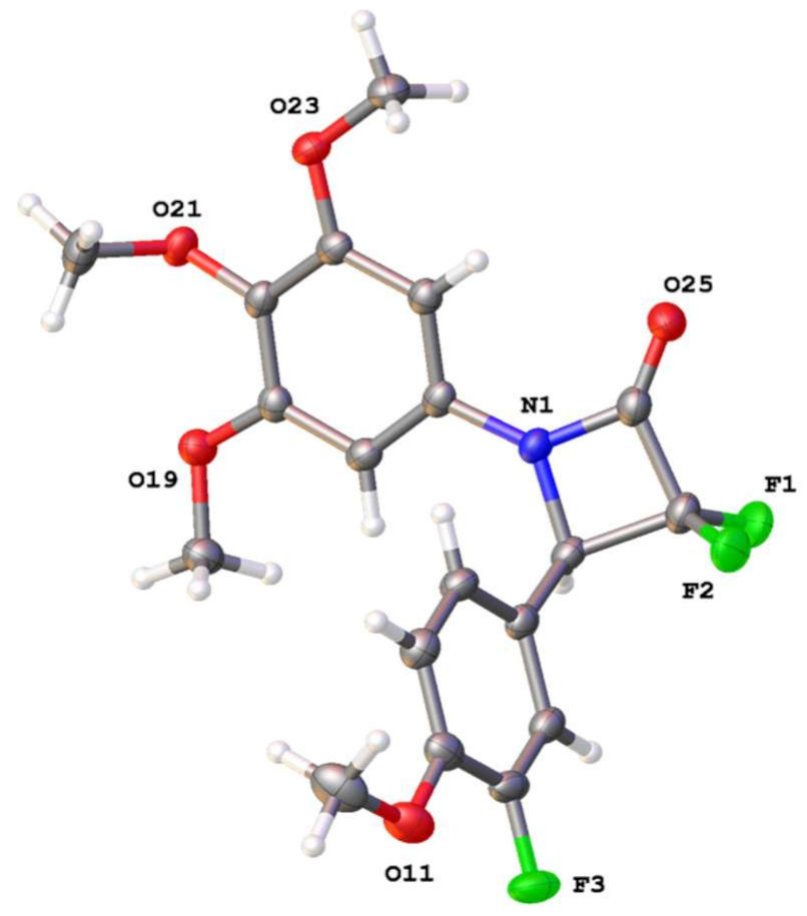

^a^ ORTEP representation of the X-ray crystal structures of *meta*-fluoro ring B β-lactams **33** and **43** with 50% thermal ellipsoids with heteroatoms numbered.

**Table 3:** Crystal data and structure refinement details for imines **18** and **23**, 3-fluoro β-lactam **33** and 3,3-difluoro β-lactam **43**.

Identification Code	18	23	33	43
CCDC number	2101671	2101672	1537939	1537940
Empirical formula	C_17_H_19_NO_3_S	C_17_H_18_NO_4_F	C_19_H_19_F_2_NO_5_	C_19_H_18_F_3_NO_5_
*M* (g/mol)	317.39	319.32	379.35	397.34
*T* (K)	100 (2)	100 (2)	100 (2)	100 (2)
Crystal system	Monoclinic	Monoclinic	Monoclinic	Triclinic
SG	P2_1_/c	P2_1_/c	P2_1_/n	P$\bar{1}$
*a* (Å)	14.1344 (4)	14.2025 (3)	9.9882 (4)	9.2060 (3)
*b* (Å)	15.5489 (4)	15.1955 (4)	9.9623 (4)	9.6842 (3)
*c* (Å)	7.2569 (2)	7.2677 (2)	18.1462 (7)	12.3233 (4)
*α* (°)	90	90	90	104.832 (2)
*β* (°)	93.9918 (10)	97.3324 (8)	97.1621 (13)	96.939 (2)
*γ* (°)	90	90	90	117.376 (2)
*V* (Å^3^)	1591.01 (8)	1555.65 (7)	1791.56 (12)	905.97 (5)
*Z*	4	4	4	2
*Dcalc* (g/cm^3^)	1.325	1.363	1.406	1.457
*μ* (mm^−1^)	0.215	0.105	0.115	1.083
F(000)	672	672.0	792.0	412.0
Radiation	Mo Kα (λ = 0.71073)	Mo Kα (λ = 0.71073)	Mo Kα (λ = 0.71073)	Cu Kα (λ = 1.54178)
Reflections collected	96322	73658	30695	11320
Independent reflections	4710 R_int_ = 0.0376, R_sigma_ = 0.0139	4123 R_int_ = 0.0304, R_sigma_ = 0.0105	5242 R_int_ = 0.0417, R_sigma_ = 0.0363	3379 R_int_ = 0.0289, R_sigma_ = 0.0266
Data/restraints/parameters	4710/0/199	4123/0/216	5242/0/248	3379/0/257
Goodness-of-fit on *F*^2^ (*S*)	1.039	1.074	1.023	1.060
Final *R* indexes [*I* ≥ 2σ (*I*)] *	*R*_1_ = 0.0323, *wR*_2_ = 0.0824	*R*_1_ = 0.0332, *wR*_2_ = 0.0950	*R*_1_ = 0.0393, *wR*_2_ = 0.0886	*R*_1_ = 0.0404, *wR*_2_ = 0.1138
Final *R* indexes (all data)	*R*_1_ = 0.0437, *wR*_2_ = 0.0899	*R*_1_ = 0.0404, *wR*_2_ = 0.1014	*R*_1_ = 0.0695, *wR*_2_ = 0.0999	*R*_1_ = 0.0452, *wR*_2_ = 0.1186
Largest diff. peak/hole/e Å^−3^	0.44/−0.32	0.37/−0.20	0.38/−0.26	0.27/−0.27

* *R*_1_ = ∑||*F*_o_| − |*F*_c_||/∑|*F*_o_|, *wR*_2_ = [∑*w*(*F*_o_^2^ − *F*_c_^2^)^2^/∑*w*(*F*_o_^2^)^2^]^1/2^.

**Table 4:** X-ray crystallographic data for β-lactams 3-fluoro β-lactam **33** and 3,3-difluoro β-lactam **43**.

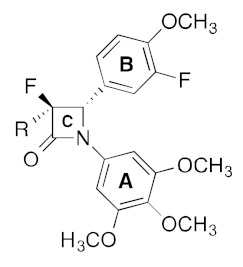	Ring Plane Normal AB Angle (°)	Ring A to Central Torsion (°) ^a^*	Ring B to Central Torsion (°) ^b^*	Ring AB Torsion (°) ^c^*	Ring BC Torsion (°) ^d,e^*
**33** R = H	97.60(4)	−154.97(12)	−161.40(11)	62.36(16)	−119.43(11)
**43** R = F	90.40(6)	156.49(16)	178.40(17)	−76.6(2)	130.71(15)
**CA-4**	-	-	-	55	-
**Colchicine**	-	-	-	53	-

^a^ C18-C13-N1-C2, C26-C21-N1-C2, C26-C21-N1-C2, C14-C13-N1-C2, C14-C13-N1-C2, C19-C14-N1-C2, C14-C13-N1-C2; ^b^ C10-C5-C4-N1, C7-C6-C5-N1, C11-C6-C5-N1, C6-C5-C4-N1, C6-C5-C4-N1, C10-C5-C4-N1, C6-C5-C4-N1; ^c^ C13-N1-C4-C5, C21-N1-C5-C6, C21-N1-C5-C6, C13-N1-C4-C5, C13-N1-C4-C5, C14-N1-C4-C5, C13-N1-C4-C5; ^d^ C5-C4-C3-C26, C6-C5-C4-O14, C6-C5-C4-O14, C5-C4-C3-Cl1, C5-C4-C3-O26, C5-C4-C3-F1, C5-C4-C3-F1; ^e^ Ring C = β-lactam ring; * = 2 independent molecules in the asymmetric unit. Each angle given but only the first atom numbering scheme is outlined above.

## Data Availability

Data is contained within the article and Supplementary Materials.

## Supplementary Materials

The following supporting information can be downloaded at: https://www.mdpi.com/article/10.3390/ph15091044/s1, Table S1: Tier-1 Profiling screen of 3-fluoroazetidinones, 3,3-difluororoazetidinones and related compounds; Table S2: ADMET and Lipinski properties for 3-fluoroazetidinones, 3,3-difluoroazetidinones and related compounds; Table S3: Growth inhibition of compounds **33**, **37** and **43** in the NCI 60 cell line in vitro screen (10 μM); Table S4: Antiproliferative effects of 3-fluoroazetidinone compounds **26**, **32**, **33**, **42** and **43** in MCF-7 human breast cancer cells; Table S5: Standard COMPARE analysis of compound **33** based on one dose (10 μM). Table S6: Standard COMPARE Analysis of compound **33** based on GI_50_ mean graph; Table S7: Stability study for compounds **33** and **39**. Table S8: Docking scores for compounds **32**, **33**, **42** and **43**. Figures S1–S10: ^1^H NMR, ^13^C NMR, ^19^F NMR spectra; Figure S11: Cell viability data for 3-fluoro and 3,3-difluoro-β-lactam compounds in SW-480 cell line; Figure S12: Heat map for compound **33** (NCI 792959) and macbecin (NSC S330500); Figure S13: Protein–ligand interactions for the 3-fluoro and 3,3-difluoro-β-lactam compounds.

## Abbreviations

ADC	Antibody–drug conjugate
ATR	Attenuated total reflection
CA-4	Combretastatin A-4
DBU	1,8-Diazabicyclo[5.4.0]undec-7-ene
DCM	Dichloromethane
DMEM	Dulbecco’s modified Eagle’s medium
DMSO	Dimethylsulfoxide
ECACC	European Collection of Animal Cell Cultures
ER	Estrogen receptor
GI_50_	50% growth inhibitory concentration
HER2	Human epidermal growth factor receptor 2
IC_50_	Half-maximal inhibitory concentration
LC_50_	Median lethal concentration
MBC	Metastatic breast cancer
MRP	Multidrug resistance protein
MTA	Microtubule-targeting agent
NCI	National Cancer Institute
PBS	Phosphate-buffered saline
PBST	Phosphate-buffered saline with Tween 20
P-gp	P-glycoprotein
PI	Propidium iodide
PIK3	Phosphatidylinositol-4,5-bisphosphate 3-kinase
PR	Progesterone receptor
SRB	Sulphorhodamine B
TBAF	*tert*-Butylammonium fluoride
TBDMSCl	*tert*-Butyldimethylsilyl chloride
TGI	Total growth inhibitory concentration
THF	Tetrahydrofuran
TLC	Thin layer chromatography
TNBC	Triple-negative breast cancer

## References

[1] World Health Organization Breast Cancer. https://www.who.int/news-room/fact-sheets/detail/breast-cancer.

[2] Sung H., Ferlay J., Siegel R.L., Laversanne M., Soerjomataram I., Jemal A., Bray F. (2021). Global cancer statistics 2020: Globocan estimates of incidence and mortality worldwide for 36 cancers in 185 countries. CA Cancer J. Clin..

[3] Waks A.G., Winer E.P. (2019). Breast cancer treatment: A review. JAMA.

[4] Godone R., Leitão G., Araújo N., Castelletti C., Lima-Filho J., Martins D. (2018). Clinical and molecular aspects of breast cancer: Targets and therapies. Biomed. Pharmacother..

[5] Li X., Yang J., Peng L., Sahin A.A., Huo L., Ward K.C., O’Regan R., Torres M.A., Meisel J.L. (2017). Triple-negative breast cancer has worse overall survival and cause-specific survival than non-triple-negative breast cancer. Breast Cancer Res. Treat..

[6] DeSantis C.E., Ma J., Gaudet M.M., Newman L.A., Miller K.D., Goding Sauer A., Jemal A., Siegel R.L. (2019). Breast cancer statistics, 2019. CA Cancer J. Clin..

[7] Liao M., Zhang J., Wang G., Wang L., Liu J., Ouyang L., Liu B. (2021). Small-molecule drug discovery in triple negative breast cancer: Current situation and future directions. J. Med. Chem..

[8] Jordan M.A., Wilson L. (2004). Microtubules as a target for anticancer drugs. Nat. Rev. Cancer.

[9] Van Vuuren R.J., Visagie M.H., Theron A.E., Joubert A.M. (2015). Antimitotic drugs in the treatment of cancer. Cancer Chemother. Pharmacol..

[10] Duranti S., Fabi A., Filetti M., Falcone R., Lombardi P., Daniele G., Franceschini G., Carbognin L., Palazzo A., Garganese G. (2021). Breast cancer drug approvals issued by EMA: A review of clinical trials. Cancers.

[11] A Study of Tucatinib vs. Placebo in Combination with Capecitabine & Trastuzumab in Patients with Advanced HER2+ Breast Cancer (HER2CLIMB). https://www.clinicaltrials.gov/ct2/show/nct02614794.

[12] FDA Approves Alpelisib for Metastatic Breast Cancer. https://www.fda.gov/drugs/resources-information-approved-drugs/fda-approves-alpelisib-metastatic-breast-cancer.

[13] Andre F., Ciruelos E., Rubovszky G., Campone M., Loibl S., Rugo H.S., Iwata H., Conte P., Mayer I.A., Kaufman B. (2019). Alpelisib for PIK3CA-mutated, hormone receptor-positive advanced breast cancer. N. Engl. J. Med..

[14] FDA Approves Pembrolizumab for High-Risk Early-Stage Triple-Negative Breast Cancer. https://www.fda.gov/drugs/resources-information-approved-drugs/fda-approves-pembrolizumab-high-risk-early-stage-triple-negative-breast-cancer.

[15] Rugo H.S., Im S.A., Cardoso F., Cortes J., Curigliano G., Musolino A., Pegram M.D., Wright G.S., Saura C., Escriva-de-Romani S. (2021). Efficacy of margetuximab vs. trastuzumab in patients with pretreated ERBB2-positive advanced breast cancer: A phase 3 randomized clinical trial. JAMA Oncol..

[16] Enhertu (Trastuzumab Deruxtecan) Approved in the US for HER2-Positive Unresectable or Metastatic Breast Cancer following Two or More Prior Anti-her2 Based Regimens. https://www.astrazeneca.com/media-centre/press-releases/2019/enhertu-trastuzumab-deruxtecan-approved-in-the-us-for-her2-positive-unresectable-or-metastatic-breast-cancer-following-2-or-more-prior-anti-her2-based-regimens.html.

[17] Spring L.M., Nakajima E., Hutchinson J., Viscosi E., Blouin G., Weekes C., Rugo H., Moy B., Bardia A. (2021). Sacituzumab govitecan for metastatic triple-negative breast cancer: Clinical overview and management of potential toxicities. Oncologist.

[18] McGuinness J.E., Kalinsky K. (2021). Antibody-drug conjugates in metastatic triple negative breast cancer: A spotlight on sacituzumab govitecan, ladiratuzumab vedotin, and trastuzumab deruxtecan. Expert Opin. Biol. Ther..

[19] Ravelli R.B., Gigant B., Curmi P.A., Jourdain I., Lachkar S., Sobel A., Knossow M. (2004). Insight into tubulin regulation from a complex with colchicine and a stathmin-like domain. Nature.

[20] Lu Y., Chen J., Xiao M., Li W., Miller D.D. (2012). An overview of tubulin inhibitors that interact with the colchicine binding site. Pharm. Res..

[21] Bukhari S.N.A., Kumar G.B., Revankar H.M., Qin H.L. (2017). Development of combretastatins as potent tubulin polymerization inhibitors. Bioorg. Chem..

[22] Karatoprak G.S., Kupeli Akkol E., Genc Y., Bardakci H., Yucel C., Sobarzo-Sanchez E. (2020). Combretastatins: An overview of structure, probable mechanisms of action and potential applications. Molecules.

[23] Greene L.M., Meegan M.J., Zisterer D.M. (2015). Combretastatins: More than just vascular targeting agents?. J. Pharmacol. Exp. Ther..

[24] Grisham R., Ky B., Tewari K.S., Chaplin D.J., Walker J. (2018). Clinical trial experience with CA4P anticancer therapy: Focus on efficacy, cardiovascular adverse events, and hypertension management. Gynecol. Oncol. Res. Pract..

[25] Garon E.B., Neidhart J.D., Gabrail N.Y., de Oliveira M.R., Balkissoon J., Kabbinavar F. (2016). A randomized phase II trial of the tumor vascular disrupting agent CA4P (fosbretabulin tromethamine) with carboplatin, paclitaxel, and bevacizumab in advanced nonsquamous non-small-cell lung cancer. OncoTargets Ther..

[26] Blay J.Y., Papai Z., Tolcher A.W., Italiano A., Cupissol D., Lopez-Pousa A., Chawla S.P., Bompas E., Babovic N., Penel N. (2015). Ombrabulin plus cisplatin versus placebo plus cisplatin in patients with advanced soft-tissue sarcomas after failure of anthracycline and ifosfamide chemotherapy: A randomised, double-blind, placebo-controlled, phase 3 trial. Lancet Oncol..

[27] Bates D., Eastman A. (2017). Microtubule destabilising agents: Far more than just antimitotic anticancer drugs. Br. J. Clin. Pharmacol..

[28] Wu X., Wang Q., Li W. (2016). Recent advances in heterocyclic tubulin inhibitors targeting the colchicine binding site. Anti-Cancer Agents Med. Chem..

[29] McLoughlin E.C., O’Boyle N.M. (2020). Colchicine-binding site inhibitors from chemistry to clinic: A review. Pharmaceuticals.

[30] Sherbet G.V. (2020). Combretastatin analogues in cancer biology: A prospective view. J. Cell. Biochem..

[31] O’Boyle N.M., Carr M., Greene L.M., Bergin O., Nathwani S.M., McCabe T., Lloyd D.G., Zisterer D.M., Meegan M.J. (2010). Synthesis and evaluation of azetidinone analogues of combretastatin A-4 as tubulin targeting agents. J. Med. Chem..

[32] Wang S., Malebari A.M., Greene T.F., O’Boyle N.M., Fayne D., Nathwani S.M., Twamley B., McCabe T., Keely N.O., Zisterer D.M. (2019). 3-vinylazetidin-2-ones: Synthesis, antiproliferative and tubulin destabilizing activity in MCF-7 and MDA-MB-231 breast cancer cells. Pharmaceuticals.

[33] Malebari A.M., Fayne D., Nathwani S.M., O’Connell F., Noorani S., Twamley B., O’Boyle N.M., O’Sullivan J., Zisterer D.M., Meegan M.J. (2020). β-lactams with antiproliferative and antiapoptotic activity in breast and chemoresistant colon cancer cells. Eur. J. Med. Chem..

[34] Malebari A.M., Greene L.M., Nathwani S.M., Fayne D., O’Boyle N.M., Wang S., Twamley B., Zisterer D.M., Meegan M.J. (2017). β-lactam analogues of combretastatin A-4 prevent metabolic inactivation by glucuronidation in chemoresistant HT-29 colon cancer cells. Eur. J. Med. Chem..

[35] Arya N., Jagdale A.Y., Patil T.A., Yeramwar S.S., Holikatti S.S., Dwivedi J., Shishoo C.J., Jain K.S. (2014). The chemistry and biological potential of azetidin-2-ones. Eur. J. Med. Chem..

[36] Nagpal R., Bhalla J., Bari S.S. (2019). A comprehensive review on C-3 functionalization of β-lactams. Curr. Org. Synth..

[37] Tang H., Cheng J., Liang Y., Wang Y. (2020). Discovery of a chiral fluorinated azetidin-2-one as a tubulin polymerisation inhibitor with potent antitumour efficacy. Eur. J. Med. Chem..

[38] Zhang X., Jia Y. (2020). Recent advances in β-lactam derivatives as potential anticancer agents. Curr. Top. Med. Chem..

[39] Visconti R., Grieco D. (2017). Fighting tubulin-targeting anticancer drug toxicity and resistance. Endocr. Relat. Cancer.

[40] Gutman H., Bazylevich A., Prasad C., Dorfman O., Hesin A., Marks V., Patsenker L., Gellerman G. (2021). Discovery of dolastatinol: A synthetic analog of dolastatin 10 and low nanomolar inhibitor of tubulin polymerization. ACS Med. Chem. Lett..

[41] Banerjee S., Mahmud F., Deng S., Ma L., Yun M.K., Fakayode S.O., Arnst K.E., Yang L., Chen H., Wu Z. (2021). X-ray crystallography-guided design, antitumor efficacy, and QSAR analysis of metabolically stable cyclopenta-pyrimidinyl dihydroquinoxalinone as a potent tubulin polymerization inhibitor. J. Med. Chem..

[42] Deng S., Krutilina R.I., Wang Q., Lin Z., Parke D.N., Playa H.C., Chen H., Miller D.D., Seagroves T.N., Li W. (2020). An orally available tubulin inhibitor, VERU-111, suppresses triple-negative breast cancer tumor growth and metastasis and bypasses taxane resistance. Mol. Cancer Ther..

[43] Wang Q., Arnst K.E., Wang Y., Kumar G., Ma D., White S.W., Miller D.D., Li W., Li W. (2019). Structure-guided design, synthesis, and biological evaluation of (2-(1H-indol-3-yl)-1H-imidazol-4-yl)(3,4,5-trimethoxyphenyl) methanone (ABI-231) analogues targeting the colchicine binding site in tubulin. J. Med. Chem..

[44] Sabizabulin for COVID-19. https://verupharma.com/pipeline/veru-111-for-covid-19/.

[45] Arnst K.E., Wang Y., Hwang D.J., Xue Y., Costello T., Hamilton D., Chen Q., Yang J., Park F., Dalton J.T. (2018). A potent, metabolically stable tubulin inhibitor targets the colchicine binding site and overcomes taxane resistance. Cancer Res..

[46] (2021). A phase I/II Trial of Crolibulin (EPC2407) Plus Cisplatin in Adults with Solid Tumors with a Focus on Anaplastic Thyroid Cancer (ATC). https://clinicaltrials.gov/ct2/show/nct01240590.

[47] Wang Y., Zhang H., Gigant B., Yu Y., Wu Y., Chen X., Lai Q., Yang Z., Chen Q., Yang J. (2016). Structures of a diverse set of colchicine binding site inhibitors in complex with tubulin provide a rationale for drug discovery. FEBS J..

[48] Bohnacker T., Prota A.E., Beaufils F., Burke J.E., Melone A., Inglis A.J., Rageot D., Sele A.M., Cmiljanovic V., Cmiljanovic N. (2017). Deconvolution of buparlisib’s mechanism of action defines specific PI3K and tubulin inhibitors for therapeutic intervention. Nat. Commun..

[49] Arnst K.E., Banerjee S., Chen H., Deng S., Hwang D.J., Li W., Miller D.D. (2019). Current advances of tubulin inhibitors as dual acting small molecules for cancer therapy. Med. Res. Rev..

[50] Wang L., Zheng Y., Li D., Yang J., Lei L., Yan W., Zheng W., Tang M., Shi M., Zhang R. (2021). Design, synthesis, and bioactivity evaluation of dual-target inhibitors of tubulin and SRC kinase guided by crystal structure. J. Med. Chem..

[51] Yang L., Zhang W., Qiu Q., Su Z., Tang M., Bai P., Si W., Zhu Z., Liu Y., Yang J. (2021). Discovery of a series of hydroxamic acid-based microtubule destabilizing agents with potent antitumor activity. J. Med. Chem..

[52] Wang K., Zhong H., Li N., Yu N., Wang Y., Chen L., Sun J. (2021). Discovery of novel anti-breast-cancer inhibitors by synergistically antagonizing microtubule polymerization and aryl hydrocarbon receptor expression. J. Med. Chem..

[53] Zheng L., Ren R., Sun X., Zou Y., Shi Y., Di B., Niu M.M. (2021). Discovery of a dual tubulin and poly(adp-ribose) polymerase-1 inhibitor by structure-based pharmacophore modeling, virtual screening, molecular docking, and biological evaluation. J. Med. Chem..

[54] Meanwell N.A. (2018). Fluorine and fluorinated motifs in the design and application of bioisosteres for drug design. J. Med. Chem..

[55] Inoue M., Sumii Y., Shibata N. (2020). Contribution of organofluorine compounds to pharmaceuticals. ACS Omega.

[56] Alloatti D., Giannini G., Cabri W., Lustrati I., Marzi M., Ciacci A., Gallo G., Tinti M.O., Marcellini M., Riccioni T. (2008). Synthesis and biological activity of fluorinated combretastatin analogues. J. Med. Chem..

[57] Carr M., Greene L.M., Knox A.J., Lloyd D.G., Zisterer D.M., Meegan M.J. (2010). Lead identification of conformationally restricted β-lactam type combretastatin analogues: Synthesis, antiproliferative activity and tubulin targeting effects. Eur. J. Med. Chem..

[58] Hosseyni S., Jarrahpour A. (2018). Recent advances in β-lactam synthesis. Org. Biomol. Chem..

[59] Leite T.H.O., Saraiva M.F., Pinheiro A.C., de Souza M.V.N. (2020). Monocyclic β-lactam: A review on synthesis and potential biological activities of a multitarget core. Mini Rev. Med. Chem..

[60] Pitts C.R., Lectka T. (2014). Chemical synthesis of β-lactams: Asymmetric catalysis and other recent advances. Chem. Rev..

[61] Deketelaere S., Van Nguyen T., Stevens C.V., D’Hooghe M. (2017). Synthetic approaches toward monocyclic 3-amino-β-lactams. ChemistryOpen.

[62] Georg G.I., Cheruvallath Z.S., Himes R.H., Mejillano M.R., Burke C.T. (1992). Synthesis of biologically active taxol analogues with modified phenylisoserine side chains. J. Med. Chem..

[63] Tarui A. (2015). Stereoselective synthesis of multi-substituted fluoro-β-lactams and their conversion to fluorinated β-amino acid core. Yakugaku Zasshi.

[64] Tarui A. (2020). Stereoselective synthesis of multisubstituted α-fluoro-β-lactams. Curr. Org. Chem..

[65] Tantawy A.H., El-Behairy M.F., Abd-Allah W.H., Jiang H., Wang M.Q., Marzouk A.A. (2021). Design, synthesis, biological evaluation, and computational studies of novel fluorinated candidates as PI3Kinhibitors: Targeting fluorophilic binding sites. J. Med. Chem..

[66] Lara-Ochoa F., Espinosa-Pérez G. (2007). A new synthesis of combretastatins A-4 and AVE-8062A. Tetrahedron Lett..

[67] Combes S., Barbier P., Douillard S., McLeer-Florin A., Bourgarel-Rey V., Pierson J.T., Fedorov A.Y., Finet J.P., Boutonnat J., Peyrot V. (2011). Synthesis and biological evaluation of 4-arylcoumarin analogues of combretastatins. Part 2. J. Med. Chem..

[68] Vaske Y.S., Mahoney M.E., Konopelski J.P., Rogow D.L., McDonald W.J. (2010). Enantiomerically pure trans-β-lactams from α-amino acids via compact fluorescent light (CFL) continuous-flow photolysis. J. Am. Chem. Soc..

[69] Twamley B., O’Boyle N.M., Meegan M.J. (2020). Azetidin-2-ones: Structures of anti-mitotic compounds based on the 1-(3,4,5-tri-meth-oxy-phen-yl)azetidin-2-one core. Acta Crystallogr. E Crystallogr. Commun..

[70] Zajac M., Jelinska A., Cielecka-Piontek J., Oszczapowicz I. (2005). Stability of aztreonam in azactam. Farmaco.

[71] Singh S., Singh B., Bahuguna R., Wadhwa L., Saxena R. (2006). Stress degradation studies on ezetimibe and development of a validated stability-indicating HPLC assay. J. Pharm. Biomed. Anal..

[72] Pipeline Pilot Overview. https://www.3ds.com/fileadmin/products-services/biovia/pdf/biovia-pipeline%20pilot-pipeline-pilot-overview.pdf.

[73] O’Boyle N.M., Greene L.M., Keely N.O., Wang S., Cotter T.S., Zisterer D.M., Meegan M.J. (2013). Synthesis and biochemical activities of antiproliferative amino acid and phosphate derivatives of microtubule-disrupting β-lactam combretastatins. Eur. J. Med. Chem..

[74] Baell J.B., Nissink J.W.M. (2018). Seven year itch: Pan-assay interference compounds (PAINS) in 2017-utility and limitations. ACS Chem. Biol..

[75] Davis A.W., Ward S.E. (2014). The Handbook of Medicinal Chemistry: Principles and Practice.

[76] Cushman M., Nagarathnam D., Gopal D., He H.M., Lin C.M., Hamel E. (1992). Synthesis and evaluation of analogues of (z)-1-(4-methoxyphenyl)-2-(3,4,5-trimethoxyphenyl)ethene as potential cytotoxic and antimitotic agents. J. Med. Chem..

[77] Ma M., Sun L., Lou H., Ji M. (2013). Synthesis and biological evaluation of combretastatin a-4 derivatives containing a 3′-O-substituted carbonic ether moiety as potential antitumor agents. Chem. Cent. J..

[78] Messaoudi S., Treguier B., Hamze A., Provot O., Peyrat J.F., De Losada J.R., Liu J.M., Bignon J., Wdzieczak-Bakala J., Thoret S. (2009). Isocombretastatins a versus combretastatins a: The forgotten isoCA-4 isomer as a highly promising cytotoxic and antitubulin agent. J. Med. Chem..

[79] Hughes L., Malone C., Chumsri S., Burger A.M., McDonnell S. (2008). Characterisation of breast cancer cell lines and establishment of a novel isogenic subclone to study migration, invasion and tumourigenicity. Clin. Exp. Metastasis.

[80] National Cancer Institute Biological Testing Branch; Developmental Therapeutics Program; National Cancer Institute: Bethesda, MD, USA. https://dtp.cancer.gov.

[81] Lee S.Y. (2016). Temozolomide resistance in glioblastoma multiforme. Genes Dis..

[82] Compare Analysis. https://dtp.cancer.gov/databases_tools/compare.htm.

[83] Bates S.E., Fojo A.T., Weinstein J.N., Myers T.G., Alvarez M., Pauli K.D., Chabner B.A. (1995). Molecular targets in the National Cancer Institute Drug Screen. J. Cancer Res. Clin. Oncol..

[84] Shoemaker R.H. (2006). The NCI60 human tumour cell line anticancer drug screen. Nat. Rev. Cancer.

[85] Pena-Blanco A., Garcia-Saez A.J. (2018). Bax, Bak and beyond—Mitochondrial performance in apoptosis. FEBS J..

[86] Roberts A.W. (2020). Therapeutic development and current uses of Bcl-2 inhibition. Hematol. Am. Soc. Hematol. Educ. Program.

[87] Kale J., Osterlund E.J., Andrews D.W. (2018). Bcl-2 family proteins: Changing partners in the dance towards death. Cell Death Differ..

[88] Mori A., Wada H., Nishimura Y., Okamoto T., Takemoto Y., Kakishita E. (2002). Expression of the antiapoptosis gene survivin in human leukemia. Int. J. Hematol..

[89] Jha K., Shukla M., Pandey M. (2012). Survivin expression and targeting in breast cancer. Surg. Oncol..

[90] Jaiswal P.K., Goel A., Mittal R.D. (2015). Survivin: A molecular biomarker in cancer. Indian J. Med. Res..

[91] Li F., Aljahdali I., Ling X. (2019). Cancer therapeutics using survivin BIRC5 as a target: What can we do after over two decades of study?. J. Exp. Clin. Cancer Res..

[92] Hawkins P.C., Skillman A.G., Warren G.L., Ellingson B.A., Stahl M.T. (2010). Conformer generation with omega: Algorithm and validation using high quality structures from the protein databank and Cambridge Structural Database. J. Chem. Inf. Model..

[93] Openeye Scientific Software, Inc. Omega 4.1.0. 2. http://www.eyesopen.com.

[94] McGann M. (2011). FRED Pose prediction and virtual screening accuracy. J. Chem. Inf. Model..

[95] Wienecke A., Bacher G. (2009). Indibulin, a novel microtubule inhibitor, discriminates between mature neuronal and nonneuronal tubulin. Cancer Res..

[96] Bruker AXC Inc. (2012). Bruker APEX 2 v2012.12-0.

[97] Sheldrick G.M. (2014). SADABS.

[98] Sheldrick G.M. (2015). SHELXT-integrated space-group and crystal-structure determination. Acta Crystallogr. A Found. Adv..

[99] Sheldrick G.M. (2008). A short history of SHELX. Acta Crystallogr. A.

[100] Dolomanov O.V., Bourhis L.J., Gildea R.J., Howard J.A.K., Puschmann H. (2009). Olex2: A complete structure solution, refinement and analysis program. J. Appl. Crystallogr..

