# Supplementary material for: Synthesis, Characterisation and Mechanism of Action of Anticancer 3-Fluoroazetidin-2-ones
Source: Pharmaceuticals (Basel). 2022 Aug 24;15(9):1044. doi: 10.3390/ph15091044 (PMC9501633; doi:10.3390/ph15091044)
Supplement: Supplementary file 1 [file pharmaceuticals-15-01044-s001.zip › pharmaceuticals-1860452-supplementary.pdf]

Supplementary Information

Synthesis, characterization and mechanism of action of anticancer 3-fluoroazetidin-2-ones

Azizah M. Malebari[a,b*], Gabriela Duffy Morales[b], Brendan Twamley[c], Darren Fayne[d], Mohemmed Faraz Khan[d], Eavan C. McLoughlin[b], Niamh M. O'Boyle[b], Daniela M. Zisterer[d] and Mary J. Meegan[b*]

[a] Department of Pharmaceutical Chemistry, College of Pharmacy, King Abdulaziz University, Jeddah, Saudi Arabia

[b] School of Pharmacy and Pharmaceutical Sciences, Trinity College Dublin, Trinity Biomedical Sciences Institute, 152-160 Pearse Street, Dublin 2, Ireland

[c] School of Chemistry, Trinity College Dublin, Dublin 2, Ireland

[d] School of Biochemistry and Immunology, Trinity College Dublin, Trinity Biomedical Sciences Institute, 152-160 Pearse Street, Dublin 2, Ireland

Experimental chemistry: Preparation of [3-(*tert*-Butyldimethylsilanyloxy)-4-methoxybenzylidene](3,4,5-trimethoxyphenyl)amine **22**

Table S1: Tier-1 Profiling Screen of selected 3-fluoroazetidinones, 3,3-difluoroazetidinones and related compounds

Table S2: ADMET and Lipinski Properties for selected 3-fluoroazetidinones, 3,3-difluoroazetidin-2-ones and related compounds

Table S3: Growth inhibition of compounds **33**, **37** and **43** in the NCI60 cell line *in vitro* screen (10 μM)

Table S4. Antiproliferative effects of β-lactam compounds **26, 32, 33, 42** and **43** in MCF-7 human breast cancer cells

Table S5: Standard COMPARE Analysis of compound **33** based on one-dose (10 μM)

Table S6: Standard COMPARE Analysis of compounds **33** based on GI_{50} mean graph

Table S7: Stability study for compounds **33** and **39**

Table S8: Docking Scores for compounds **32**, **33**, **42** and **43**

Figure S1: ^{1}H NMR spectrum of compound **28**

Figure S2: ^{13}C NMR spectrum of compound **28**

Figure S3: ^{1}H NMR spectrum of compound **37**

Figure S4: ^{13}C NMR spectrum of compound **37**

Figure S5: ^{1}H-NMR spectrum of compound **33**

Figure S6: ^{13}C-NMR spectrum of compound **33**

Figure S7: ^{1}H-NMR spectrum of compound **39**

Figure S8: ^{1}H-NMR spectrum of compound **41**

Figure S9: ^{1}H-NMR spectrum of compound **42**

Figure S10: ^{19}F NMR spectrum of compound **23**

Figure S11: Cell viability data for 3-fluoro and 3,3-difluoro-β-lactam compounds in SW-480 cell line

Figure S12: Heat map compound 792959 and macbeccin NSC S330500

Figure S13: Protein-ligand interactions for the 3-fluoro and 3,3-difluoro-β-lactam compounds **26** and **36**.

References

Experimental chemistry:

[3-(*tert*-Butyldimethylsilanyloxy)-4-methoxybenzylidene](3,4,5-trimethoxyphenyl)amine 22

DBU (8 mmol) was added dropwise to a stirred solution of imine **22** (5 mmol) and *tert*-butyldimethylsilyl chloride (6 mmol) in anhydrous CH_2Cl_2 (40 mL) under a nitrogen atmosphere (reaction was continued for 2-4 h until complete as indicated by TLC (eluent, 1:1 hexane/ethyl acetate)). The reaction mixture was diluted with CH_2Cl_2 (50 mL) and washed with water (100 mL), 0.1 M HCl (50 mL) and saturated aqueous $NaHCO_3$ (50 mL), before drying over anhydrous $NaSO_4$. The solvent was removed *in vacuo* to yield TBDMS-protected imine **22** as amber oil, (yield 52%), purity (HPLC): 100%. Imine **22** was characterised as described previously by us [1], [2]. IR ν_{max} (KBr) cm^1:1618.8, (C=N). ^1H NMR (400 MHz, DMSO-d6): δ 0.21 (s, 6H, $Si(CH_3)_2$, 1.04 (s, 9H, $SiC(CH_3)_3$), 3.74 (s, 3H, OCH_3), 3.87 (s, 6H, $2xOCH_3$), 3.93 (s, 3H, OCH_3), 6.58 (s, 2H, Ar-H), 7.12 (d, 1H, J = 8.2 Hz, Ar-H), 7.48-7.55 (m, 2H, Ar-H), 8.51 (s, 1H, CH=N). ^{13}C NMR (100 MHz, DMSO-d6): δ 5.78 $Si(CH_3)_2$,17.71 $SiC(CH_3)_3$), 24.74 (C $(CH_3)_3$), 55.00 (OCH_3), 59.25 (OCH_3), 98.04, 111.22, 121.27, 125.43, 129.50, 135.95, 144.68, 147.73, 153.32 (Ar-C), 158.27 (CH=N). HRMS: Calculated for $C_{23}H_{34}O_5Si$: 432.2216; Found: 432.2213 $(M+ H)^+$.

Table S1: Tier-1 Profiling Screen of selected 3-fluoroazetidin-2-ones, 3,3-difluoroazetidin-2-ones[a]

Compound	Number	ADMET Solubility[b]	ADMET Solubility Level[c]	ADMET_ BBB[d]	ADMET BBB Level[e]	ADMET CYP2D6 Prediction[f]	ADMET Hepatotoxic Prediction[g]
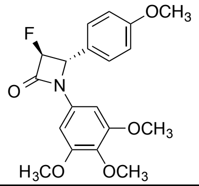	**26**	-4.1380	2	-0.14200	2	false	true
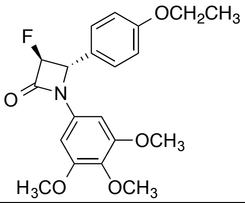	**27**	-4.3290	2	-0.034000	2	false	false
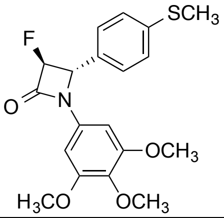	**28**	-4.6260	2	0.17200	1	false	true
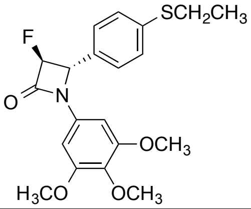	**29**	-4.8160	2	0.28000	1	false	true
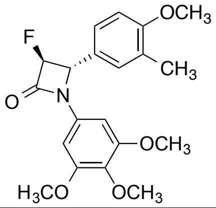	**30**	-4.6190	2	0.0080000	1	false	true
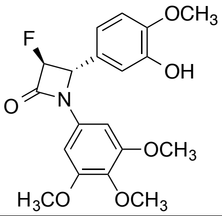	**32**	-3.8020	3	-0.54600	3	false	true
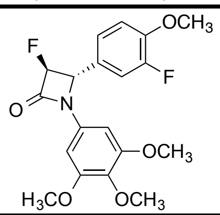	**33**	-4.3740	2	-0.079000	2	false	true

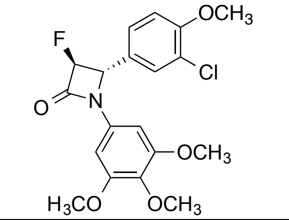	**34**	-4.7750	2	0.063000	1	false	true
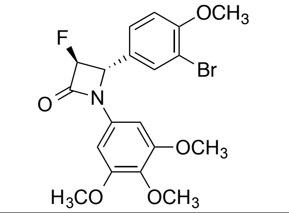	**35**	-4.8480	2	0.089000	1	false	true
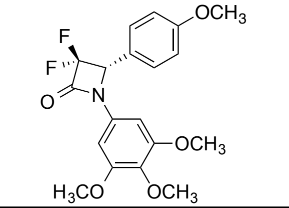	**36**	-4.3990	2	-0.090000	2	false	true
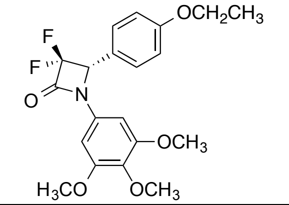	**37**	-4.5850	2	0.018000	1	false	false
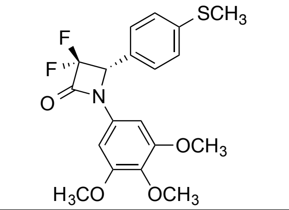	**38**	-4.8860	2	0.22400	1	false	true
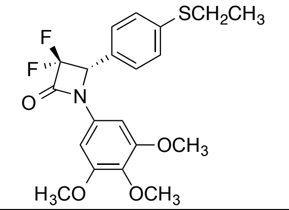	**39**	-5.0730	2	0.33200	1	false	true
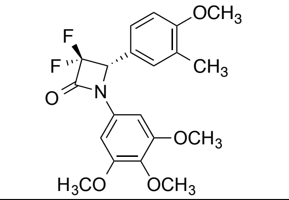	**40**	-4.8770	2	0.060000	1	false	true
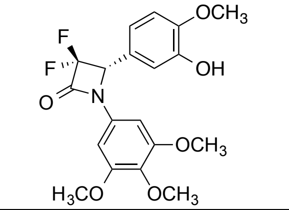	**42**	-4.1460	2	-0.49400	2	false	true

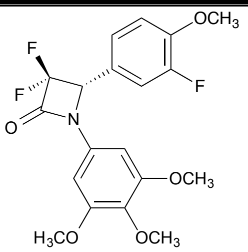	**43**	-4.6320	2	-0.026000	2	false	true
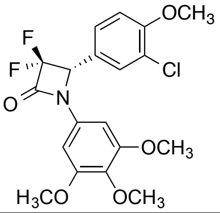	**44**	-5.0330	2	0.11500	1	false	true
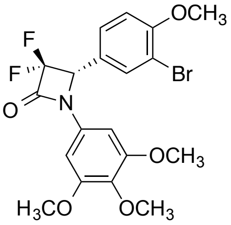	**45**	-5.1060	2	0.14100	1	false	true

[a]Calculated using Pipeline Pilot Professional (v8.5.0.200) BIOVIA, Dassault Systèmes
[b]ADMET Solubility: Log of the water solubility at 25 °C (LogSw)(mol/L)
[c]ADMET Solubility Level: Ranking of the solubility values into the following classes: 0: Extremely Low; 1: Very Low; 2: Low; 3: Good; 4: Optimal; 5: Very Soluble
[d]ADMET BBB: Predicts the blood brain barrier penetration of a molecule, defined as the ratio of the concentrations of solute (compound) on the both sides of the membrane after oral administration.
[e]ADMET Blood Brain Barrier Absorption (BBB) Level: Ranking of LogBBB values into one of the following levels: 0: Very High; 1: High; 2: Medium; 3: Low; 4: Undefined (molecule is outside the confidence area of the regression model used to calculate LogBB)
[f]CYP2D6 inhibitor prediction
[g]Human hepatoxicity prediction

Table S2: ADMET and Lipinski Properties for Selected 3-fluoroazetidin-2-ones, 3,3-difluoroazetidin-2-ones[a]

Compound	No.	ADMET Absorption Level[b]	ADMET PPB Prediction[c]	ALogP[d]	MW	Num HBA	Num HBD	Num Rot Bonds	Molecular Volume	Molecular Polar Surface Area
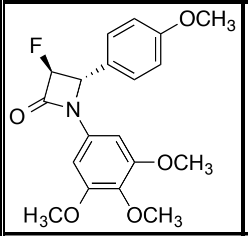	**26**	0	true	2.9240	361.36	5	0	6	127.59	57.230
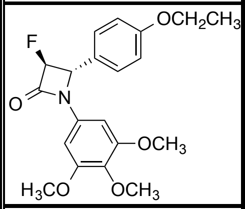	**27**	0	true	3.2730	375.39	5	0	7	133.42	57.230
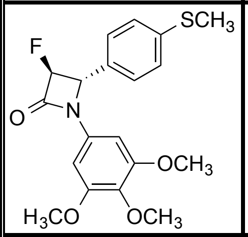	**28**	0	true	3.4830	377.43	5	0	6	130.68	73.300
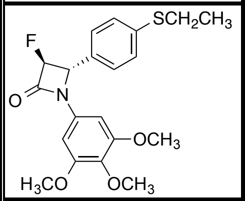	**29**	0	true	3.8310	391.46	5	0	7	136.85	73.300
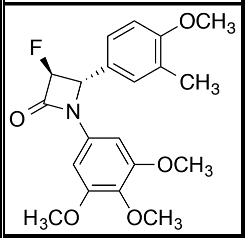	**30**	0	true	3.4110	375.39	5	0	6	130.68	57.230
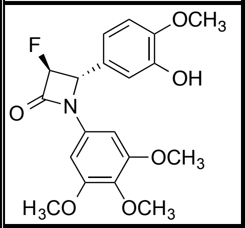	**32**	0	true	2.6820	377.36	6	1	6	128.96	77.460
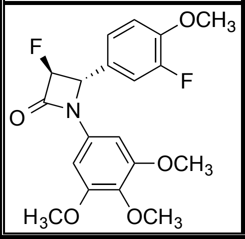	**33**	0	true	3.1300	379.35	5	0	6	128.28	57.230

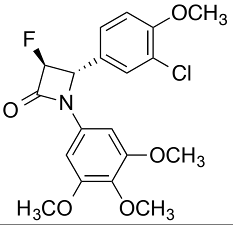	**34**	0	true	3.5890	395.81	5	0	6	130.33	57.230
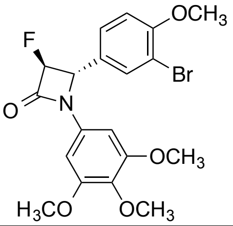	**35**	0	true	3.6730	440.26	5	0	6	141.65	57.230
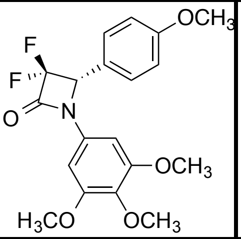	**36**	0	true	3.0930	379.35	5	0	6	128.96	57.230
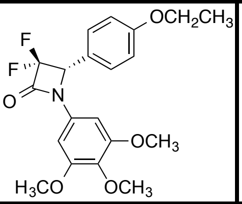	**37**	0	true	3.4420	393.38	5	0	7	134.79	57.230
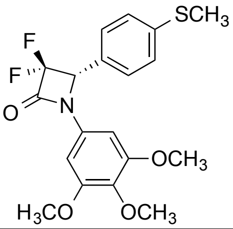	**38**	0	true	3.6520	395.42	5	0	6	131.71	73.300
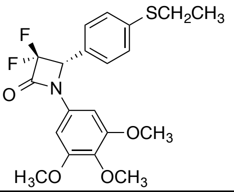	**39**	0	true	4.00	409.45	5	0	7	137.88	73.300
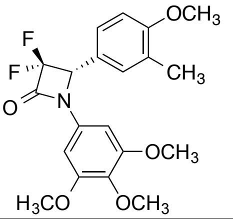	**40**	0	true	3.5800	393.38	5	0	6	132.05	57.230
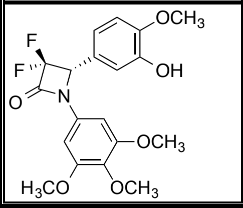	**42**	0	true	2.8510	395.35	6	1	6	130.33	77.460

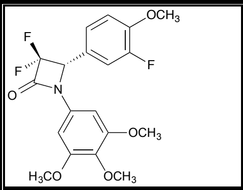	**43**	0	true	3.2990	397.35	5	0	6	128.62	57.230
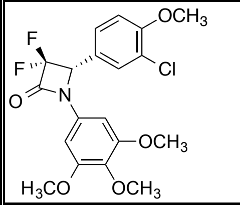	**44**	0	true	3.7580	413.80	5	0	6	131.71	57.230
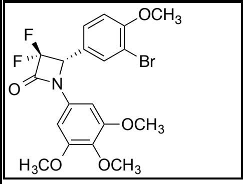	**45**	0	true	3.8420	458.25	5	0	6	142.34	57.230

[a]Calculated using Pipeline Pilot Professional (v8.5.0.200) BIOVIA, Dassault Systèmes
[b]ADMET Calculates ADMET Passive Intestinal Absorption properties. Accelrys passive intestinal absorption model. Absorption Level: Ranking of the molecule into one of the following levels: 0: Good; 1: Moderate; 2: Poor; 3: Very Poor
[c]ADMET Plasma Protein Binding (PPB) Prediction: If true, the compound is predicted to be a binder (>=90%). Otherwise, it is predicted to be a weak or nonbinder (<90%).
[d] ChemBioDraw Ultra 13.0.2.3020

Table S3: Growth inhibition of compounds **33** (NCI 792959), **37** (NCI 792958) and **43** NCI 792960) in the NCI60 cell line *in vitro* screen (10 μM)[a]

	Compound **33**		Compound **37**		Compound **43**	
	Growth %	Inhibition %	Growth %	Inhibition %	Growth %	Inhibition %
**Leukaemia**						
CCRF-CEM	13.16	86.84	10.2	89.8	12.96	87.04
HL-60(TB)	-14.69	114.69	-15.97	115.97	0.94	99.06
K-562	9.45	90.55	8.49	91.51	10.5	89.5
MOLT-4	19.7	80.3	18.14	81.86	18.67	81.33
RPMI-8226	15.32	84.68	14.64	85.36	20.78	79.22
SR	13.28	86.72	16.25	83.75	18.7	81.3
**Non-Small Cell Lung Cancer**						
A549/A TCC	15.33	84.67	33.56	66.44	48.65	51.35
EKVX	72.16	27.84	75.34	24.66	77.7	22.3
HOP-62	31.85	68.15	40.81	59.19	38.43	61.57
HOP-92	32.28	67.72	55.62	44.38	60.84	39.16
NCI-H226	58.99	41.01	71.01	28.99	75.53	24.47
NCI-H23	21.3	78.7	33.57	66.43	37.86	62.14
NCI-H322M	46.45	53.55	67.23	32.77	91.21	8.79
NCI-H460	7.06	92.94	12.73	87.27	27.15	72.85
NCI-H522	-8.46	108.46	19.28	80.72	17.54	82.46
**Colon Cancer**						
COLO 205	-68	168	10.78	89.22	16.32	83.68
HCC-2998	32.93	67.07	81.66	18.34	84.2	15.8
HCT-116	5.59	94.41	8.92	91.08	5.76	94.24
HCT-15	13.63	86.37	22.55	77.45	19.44	80.56
HT29	3.73	96.27	12.27	87.73	11.16	88.84
KM12	10.73	89.27	27.97	72.03	22.9	77.1
SW-620	28.73	71.27	25.47	74.53	21.22	78.78
**CNS Cancer**						
SF-268	33.13	66.87	52.38	47.62	52.94	47.06
SF-295	18.61	81.39	56.32	43.68	48.29	51.71
SF-539	4.17	95.83	10.75	89.25	21.65	78.35
SNB-19	34.05	65.95	54.14	45.86	52.93	47.07
SNB-75	50.89	49.11	55.93	44.07	64.74	35.26
U251	11.86	88.14	36.19	63.81	41.61	58.39

	Compound **33**		Compound **37**		Compound **43**	
	Growth %	Inhibition %	Growth %	Inhibition %	Growth %	Inhibition %
**Melanoma**						
LOX IMVI	23.98	76.02	27.57	72.43	27.06	72.94
MALME-3M	74.02	25.98	63.07	36.93	64.09	35.91
M14	1.91	98.09	27.45	72.55	24.24	75.76
MDA-MB-435	0.35	99.65	16.83	83.17	12.92	87.08
SK-MEL-2	58.83	41.17	91.78	8.22	84.61	15.39
SK-MEL-28	57.74	42.26	66.8	33.2	77.22	22.78
SK-MEL-5	-31.46	131.46	6.76	93.24	8.08	91.92
UACC-257	38.74	61.26	58.69	41.31	50.01	49.99
UACC-62	53.45	46.55	51.61	48.39	55.46	44.54
**Ovarian Cancer**						
IGROV1	31.88	68.12	37.8	62.2	44.2	55.8
OVCAR-3	-4.18	104.18	7.25	92.75	11.64	88.36
OVCAR-4	54.92	45.08	49.59	50.41	58.34	41.66
OVCAR-5	43.57	56.43	66.47	33.53	78.72	21.28
OVCAR-8	13.71	86.29	43.23	56.77	44.07	55.93
NCI/ADR-RES	8.4	91.6	28.94	71.06	32.94	67.06
SK-OV-3	33.36	66.64	71.89	28.11	75.49	24.51
**Renal Cancer**						
786-0	22.92	77.08	64.68	35.32	75.35	24.65
A498	-5.5	105.5	92.3	7.7	97.81	2.19
ACHN	32.67	67.33	55.25	44.75	60.82	39.18
RXF 393	28.8	71.2	54.19	45.81	49.36	50.64
SN12C	33.29	66.71	50.97	49.03	54.92	45.08
TK-10	64.4	35.6	103.32	-3.32	105.64	-5.64
UO-31	43.2	56.8	51.9	48.1	53.48	46.52
**Prostate Cancer**						
PC-3	23.13	76.87	37.3	62.7	35.89	64.11
DU-145	9.46	90.54	30.05	69.95	25.22	74.78
**Breast Cancer**						
MCF7	12.83	87.17	17.19	82.81	15.06	84.94
MDA-MB-231/ATCC	19.65	80.35	46.12	53.88	54.02	45.98
HS 578T	25.54	74.46	37.42	62.58	21.93	78.07
BT-549	15.26	84.74	44.17	55.83	34.99	65.01
T-47D	74.94	25.06	70.03	29.97	65.69	34.31
MDA-MB-468	23	77	34.89	65.11	44.48	55.52
Mean	23.66	76.34	41.05	58.95	43.57	56.43

[a]Data obtained from NCI *in vitro* human tumour cell screen 1 dose assay (10 μM). **33** (NCI 792959), **37** (NCI 792958) and **43** NCI 792960).

[National Cancer Institute Biological Testing Branch; National Cancer Institute; Bethesda, MD; https://dtp.cancer.gov (accessed 02/03/2022)].

Table S4. Antiproliferative effects of β-lactam compounds **26, 32, 33, 42** and **43** in MCF-7 human breast cancer cells

**Compound**	**Antiproliferative activity[a,b]** **MCF-7 Cells** **IC_{50} (μM)**	**Log P[c]**
**26**	0.312± 0.08	2.92
**32**	0.075± 0.002	2.68
**33**	0.095± 0.004	3.10
**42**	0.321± 0.06	2.85
**43**	1.650±0.30	3.29
**CA-4[b]**	0.0035±0.0002	3.32

[a] IC_{50} values are half maximal inhibitory concentrations required to inhibit the growth of MCF-7 breast cancer cells. Values represent the mean (SEM (error values 10^{-6}) for three experiments performed in triplicate. [b] The IC_{50} value obtained for CA-4 in this assay is in agreement with the reported values for CA-4 in MCF-7 and MDA-MB-231 human breast cancer cell lines .[3],[4]. [c]Pipeline Pilot Professional.

Table S5: Standard COMPARE analysis of compound **33** based on one-dose (10 µM)[a]

Rank	Compound	*r*
1	macbecin II	0.53
2	vincristine sulfate	0.50
3	Floxuridine	0.50
4	rhizoxin	0.49
5	tiazofurin	0.49
6	maytansine	0.48

[a]The target set was the NCI Standard Database and the target set endpoints were selected to be equal to the seed end points. Standard COMPARE analysis was performed. Correlation values (r) are Pearson correlation coefficients. [Reference National Cancer Institute Biological Testing Branch; National Cancer Institute; Bethesda, MD; https://dtp.cancer.gov (accessed 02/03/2022)].

The NSC compound reference number 792959 for the seed compound **33** was submitted to the COMPARE website. The reference compounds with most similar GI_{50} profile across the NCI60 cell lines, defined as the highest Pearson correlation coefficient (Pearson's ***r***). The underlying assumption is that two compounds which are identified as sharing a similar NCI60 profile, they may have a similar mechanism of action.

Table S6: Standard COMPARE analysis of compound **33** based on GI_{50} mean graph [a]

Rank	NSC Number	Mechanism of Action	Compound	Correlation Coefficient	Reference
1	791946	Tubulin polymerization inhibitor	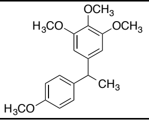	0.85	[5]
2	791947	Cytotoxicity, tubulin inhibition	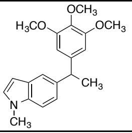	0.84	[6]
3	794223	Unknown mechanism of action	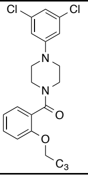	0.83	Data not available
4	792769	Inhibition of colchicine binding site of tubulin.	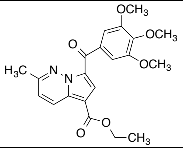	0.83	[7]
5	792957	Antiproliferative and antiapoptotic activity, tubulin binding site inhibition	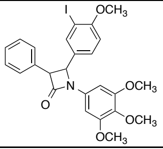	0.83	[8]
6	791526	Bisindole alkaloid binds to the microtubule proteins of the mitotic spindle, mitotic arrest and cell death	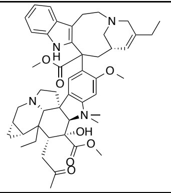	0.81	[9]
8	31708	Antimitotic action	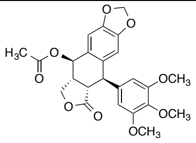	0.80	[10]
7	799732	Unknown mechanism of action	Structure not available	0.81	Data not available
9	705323	Unknown mechanism of action	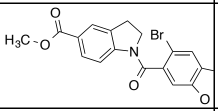	0.80	[11]
10	782882	Induction of mitotic arrest and apoptosis	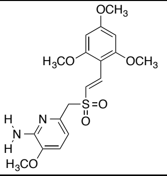	0.80	[12]
11	736992	Interaction with the colchicine binding site of αβ-tubulin interface.	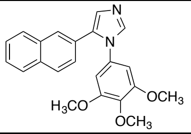	0.79	[13]
12	781551	Microtubule disruption	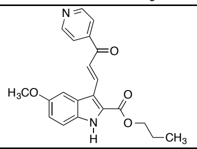	0.79	[14]

13	782361	Microtubule depolymerizing and antitumor activity	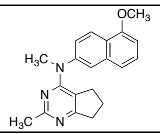	0.78	[15]
14	794655	Unknown mechanism of action	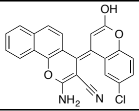	0.78	Data not available
15	792961	Antiproliferative and antiapoptotic activity	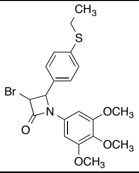	0.76	[16]
16	794658	Unknown mechanism of action	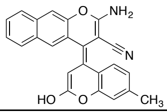	0.76	Data not available
17	747379	Caspase activators and apoptosis inducers	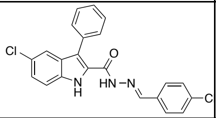	0.76	[17]
18	794639	Tubulin polymerization inhibition, targets at the colchicine-binding site	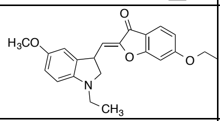	0.76	[18]
19	791945	Unspecified anticancer activity	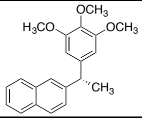	0.75	[19]

[a]The target set was the NCI synthetic database and the target set endpoints were selected to be equal to the seed end points. Standard COMPARE analysis was performed. Correlation values (r) are Pearson correlation coefficients.[Reference National Cancer Institute Biological Testing Branch; National Cancer Institute; Bethesda, MD; https://dtp.cancer.gov (accessed 02/03/2022)].

Table S7: Stability study for compounds **33** and **39**[a]

Conditions	Compound **33** (% remaining)	Compound **39** (% remaining)
pH 4	76	39
pH 7.4	87	50
pH 11	26	19
Heat (60°C)	90	82
Light (UV)	88	61
0.1 M HCl	60	54
0.1 M NaOH	65	60
3% H_2O_2	65	50

[a] The stability study for compounds **33** and **39** was performed by analytical HPLC, [Symmetry® column (C18, 5 mm, 4.6 x150 mm), Dual Wavelength Absorbance detector (Waters 2487), binary HPLC pump (Waters 1525), and Autosampler (Waters 717 plus)], with mobile phase acetonitrile (70%)/water (30%), flow rate 1 mL/min over 15 min and detection at λ 254 nm. Stock solutions of the compounds **33** and **39** (1 mg/mL in mobile phase) were used. Dilutions of 0.5 mg/mL, 0.25 mg/mL, 0.125 mg/mL, 0.0625 mg/mL, 0.03125 mg/mL, 0.015625 and 0.0078 mg/mL were prepared for the calibration curve.

Table S8: Docking Scores for compounds **32**, **33**, **42** and **43**

**Compound ID**	**Score**[a]
**32**(3*S*4*S*)	-9.06
**43**(4*R*)	-9.04
**42**(4*S*)	-9.03
**33**(3*S*4*S*)	-8.90
**43**(4*S*)	-8.88
**32**(3*R*4*R*)	-8.47
**33**(3*R*4*R*)	-8.36
**42**(4*R*)	-8.22

[a]Scores from docking with MOE of the best ranked pose of enantiomers of each compound **32**, **33**, **42** and **43** using the MMFF94x force field

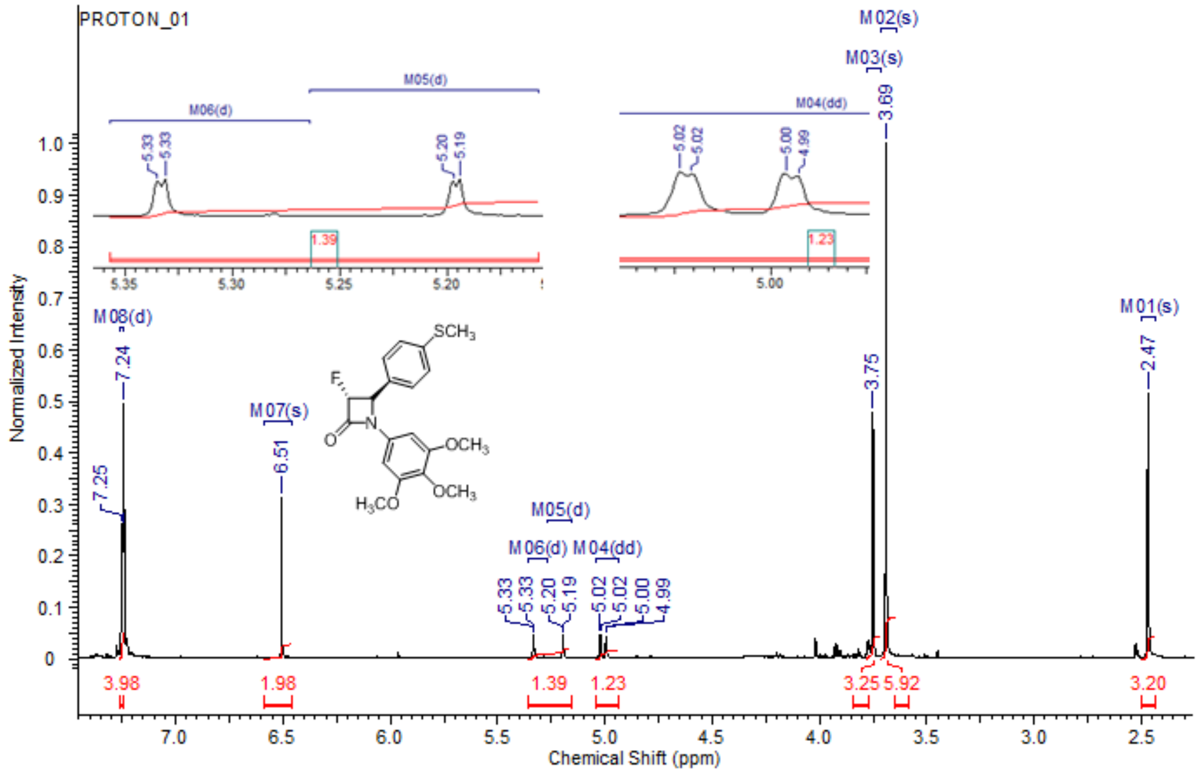

Figure S1: ^{1}H NMR spectrum of compound **28** ($CDCl_3$)

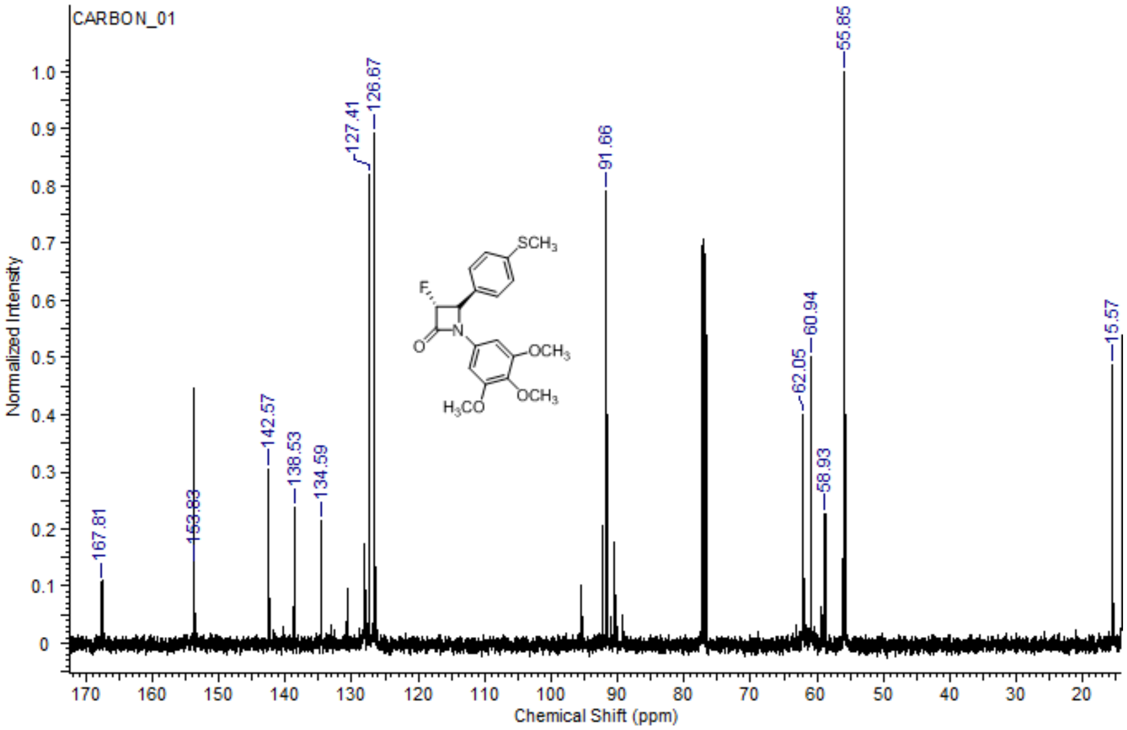

Figure S2: ^{13}C NMR spectrum of compound **28** ($CDCl_3$)

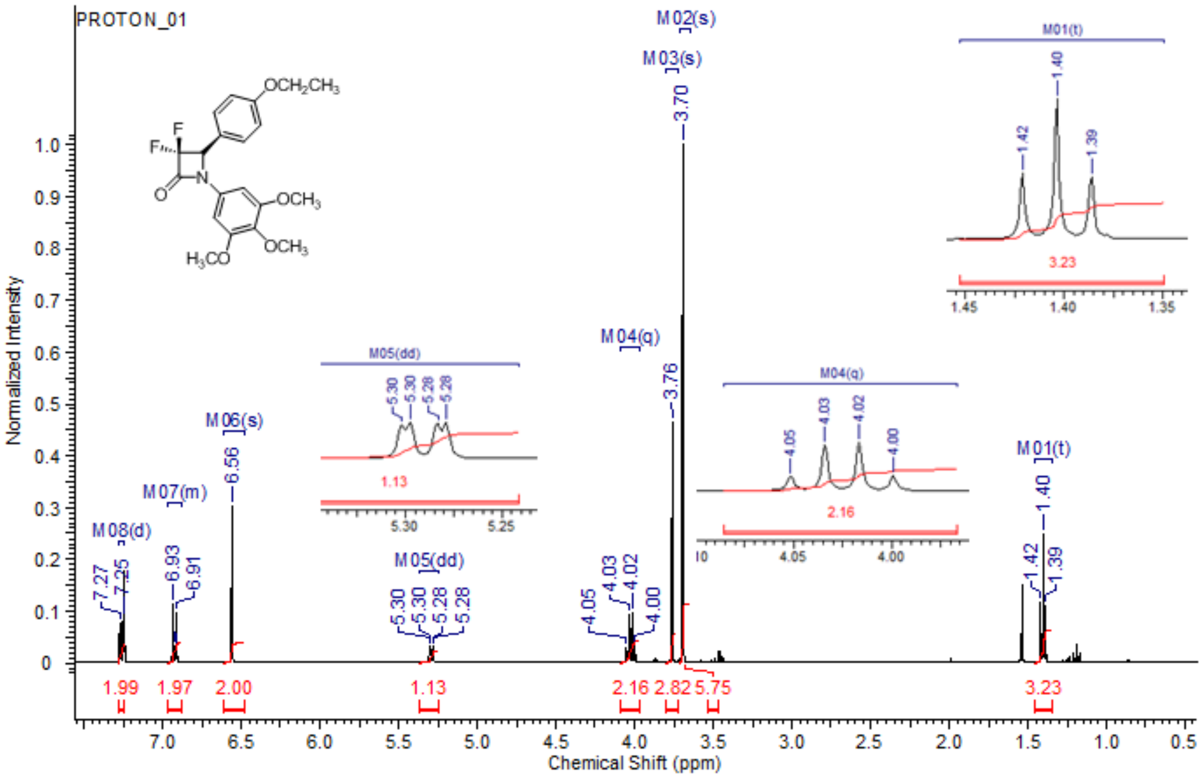

Figure S3: ^{1}H NMR spectrum of compound **37** ($CDCl_3$)

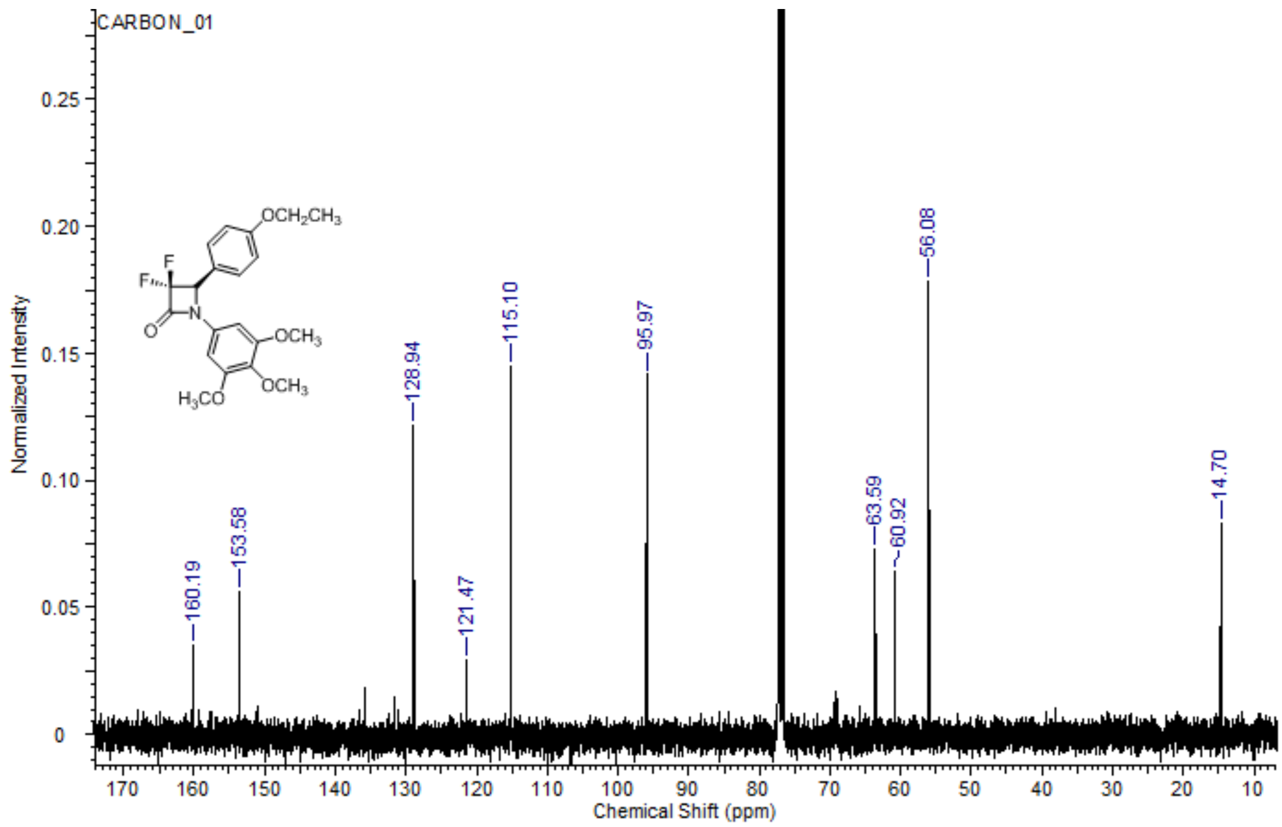

Figure S4: ^{13}C NMR spectrum of compound **37** ($CDCl_3$)

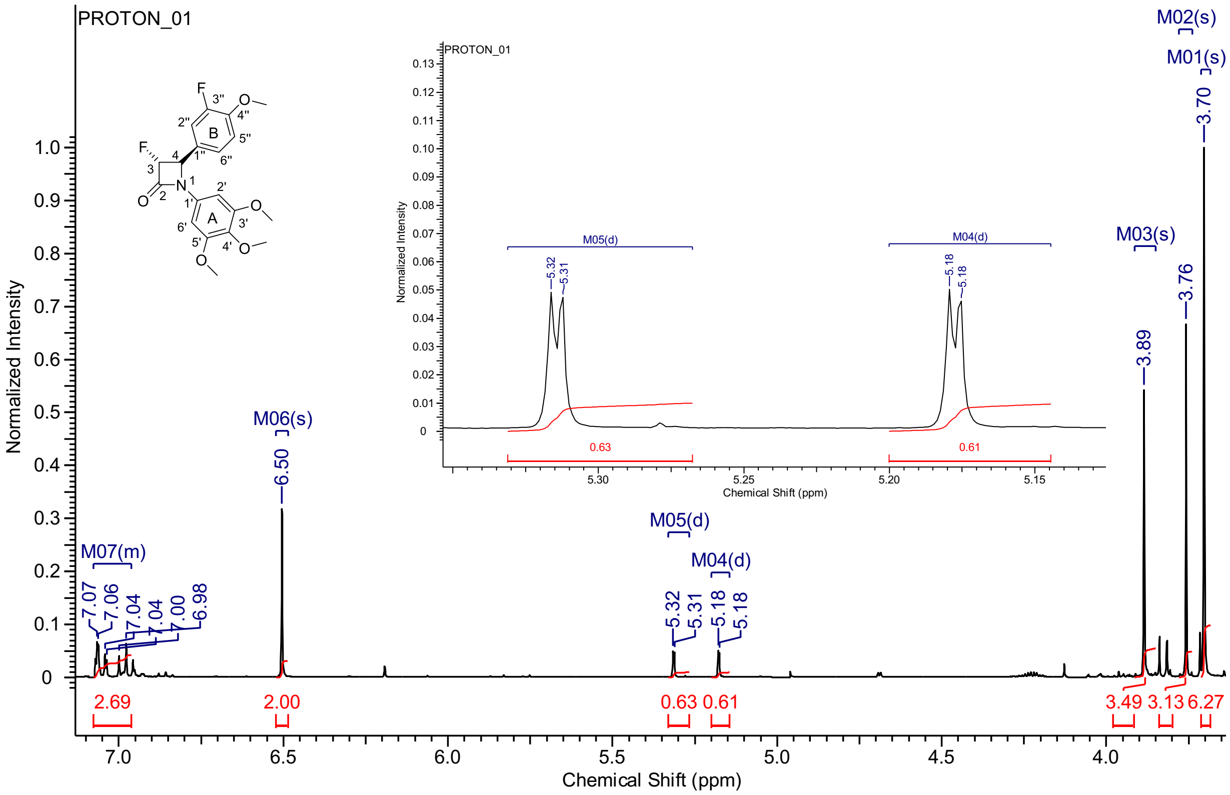

Figure S5: ^{1}H-NMR spectrum of compound **33** ($CDCl_3$)

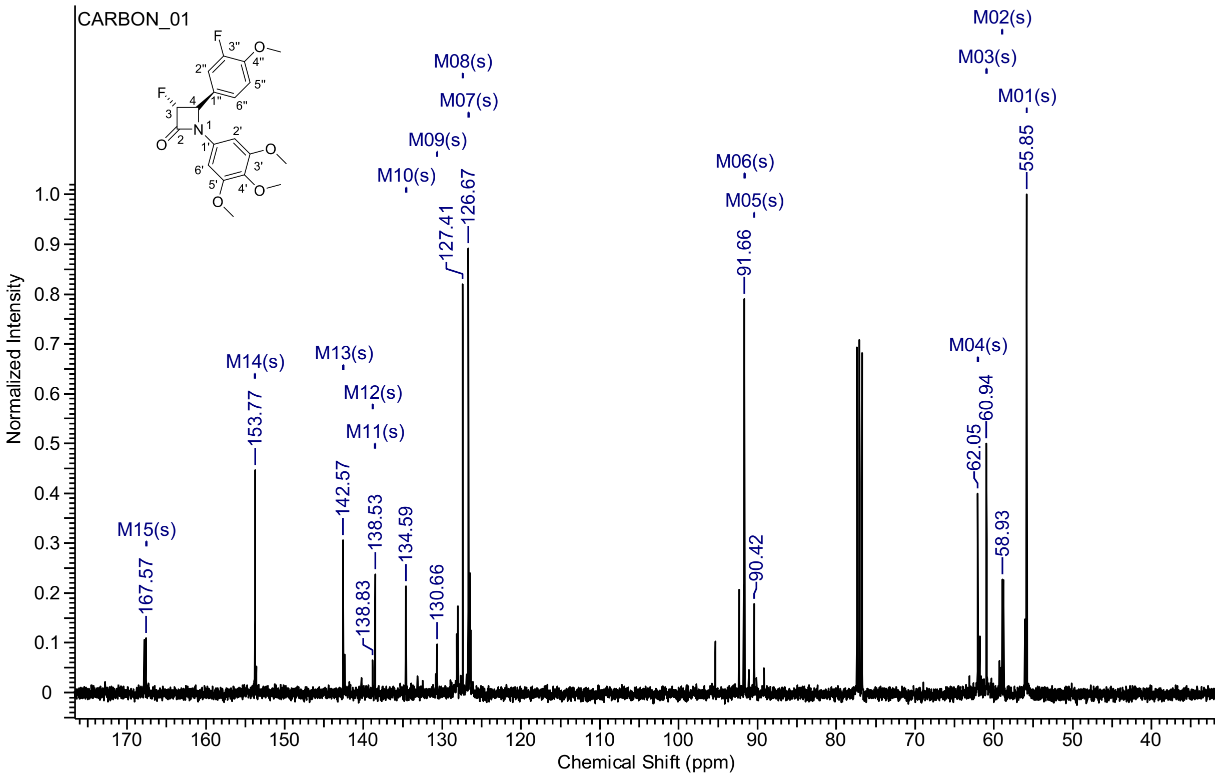

Figure S6: ^{13}C-NMR spectrum of compound **33** ($CDCl_3$)

Figure S7: ^{1}H-NMR spectrum of compound **39** ($CDCl_3$)

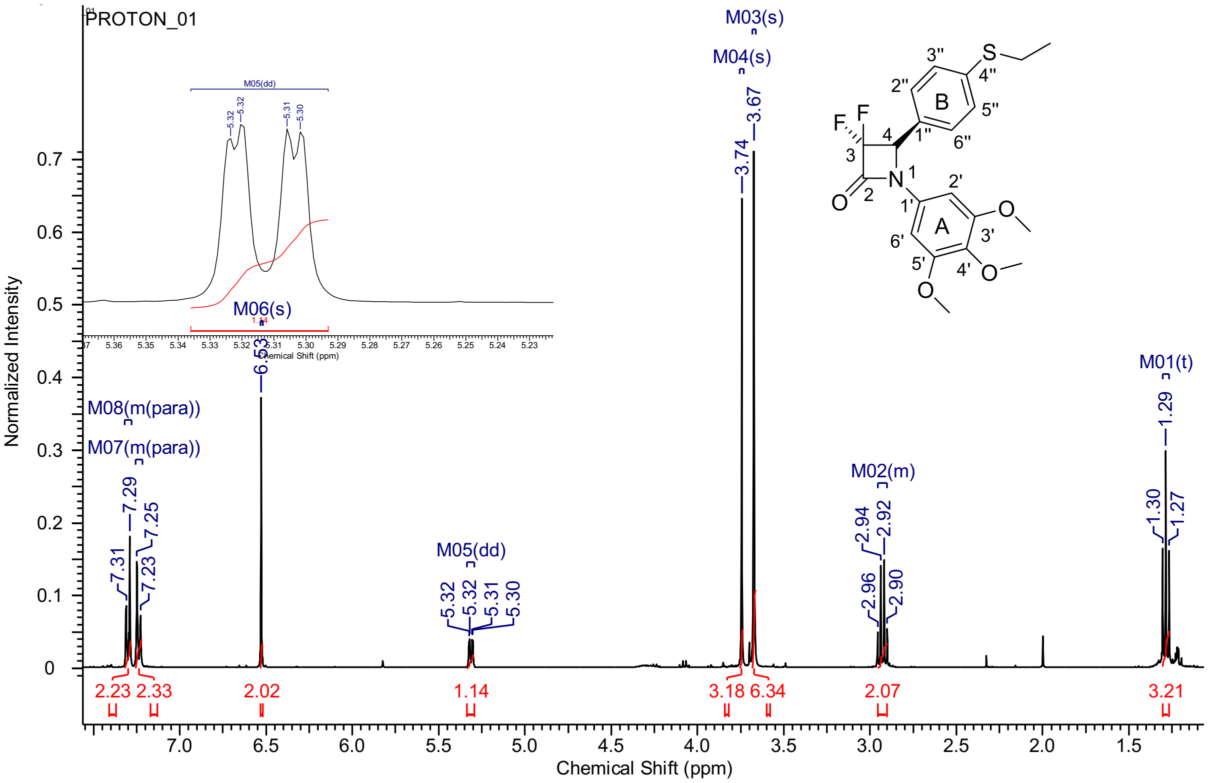

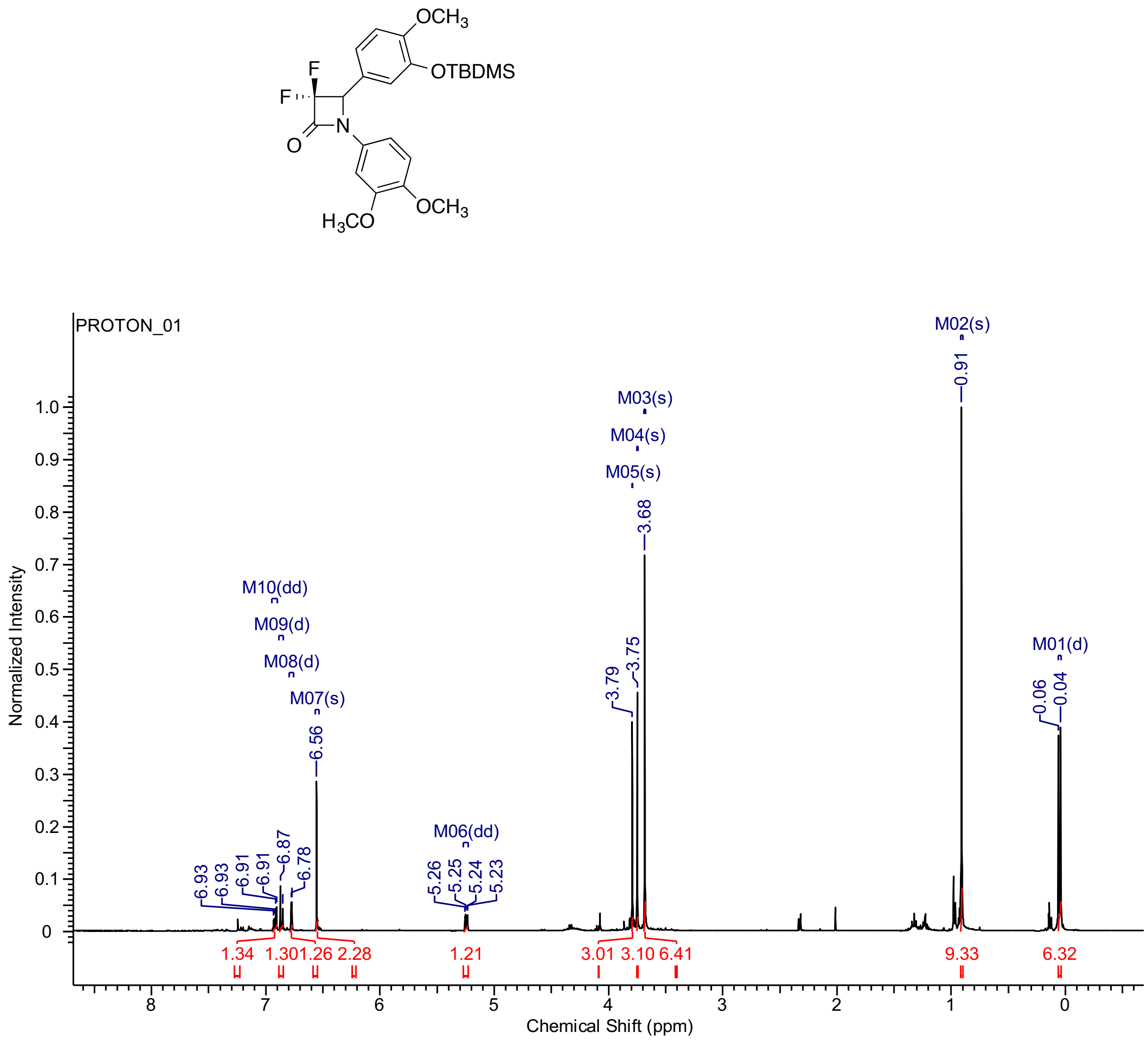

Figure S8: ^{1}H-NMR spectrum for compound **41** ($CDCl_3$)

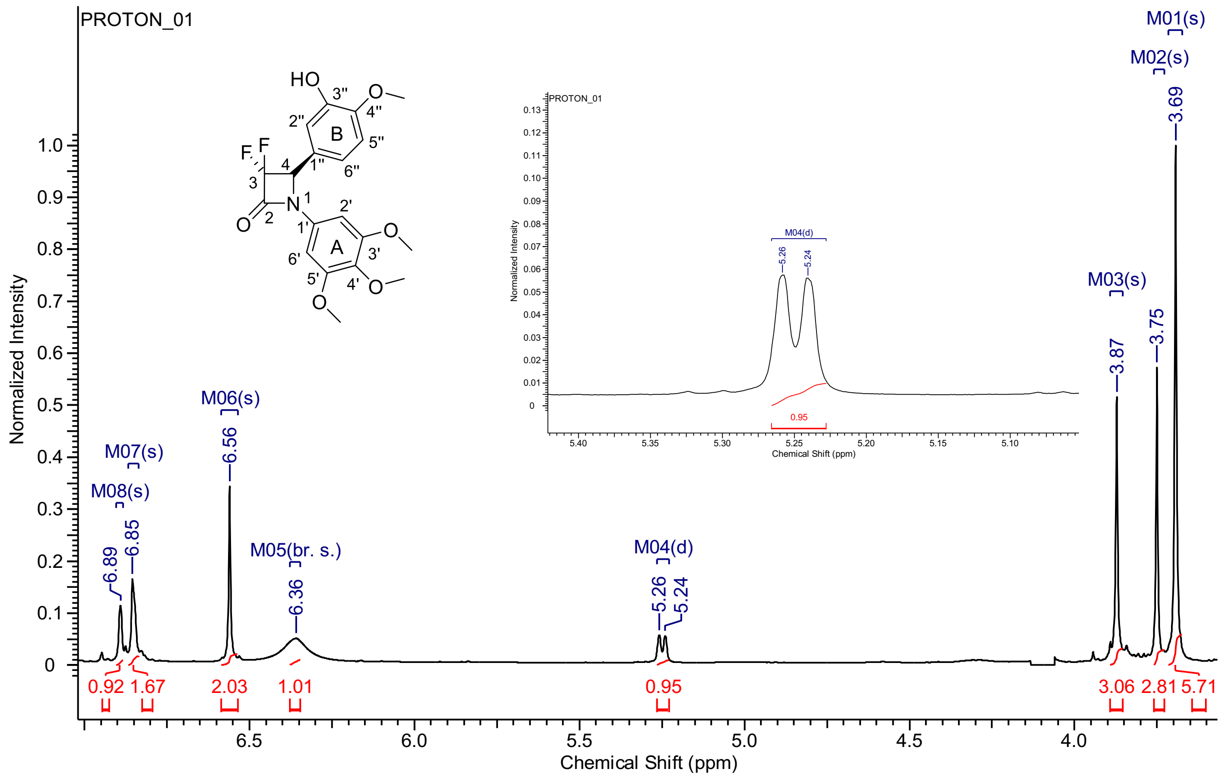

Figure S9: ^{1}H-NMR spectrum of compound **42** ($CDCl_3$)

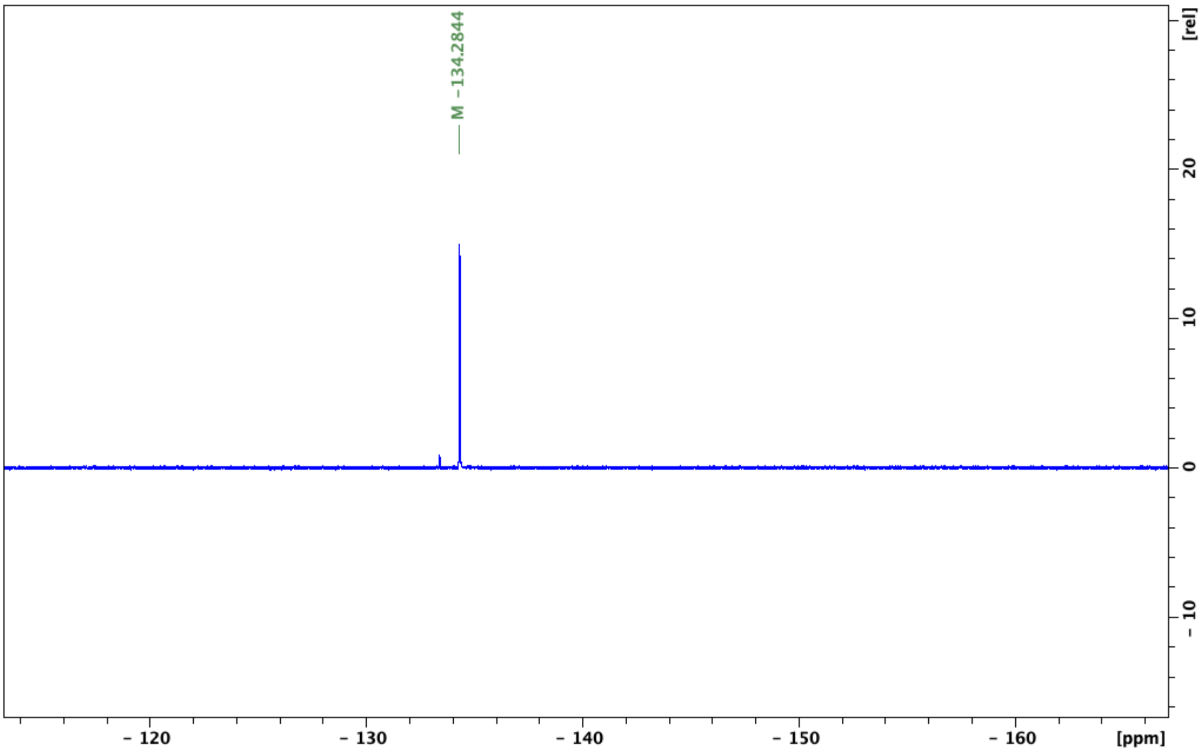

Figure S10: ^{19}F NMR spectrum of compound **23** (376 MHz, $CDCl_3$)

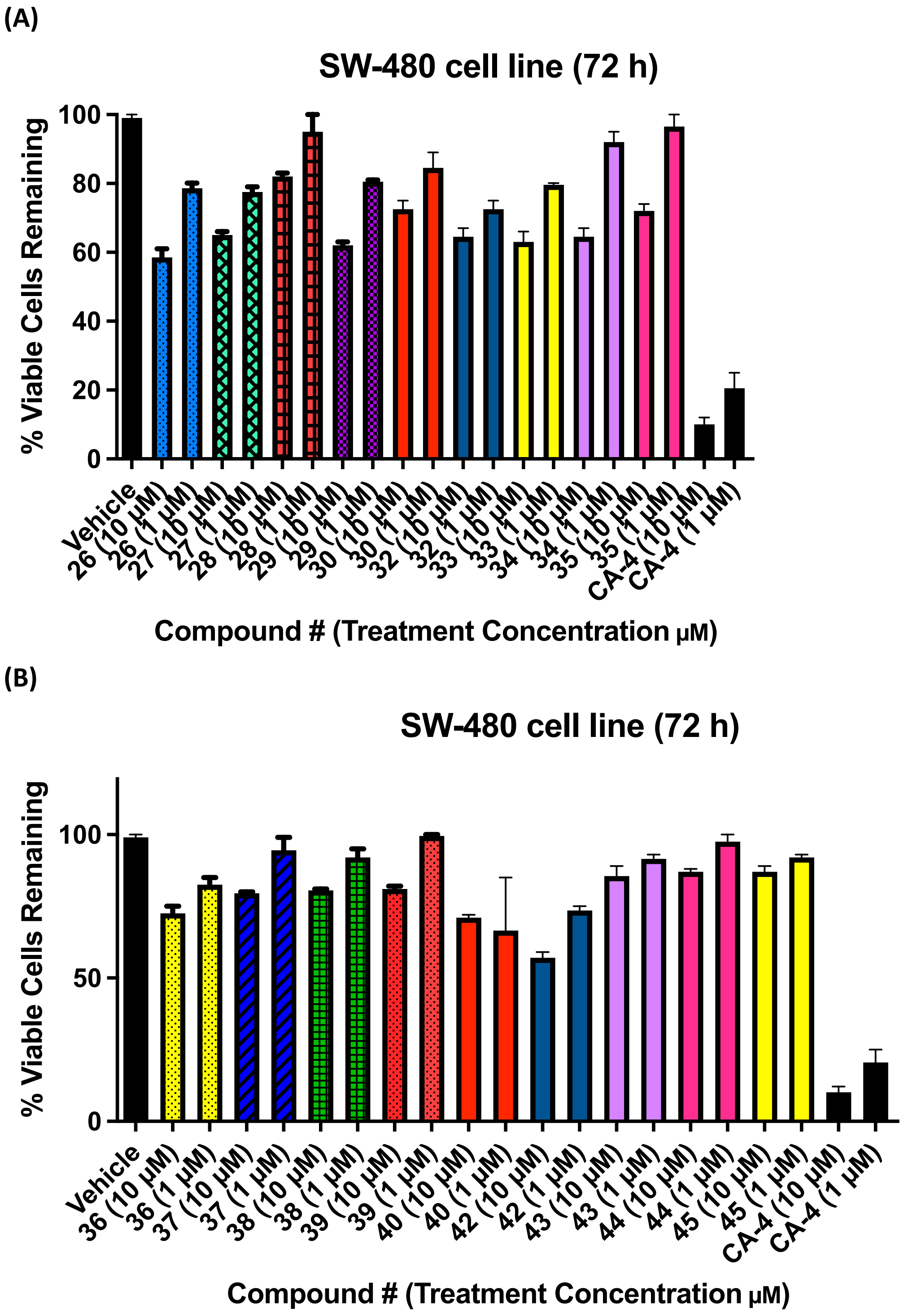

Figure S11: Preliminary cell viability data for **(A)** 3-fluoro β-lactam compounds containing different *para*-substituent ring B **26-29**; 3-fluoro β-lactam compounds containing different *meta*-substituent ring B **30**, **33-35**; **(B)** 3,3-difluoro β-lactam compounds containing different *para*-substituent ring B **36-39**; 3,3-difluoro β-lactam compounds containing different *meta*-substituent

ring B **40**, **42-45** in SW-480 cancer cells. The IC_{50} value determined for CA-4 in SW480 cells is 0.0063±0.0006 μM.

Cell proliferation of MCF-7 cells was determined with an alamarBlue assay (seeding density 2.5×10^5 cells/mL per well for 96-well plates). Compound concentrations of either 10 μM or 1 μM for 72 h were used to treat the cells with control wells containing vehicle ethanol (1% *v*/*v*). The mean value for three experiments is shown.

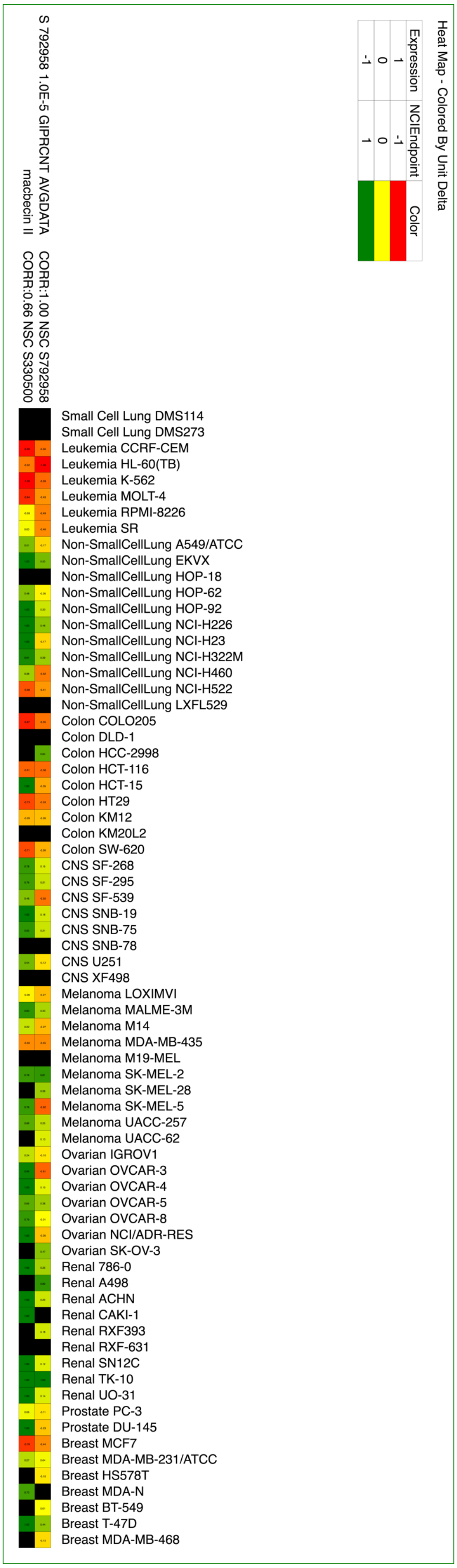

Figure S12: Heat map compound 792959 and macbeccin NSC S330500

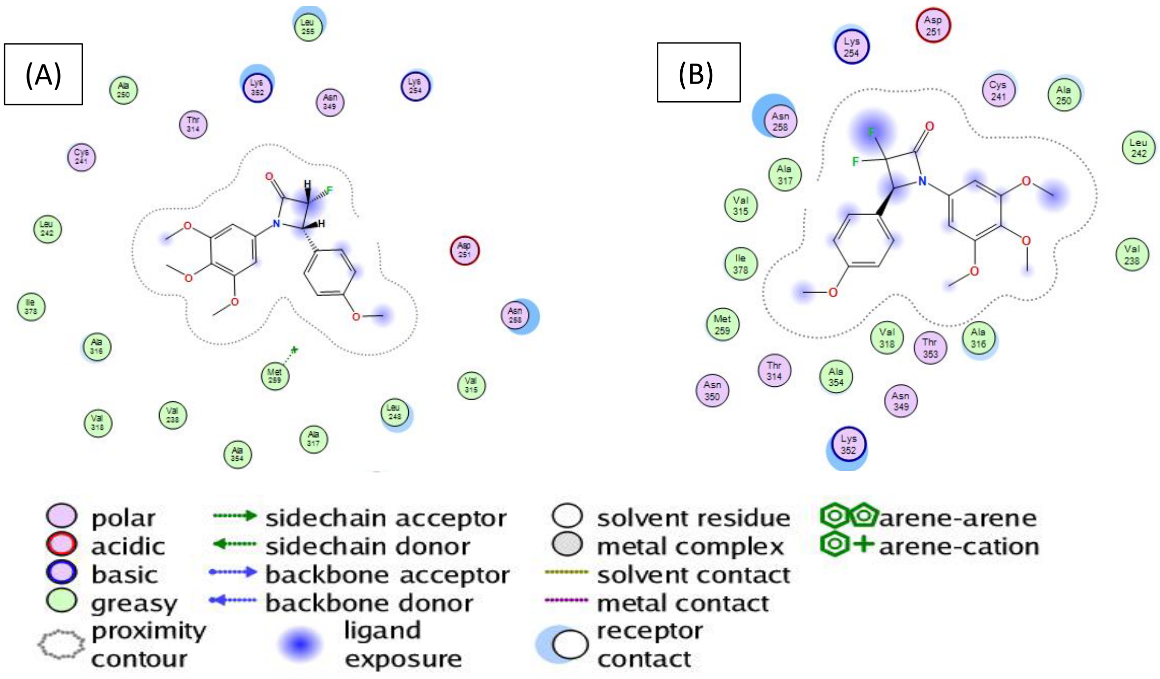

Figure S13. Protein-ligand interactions for the 3-fluoro and 3,3-difluoro-β-lactam compounds **26** and **36** with the colchicine-binding site of tubulin.

(A) 2D representation of the ligand–protein interactions of compound **26** **(red)** and Colchicine **(blue)** in the colchicine binding site interacting with Lys352, Met259, Leu 266, Lys254, Asn258 and Leu 240 rendered using the LigX module of MOE. **(B)** 2D representation of compound **36** **(red)** and Colchicine **(blue)** in the colchicine binding site interacting with Asn258, Lys254, Lys352, Ala316, Cys241 and Ala250 rendered using the LigX module of MOE.

References

1. O'Boyle, N.M.; Carr, M.; Greene, L.M.; Bergin, O.; Nathwani, S.M.; McCabe, T.; Lloyd, D.G.; Zisterer, D.M.; Meegan, M.J. Synthesis and evaluation of azetidinone analogues of combretastatin a-4 as tubulin targeting agents. *J Med Chem* **2010**, *53*, 8569-8584.
2. Carr, M.; Greene, L.M.; Knox, A.J.; Lloyd, D.G.; Zisterer, D.M.; Meegan, M.J. Lead identification of conformationally restricted beta-lactam type combretastatin analogues: Synthesis, antiproliferative activity and tubulin targeting effects. *Eur J Med Chem* **2010**, *45*, 5752-5766.
3. Cushman, M.; Nagarathnam, D.; Gopal, D.; He, H.M.; Lin, C.M.; Hamel, E. Synthesis and evaluation of analogues of (z)-1-(4-methoxyphenyl)-2-(3,4,5-trimethoxyphenyl)ethene as potential cytotoxic and antimitotic agents. *J Med Chem* **1992**, *35*, 2293-2306.
4. Messaoudi, S.; Treguier, B.; Hamze, A.; Provot, O.; Peyrat, J.F.; De Losada, J.R.; Liu, J.M.; Bignon, J.; Wdzieczak-Bakala, J.; Thoret, S., *et al.* Isocombretastatins a versus combretastatins a: The forgotten isoca-4 isomer as a highly promising cytotoxic and antitubulin agent. *J Med Chem* **2009**, *52*, 4538-4542.
5. Messaoudi, S.; Hamze, A.; Provot, O.; Treguier, B.; Rodrigo De Losada, J.; Bignon, J.; Liu, J.M.; Wdzieczak-Bakala, J.; Thoret, S.; Dubois, J., *et al.* Discovery of isoerianin analogues as promising anticancer agents. *ChemMedChem* **2011**, *6*, 488-497.
6. Alvarez, R.; Puebla, P.; Diaz, J.F.; Bento, A.C.; Garcia-Navas, R.; de la Iglesia-Vicente, J.; Mollinedo, F.; Andreu, J.M.; Medarde, M.; Pelaez, R. Endowing indole-based tubulin inhibitors with an anchor for derivatization: Highly potent 3-substituted indolephenstatins and indoleisocombretastatins. *J Med Chem* **2013**, *56*, 2813-2827.
7. Popovici, L.; Amarandi, R.M.; Mangalagiu, II; Mangalagiu, V.; Danac, R. Synthesis, molecular modelling and anticancer evaluation of new pyrrolo[1,2-b]pyridazine and pyrrolo[2,1-a]phthalazine derivatives. *J Enzyme Inhib Med Chem* **2019**, *34*, 230-243.
8. Malebari, A.M.; Fayne, D.; Nathwani, S.M.; O'Connell, F.; Noorani, S.; Twamley, B.; O'Boyle, N.M.; O'Sullivan, J.; Zisterer, D.M.; Meegan, M.J. Beta-lactams with antiproliferative and antiapoptotic activity in breast and chemoresistant colon cancer cells. *Eur J Med Chem* **2020**, *189*, 112050.
9. Keglevich, P.H., L.; Dubrovay, Z.; Dékány, M.; Szántay, C.; Kalaus, G.,; Szántay, C. . Bisindole alkaloids condensed with a cyclopropane ring, part 1. 14,15-cyclopropanovinblastine and vincristine. *Heterocycles* **2014** *89*, 653-668.
10. Mojica, M.A.L., A.; Rojas-Sepulveda, A. M.; Marquina, S.; Mendieta-Serrano, M. A.; Salas-Vidal, E.; Villarreal, M. L.; Alvarez, L. . Aryldihydronaphthalene-type lignans from bursera fagaroides var. Fagaroides and their antimitotic mechanism of action *Rsc Adv* **2016**, *6*, 4950-4959.
11. Prakash, O.; Khan, F. Cluster based svr-qsar modelling for hts records: An implementation for anticancer leads against human breast cancer. *Comb Chem High Throughput Screen* **2013**, *16*, 511-521.
12. Lu, T.; Goh, A.W.; Yu, M.; Adams, J.; Lam, F.; Teo, T.; Li, P.; Noll, B.; Zhong, L.; Diab, S., *et al.* Discovery of (e)-3-((styrylsulfonyl)methyl)pyridine and (e)-2-((styrylsulfonyl)methyl)pyridine derivatives as anticancer agents: Synthesis, structure-activity relationships, and biological activities. *J Med Chem* **2014**, *57*, 2275-2291.

13. Bellina, F.; Cauteruccio, S.; Monti, S.; Rossi, R. Novel imidazole-based combretastatin a-4 analogues: Evaluation of their in vitro antitumor activity and molecular modeling study of their binding to the colchicine site of tubulin. *Bioorg Med Chem Lett* **2006**, *16*, 5757-5762.
14. Trabbic, C.J.; Overmeyer, J.H.; Alexander, E.M.; Crissman, E.J.; Kvale, H.M.; Smith, M.A.; Erhardt, P.W.; Maltese, W.A. Synthesis and biological evaluation of indolyl-pyridinyl-propenones having either methuosis or microtubule disruption activity. *J Med Chem* **2015**, *58*, 2489-2512.
15. Xiang, W.; Quadery, T.M.; Hamel, E.; Luckett-Chastain, L.R.; Ihnat, M.A.; Mooberry, S.L.; Gangjee, A. The 3-d conformational shape of n-naphthyl-cyclopenta[d]pyrimidines affects their potency as microtubule targeting agents and their antitumor activity. *Bioorganic & medicinal chemistry* **2021**, *29*, 115887.
16. Malebari, A.M.; Wang, S.; Greene, T.F.; O'Boyle, N.M.; Fayne, D.; Khan, M.F.; Nathwani, S.M.; Twamley, B.; McCabe, T.; Zisterer, D.M.*, et al.* Synthesis and antiproliferative evaluation of 3-chloroazetidin-2-ones with antimitotic activity: Heterocyclic bridged analogues of combretastatin a-4. *Pharmaceuticals (Basel)* **2021**, *14*.
17. Preparation of indole-2-carboxylic acid benzylidenehydrazides and analogs as caspase activators and apoptosis inducers; cai, s.X.; drewe, j.A.; zhang, h.; united state patent us20020091148 (2002-09-12).
18. Xie, Y.; Kril, L.M.; Yu, T.; Zhang, W.; Frasinyuk, M.S.; Bondarenko, S.P.; Kondratyuk, K.M.; Hausman, E.; Martin, Z.M.; Wyrebek, P.P.*, et al.* Semisynthetic aurones inhibit tubulin polymerization at the colchicine-binding site and repress pc-3 tumor xenografts in nude mice and myc-induced t-all in zebrafish. *Sci Rep* **2019**, *9*, 6439.
19. Taylor, B.L.; Swift, E.C.; Waetzig, J.D.; Jarvo, E.R. Stereospecific nickel-catalyzed cross-coupling reactions of alkyl ethers: Enantioselective synthesis of diarylethanes. *J Am Chem Soc* **2011**, *133*, 389-391.
